# Effects of finerenone on arterial stiffness and cardiorenal biomarkers in patients with type 2 diabetes and chronic kidney disease: a randomised placebo-controlled mechanistic trial (FIVE-STAR)

**DOI:** 10.1186/s12933-025-03014-x

**Published:** 2025-12-05

**Authors:** Atsushi Tanaka, Muthiah Vaduganathan, Takumi Imai, Yosuke Okada, Satomi Sonoda, Keiichi Torimoto, Satoru Suwa, Hiroki Teragawa, Motoaki Miyazono, Makoto Fukuda, Keisuke Yonezu, Naohiko Takahashi, Yuichi Yoshida, Kenichi Tanaka, Michio Shimabukuro, Yuki Hotta, Masao Moroi, Hiroki Niikura, Keisuke Kida, Kenichi Yokota, Daiju Fukuda, Kengo Tanabe, Yu Horiuchi, Shigeru Toyoda, Isao Taguchi, Hisako Yoshida, Toru Miyoshi, Masaomi Nangaku, Hirotaka Shibata, Koichi Node

**Affiliations:** 1 Department of Cardiovascular Medicine, Saga University, Saga, Japan; 2 Division of Cardiovascular Medicine, Brigham and Women’s Hospital, Harvard Medical School, Boston, MA USA; 3 Clinical Research Division, Organization for Clinical Medicine Promotion, Tokyo, Japan; 4 Clinical and Translational Research Center, Kobe University Hospital, Kobe, Japan; 5 Clinical Research Center, Hospital of the University of Occupational and Environmental Health, Kitakyushu, Japan; 6 First Department of Internal Medicine, University of Occupational and Environmental Health, Kitakyushu, Japan; 7 Department of Cardiology, Juntendo University Shizuoka Hospital, Izunokuni, Japan; 8 Department of Cardiovascular Medicine, JR Hiroshima Hospital, Hiroshima, Japan; 9 Department of Nephrology, Saga University, Saga, Japan; 10 Department of Cardiology and Clinical Examination, Faculty of Medicine, Oita University, Yufu, Japan; 11 Department of Endocrinology, Rheumatology and Nephrology, Faculty of Medicine, Oita University, Metabolism, Yufu, Japan; 12 Wakamatsu Hospital of the University of Occupational and Environmental Health, Kitakyushu, Japan; 13 Department of Diabetes, Endocrinology, and Metabolism, Fukushima Medical University School of Medicine, Fukushima, Japan; 14 Division of Cardiovascular Medicine, Toho University Ohashi Medical Center, Tokyo, Japan; 15 Department of Pharmacology, St. Marianna University School of Medicine, Kawasaki, Japan; 16 Division of Metabolism and Endocrinology, Department of Internal Medicine, St. Marianna University School of Medicine, Kawasaki, Japan; 17 Department of Cardiovascular Medicine, Osaka Metropolitan University Graduate School of Medicine, Osaka, Japan; 18 Division of Cardiology, Mitsui Memorial Hospital, Tokyo, Japan; 19 Department of Cardiovascular Medicine, Dokkyo Medical University, Mibu, Japan; 20 Department of Cardiology, Dokkyo Medical University Saitama Medical Center, Koshigaya, Japan; 21 Department of Medical Statistics, Osaka Metropolitan University Graduate School of Medicine, Osaka, Japan; 22 Department of Cardiovascular Medicine, Dentistry and Pharmaceutical Sciences, Okayama University Graduate School of Medicine, Okayama, Japan; 23 Division of Nephrology and Endocrinology, The University of Tokyo Graduate School of Medicine, Tokyo, Japan

**Keywords:** Finerenone, Vascular stiffness, Biomarker, Type 2 diabetes, Chronic kidney disease, Proteomics

## Abstract

**Background:**

The mechanisms underlying cardiorenal benefits of finerenone remain unclear. This mechanistic trial aimed to evaluate the effects of finerenone on vascular stiffness, as assessed using the cardio-ankle vascular index (CAVI), and cardiorenal biomarkers in patients with type 2 diabetes (T2D) and chronic kidney disease (CKD).

**Methods:**

Eligible patients with T2D and CKD (estimated glomerular filtration rate [eGFR], 25 to < 90 mL/min/1.73 m^2^; urinary albumin-to-creatinine ratio [UACR], 30 to < 3500 mg/g Cr) were randomly allocated to receive either dose-adjusted finerenone or matching placebo. The primary endpoint was the change in CAVI at week 24. The key secondary endpoint was the proportional change in UACR from baseline over 24 weeks. As an exploratory analysis, changes in circulating proteins were measured by using the Olink® Target 96 Cardiovascular III and Inflammation panels.

**Results:**

This investigator-initiated, multicentre, prospective, two-arm parallel, placebo-controlled, double-blind, randomised clinical trial was conducted at 13 sites in Japan. Among 102 patients randomised, 101 (66.3% men; median age, 73 years; eGFR, 56.2 mL/min/1.73 m^2^; and UACR, 193.8 mg/g Cr) were analysed. Changes in CAVI at week 24 were − 0.023 (95% confidence interval [CI], − 0.299 to 0.254) for finerenone and 0.011 (95% CI, − 0.245 to 0.267) for placebo. The group difference was − 0.057 (95% CI, − 0.428 to 0.314; *P* = 0.760). Compared with placebo, finerenone led to a 29% reduction in UACR levels at weeks 12 (group ratio 0.706 [95% CI, 0.504 to 0.989; *P* = 0.043]) and 24 (0.709 [95% CI, 0.506 to 0.994; *P* = 0.046]). Finerenone also resulted in an early and sustained eGFR decline over 24 weeks, without increasing levels of urinary biomarkers of acute tubular injury. Finerenone, compared with placebo, was associated with nominal changes in the expression of 11 proteins among the 181 circulating proteins tested.

**Conclusions:**

Finerenone did not affect changes in vascular stiffness but led to a significant and sustained reduction in albuminuria in patients with T2D and CKD. The clinical benefits of finerenone may result from lowering intraglomerular pressure rather than from its effect on vascular stiffness.

**Registration:**

ClinicalTrial.gov (NCT05887817) and Japan Registry of Clinical Trials (jRCTs021230011).

**Graphical abstract:**

This mechanistic clinical trial involving patients with T2D and CKD found that 24-week finerenone therapy did not significantly reduce CAVI from baseline compared with placebo (−0.057; 95% CI, −0.428 to 0.314), led to a 29% reduction (group ratio [finerenone vs. placebo] of 0.71) in UACR levels, and was associated with nominal changes in 11 circulating proteins (six upregulated and five downregulated) over 24 weeks. CAVI, cardio-ankle vascular index; CI, confidence interval; CKD, chronic kidney disease; eGFR, estimated glomerular filtration rate; T2D, type 2 diabetes; UACR, urinary albumin-to-creatinine ratio.
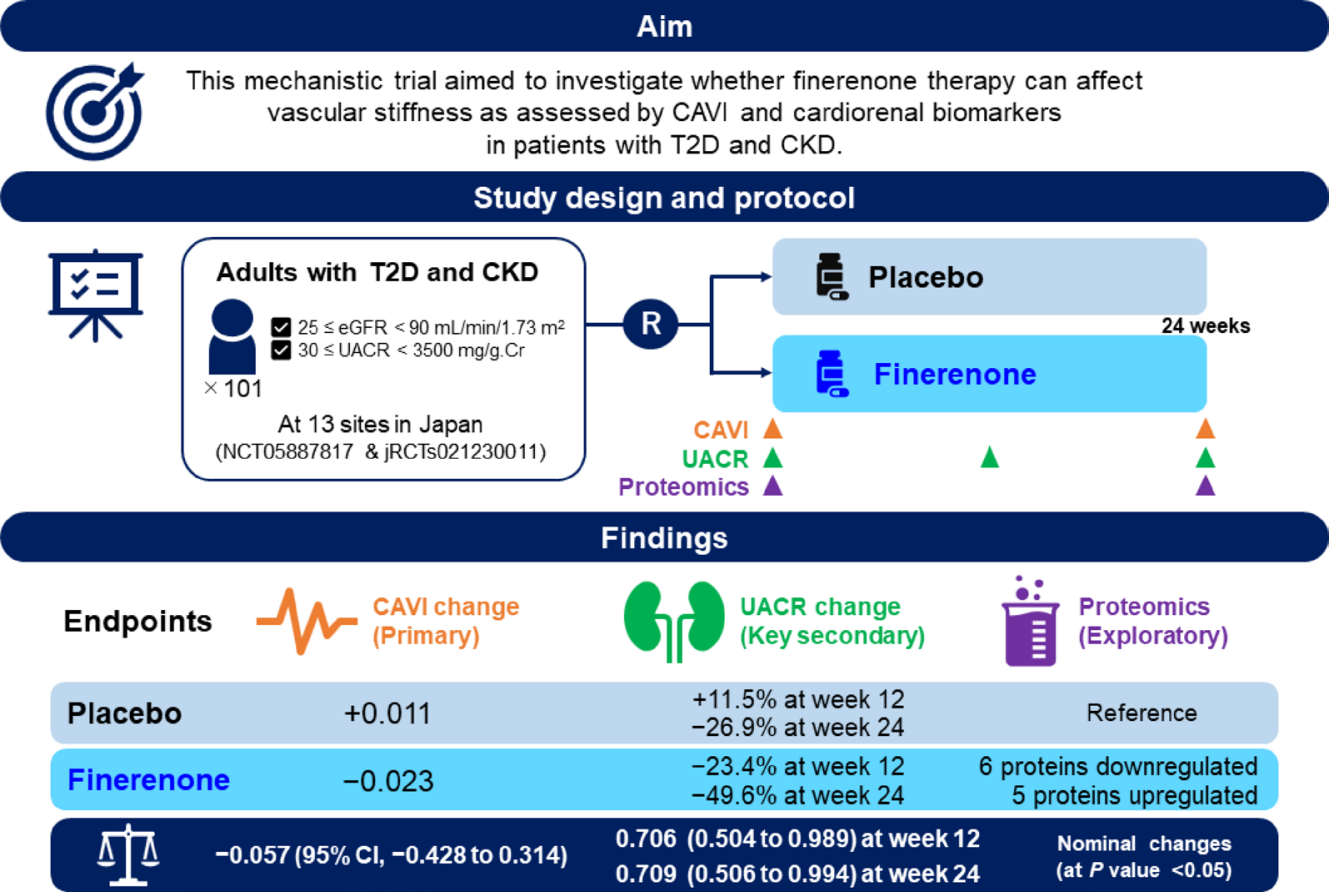

**Supplementary Information:**

The online version contains supplementary material available at 10.1186/s12933-025-03014-x.

## Research insights

**What is currently known about this topic?**

Overactivation of the mineralocorticoid receptor can cause cardiovascular and kidney injuries. Finerenone, a nonsteroidal mineralocorticoid receptor antagonist, has been shown to improve cardiorenal outcomes in patients with T2D and CKD; however, limited data are available regarding the mechanisms of cardiorenal clinical benefits of finerenone.

**What is the key research question?**

Can finerenone therapy affect the cardio-ankle vascular index, a physiological arterial stiffness marker, and relevant cardiorenal pathological status as assessed by blood and urine biomarkers in patients with T2D and CKD.

**What is new?**

Compared with placebo, 24-week finerenone therapy did not significantly affect the cardio-ankle vascular index, but significantly reduced albuminuria over 24 weeks in patients with T2D and CKD. The therapy also caused an early eGFR dip without evidence of kidney injury. Additionally, the therapy was associated with nominal changes in the expression of several systemic biomarkers from the Olink^®^ target 96 Cardiovascular III and Inflammation panels.

**How might this study influence clinical practice?**

This mechanistic study suggests that clinical benefits of finerenone likely result from lowering intraglomerular pressure rather than from its effect on vascular stiffness in this patient population. Proteomic biomarkers may help elucidate the non-haemodynamic pathways underlying finerenone therapy.

## Introduction

Cardiovascular and renal outcomes in patients with type 2 diabetes (T2D) and chronic kidney disease (CKD) remain poor despite the use of standard pharmacotherapy, including angiotensin-converting enzyme inhibitors, angiotensin-receptor blockers, and sodium-glucose cotransporter 2 (SGLT2) inhibitors [1, 2]. This suggests residual pathological targets not covered by existing medications.

Finerenone is a non-steroidal selective mineralocorticoid receptor (MR) antagonist. In the FIDELIO-DKD and FIGARO-DKD Phase III trials, finerenone reduced the risk of cardiovascular and kidney events in patients with T2D across the CKD spectrum when added to standard care [3–5]. Accumulating evidence indicates that MR overactivation causes cardiovascular and renal damage by promoting pro-inflammatory and pro-fibrotic signalling in the kidney, myocardial, and vascular tissues [6–8]. Particularly, overactivation of vascular smooth muscle cell (SMC)-MR impairs SMC function and induces vascular injury, leading to stiffened and remodelled vasculatures, contributing to cardiovascular and renal disease progression [9, 10]. This suggests that blocking MR overactivation may mitigate vascular SMC dysfunction and stiffness, potentially conferring cardiovascular and renal protection. However, clinical evidence on the effects of finerenone therapy on vascular stiffness remains limited. Additionally, the effects of finerenone on cardiorenal biomarkers, which could explain the clinical benefits observed in outcome trials, remain unclear.

The Effects of Finerenone on Vascular Stiffness and Cardiorenal Biomarkers in Type 2 Diabetes and Chronic Kidney Disease (FIVE-STAR) trial was conducted to further explore the mechanisms underlying the favourable impact of finerenone on the risk of cardiovascular and renal events in patients with T2D and CKD. We hypothesised that finerenone therapy affects vascular stiffness and relevant cardiorenal pathological status in this population.

## Methods

### Study design and oversight

The FIVE-STAR was an investigator-initiated, multicentre, prospective, randomised, double-blind, placebo-controlled, parallel-group clinical trial. The rationale and design have been published previously [11], and the full protocol is presented in Supplementary Text S1. The study protocol was approved centrally by the Certified Review Board of Fukushima Medical University (no. F2023001), and the trial adhered to the principles of the Declaration of Helsinki and the Clinical Trial Act in Japan. Written informed consent was obtained from all participants. The trial is registered as NCT05887817 (https://clinicaltrials.gov/study/NCT05887817) and jRCTs021230011 (https://jrct.mhlw.go.jp/en-latest-detail/jRCTs021230011).

### Participants

Briefly, eligible patients were adults diagnosed with T2D and CKD, meeting both of the following criteria: an estimated glomerular filtration rate (eGFR) ≥ 25 to < 90 mL/min/1.73 m^2^ and elevated albuminuria, defined as a urinary albumin-to-creatinine ratio [UACR] ≥ 30 to < 3500 mg/g Cr. Patients were required to have had no changes in T2D and CKD medications in the 4 weeks preceding consent. Key exclusion criteria encompassed patients with uncontrolled T2D, serum potassium ≥ 4.9 mEq/L, symptomatic heart failure (HF) and reduced left ventricular ejection fraction (LVEF ≤ 35%), or a history of cardiovascular and renal events within the 8 weeks preceding consent. Participants were recruited at 13 sites in Japan between September 2023 and February 2024 (Supplementary Text S2).

### Study procedures

After providing consent, patients were randomly assigned (1:1) to receive once-daily oral finerenone or a matching placebo via a web-based dynamic allocation system. A minimisation method was used to balance age (< 70 or ≥ 70 years), sex, eGFR (< 45 or ≥ 45 mL/min/1.73 m^2^), and SGLT2 inhibitor use at the time of consent. All patients and trial personnel were blinded to the treatment arms. The study drug (finerenone) and placebo tablets were supplied by Bayer and were identical in appearance to maintain blinding. Patients with a baseline eGFR < 60 mL/min/1.73 m^2^ started with 10 mg of study drug, and an up-titration to 20 mg was encouraged after 4 weeks. Patients with a baseline eGFR ≥ 60 mL/min/1.73 m^2^ received initial and maintenance doses of 20 mg. Down-titration and discontinuation of the study drug were allowed according to the patient’s medical condition, such as serum potassium and eGFR levels, at the discretion of the local investigator. After initiating the study drug, patients were required, in principle, not to start new SGLT2 inhibitors or change the dosage of concomitant medications. Study visits were conducted at weeks 4, 12, and 24.

An electronic data capture system (eClinical Base, Translational Research Center for Medical Innovation, Kobe, Japan) was used for data collection and management, with personal information masked and coded. Data were monitored independently.

### Study endpoints

The primary endpoint was the change from baseline in the cardio-ankle vascular index (CAVI) at week 24. CAVI was measured at baseline and week 24 using the VaSera device (Fukuda Denshi, Co., Ltd., Tokyo, Japan), following the manufacturer’s protocol. Generally, CAVI data from both sides were averaged and used as the patient's CAVI for analysis. In cases where data from only one side were available, the available CAVI was used. When the concurrently measured ankle-brachial index was < 0.9, the CAVI data were excluded from the analysis owing to low reliability. The secondary endpoints included (1) proportional changes from baseline in the geometric means of UACR at weeks 12 and 24 (key secondary endpoint) and (2) proportional changes from baseline in the geometric means of serum pentosidine, urinary type IV collagen, α1-microglobulin, β2-microglobulin, neutrophil gelatinase-associated lipocalin, *N*-acetyl-β-d-glucosaminidase, and liver-type fatty acid-binding protein levels, corrected for urinary creatinine at week 24. As an exploratory analysis, plasma samples collected at baseline and week 24 were used to measure changes in circulating proteins using the Olink^®^ Target 96 Cardiovascular III and Inflammation panels (Olink Proteomics AB, Uppsala, Sweden). This analysis was performed according to the manufacturer’s instructions at a central laboratory (APRO Science Group/Pharma Foods International Co., Ltd., Tokushima, Japan) in a blinded manner to treatment allocation. Relative quantification was performed using log2 transformation of Olink’s own relative abundance units (normalised protein expression, NPX). A high NPX value indicated high protein concentration.

Incidences of physician-reported adverse events, including hyperkalaemia, arising after study drug initiation were also analysed as a safety endpoint.

### Statistical analysis

Details of the sample size calculation have been published elsewhere [11]. At the time of study planning, no clinical data were available on the effect of finerenone on arterial stiffness. Therefore, we referred to the secondary analysis of the EMPA-TROPISM study, in which 6-month treatment with empagliflozin, relative to placebo, improved aortic stiffness, as assessed via pulse wave velocity, with an estimated effect size of − 0.96 standardised deviation (SD). This improvement was accompanied by a reduction in some inflammatory biomarkers, as evaluated through proteomic analysis, in patients with nondiabetic HF and reduced LVEF [12]. We conservatively hypothesised that 6 months of finerenone treatment, relative to placebo, could reduce CAVI by at least − 0.6 SD (63% of the effect size in the EMPA-TROPISM) in the FIVE-STAR trial. A minimum of 50 patients per arm was required to detect this difference at a 5% significance level (two-sided) with 80% power, accounting for a 10% drop-out rate.

The statistical analysis plan (Supplementary Text S3) was developed before the database lock. All analyses were conducted according to the intention-to-treat principle. All efficacy analyses were performed in the full analysis set (FAS), excluding patients who withdrew consent, were ineligible, did not receive study drug treatment, or lacked data on the efficacy endpoint. Additionally, adverse events were analysed in the safety set, including all randomised patients who received at least one protocol treatment.

The baseline demographic and clinical characteristics of participants are presented as median (interquartile range) or number (%). For the primary analysis, linear regression was used to assess the change in CAVI at week 24, adjusted for baseline CAVI. This analysis was also conducted in prespecified subgroups based on demographic and clinical characteristics. As a post hoc sensitivity analysis for missing CAVI values, an additional analysis was performed using multiple imputation with predictive mean matching, with results combined according to Rubin’s rules. Secondary endpoints, including UACR, were analysed using mixed-effects models for repeated measures with a compound symmetry covariance structure, including treatment, time, treatment-by-time interaction, and baseline values as fixed effects, or linear regression models adjusted for baseline values. Logistic regression analysis, adjusted for UACR at randomization, was used to evaluate treatment effects on the likelihood of achieving UACR reductions of ≥ 30% or greater. Renal endpoints, plasma/serum aldosterone, and renin activity were analysed on a logarithmic scale. In the proteomic analysis, changes in the NPX of individual circulating proteins from baseline to week 24 were compared between treatment groups using Welch's t-test. The Benjamini–Hochberg method was applied to control the false discovery rate at 5%. Safety endpoints were summarised using descriptive statistics. All statistical tests were conducted using a two-sided significance level of 0.05. All statistical analyses were performed using R (version 4.4.2; R Foundation for Statistical Computing, Vienna, Austria).

## Results

### Study population, follow-up, and treatment

**Fig. 1:**
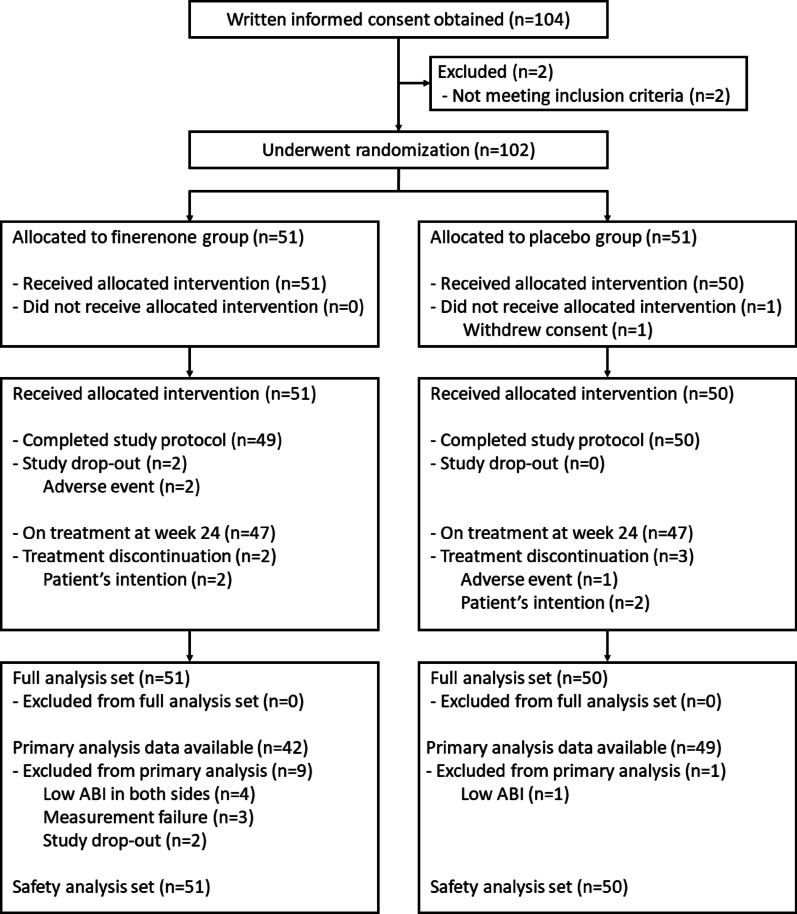
CONSORT diagram. ABI, ankle-brachial index

All participants were enrolled from September 2023 to February 2024. End-of-study visits occurred in August 2024, and database lock for the primary analysis occurred in December 2024. In total, 102 patients were equally allocated to the finerenone or placebo group (Fig. 1). A total of 101 patients (51 for the finerenone group and 50 for the placebo group) were included in the FAS, with the exception of one patient in the placebo group who withdrew consent prior to the start of study drug administration. Two patients in the finerenone group dropped out of the study owing to adverse events, and 94 patients (47 for the finerenone group and 47 for the placebo group) were on treatment at week 24. A total of 101 patients (51 for the finerenone group and 50 for the placebo group) were included in the FAS, with the exception of one patient in the placebo group who withdrew consent prior to the start of study drug administration.

Baseline patient demographic and clinical characteristics were well balanced between arms (Table 1). Overall, the median age was 73 (64, 79) years, and 67 patients (66.3%) were male. At randomisation, the median eGFR and UACR were 56.2 (44.7, 66.5) mL/min/1.73 m^2^ and 193.8 (68.0, 501.0) mg/g Cr, respectively. A distribution of patients by the Kidney Disease: Improving Global Outcomes (KDIGO) risk categories is provided in Supplementary Figure S1.

**Table 1:** Baseline patient demographic and clinical characteristics

Variable	Total (*n* = 101)	Finerenone (*n* = 51)	Placebo (*n* = 50)	Standardised mean difference between treatment groups
Age,* year	73 (64, 79)	73 (65, 80)	73 (63, 77)	0.108
Male sex	67 (66.3)	34 (66.7)	33 (66.0)	0.014
Body mass index, kg/m^2^	24.4 (22.5, 27.9)	24.2 (21.9, 27.2)	24.5 (22.9, 27.9)	0.209
Systolic blood pressure, mm Hg	129 (120, 144)	130 (122, 146)	129 (118, 141)	0.197
LVEF,* %	60 (55, 65)	59 (55, 62)	60 (55, 66)	0.147
History of hypertension	92 (91.1)	47 (92.2)	45 (90.0)	0.076
History of dyslipidemia	88 (87.1)	45 (88.2)	43 (86.0)	0.067
History of ischaemic heart disease	36 (35.6)	18 (35.3)	18 (36.0)	0.015
History of heart failure	25 (24.8)	13 (25.5)	12 (24.0)	0.035
Duration of diabetes,* year	14.1 (10.0, 22.3)	14.6 (10.3, 22.8)	13.0 (8.2, 21.5)	0.192
HbA1c,* %	6.8 (6.5, 7.4)	6.9 (6.5, 7.4)	6.7 (6.3, 7.3)	0.082
Diabetic nephropathy (aetiology of CKD)	82 (81.4)	43 (84.3)	39 (78.0)	0.162
Serum potassium,* mEq/L	4.3 (4.0, 4.6)	4.3 (4.0, 4.6)	4.3 (4.1, 4.6)	0.053
eGFR,* mL/min/1.73 m^2^	56.2 (44.7, 66.5)	55.7 (44.6, 64.2)	58.3 (44.8, 68.9)	0.106
Distribution				
≥ 60 mL/min/1.73 m^2^	44 (43.6)	20 (39.2)	24 (48.0)	0.178
45 to < 60 mL/min/1.73 m^2^	31 (30.7)	18 (35.3)	13 (26.0)	0.203
30 to < 45 mL/min/1.73 m^2^	19 (18.8)	10 (19.6)	9 (18.0)	0.041
< 30 mL/min/1.73 m^2^	7 (6.9)	3 (5.9)	4 (8.0)	0.083
UACR,* mg/g Cr	193.8 (68.0, 501.0)	221.8 (67.8, 533.1)	190.9 (76.6, 470.9)	0.078
Distribution				
30 to < 300 mg/g Cr	65 (64.4)	32 (62.7)	33 (66.0)	0.068
≥ 300 mg/g Cr	36 (35.6)	19 (37.3)	17 (34.0)	0.068
KDIGO risk category distribution				
Moderate	30 (29.7)	13 (25.5)	17 (34.0)	0.187
High	33 (32.7)	18 (35.3)	15 (30.0)	0.113
Very high	38 (37.6)	20 (39.2)	18 (36.0)	0.066
Baseline medications				
Renin-angiotensin system inhibitors	79 (78.2)	40 (78.4)	39 (78.9)	0.010
ACEI	6 (5.9)	4 (7.8)	2 (4.0)	0.163
ARB	59 (58.4)	29 (56.9)	30 (60.0)	0.064
ARNI	15 (14.9)	7 (13.7)	8 (16.0)	0.064
β-blocker	31 (30.7)	15 (29.4)	16 (32.0)	0.056
Calcium channel blocker	59 (58.4)	34 (66.7)	25 (50.0)	0.343
Diuretics	14 (13.9)	7 (13.7)	7 (14.0)	0.008
Statins	73 (72.3)	37 (72.5)	36 (72.0)	0.012
Insulin	14 (13.9)	7 (13.7)	7 (14.0)	0.008
Metformin	42 (41.6)	23 (45.1)	19 (38.0)	0.144
DPP-4 inhibitor	50 (49.5)	28 (54.9)	22 (44.0)	0.219
GLP-1 receptor agonist	32 (31.7)	14 (27.5)	18 (36.0)	0.184
SGLT2 inhibitor	65 (64.4)	32 (62.7)	33 (66.0)	0.068

Values are expressed as median (interquartile range) or n (%)

*At randomisation

*ACEI*, angiotensin converting enzyme inhibitor; *ARB*, angiotensin receptor blocker; *ARNI*, angiotensin receptor-neprilysin inhibitor; *CKD*, chronic kidney disease; *DPP-4*, dipeptidyl peptidase-4; *eGFR*, estimated glomerular filtration rate; *GLP-1*, glucagon-like peptide-1; *KDIGO*, Kidney Disease: Improving Global Outcomes; *LVEF*, left ventricular ejection fraction; *SGLT2*, sodium-glucose co-transporter 2; *UACR*, urinary albumin-to-creatinine ratio

In the FAS, 44 patients (43.6%; 21 [41.2%] in the finerenone group and 23 [46.0%] in the placebo group) received an initial daily dose of 20 mg, while the remaining patients received 10 mg. Among the 94 patients on treatment at week 24, 51 (54.3%; finerenone, n = 26; placebo, n = 25) started on 10 mg, while 41 (finerenone, 19 [73.1%]; placebo, 22 [88.0%]) were titrated up to 20 mg during the follow-up period. At week 24, 82 patients (87.2%; finerenone, 39 [83.0%]; placebo, 43 [91.5%]) were receiving 20 mg daily (Supplementary Table S1).

### CAVI

**Fig. 2:**
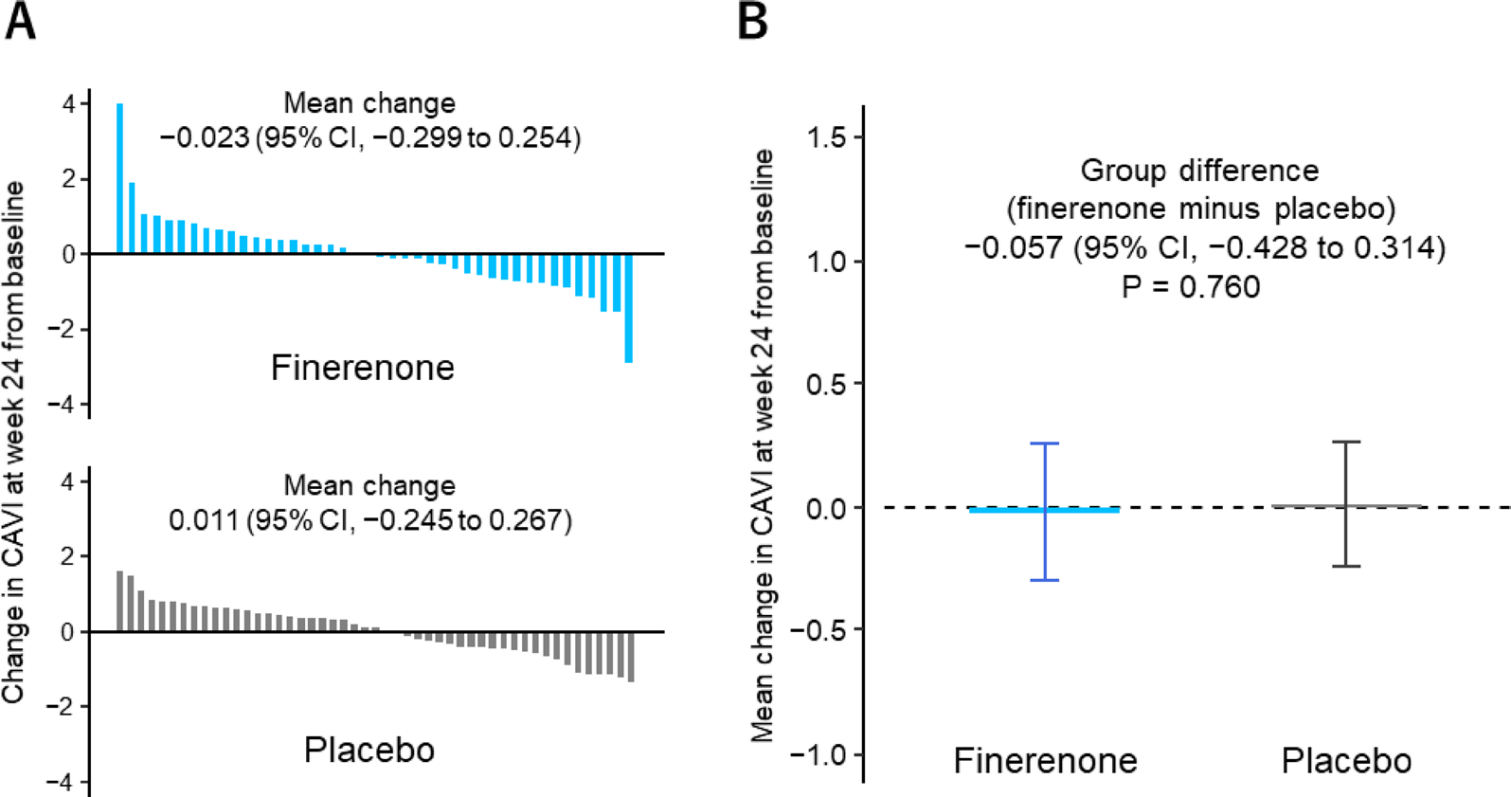
Changes in CAVI. Waterfall plots depicting individual changes in CAVI from baseline to week 24 (**A**). Comparison of changes in CAVI at week 24 from baseline in the overall study population (**B**). Group differences (finerenone minus placebo) were estimated after adjusting for baseline CAVI. CAVI, cardio-ankle vascular index; CI, confidence interval

The median baseline CAVI was 9.5 (8.3, 10.3) for the finerenone group and 9.5 (8.5, 10.4) for the placebo group. Waterfall plots showing individual changes in CAVI are presented in Fig. 2A. The estimates of CAVI change from baseline to week 24 were − 0.023 (95% CI, − 0.299 to 0.254) for the finerenone group and 0.011 (95% CI, − 0.245 to 0.267) for the placebo group. The difference in CAVI changes between the groups (finerenone minus placebo) was − 0.057 (95% CI, − 0.428 to 0.314; P = 0.760) (Fig. 2B). The treatment effect of finerenone on the primary endpoint was consistent across most prespecified subgroups (Supplementary Figure S2), but finerenone favoured the primary endpoint in subgroups with younger age (P for interaction = 0.015), lower HbA1c levels (P for interaction = 0.035), and higher LVEF (P for interaction = 0.035) (Supplementary Figures S3–5).

Although this was a double-blind trial, the finerenone group had more missing CAVI values due to low data reliability, measurement failures, and dropouts. To assess the validity of the intention-to-treat primary analysis, we conducted a sensitivity analysis using multiple imputation for missing CAVI values, which showed consistent results (group difference, − 0.061 [95% CI, − 0.477 to 0.356]; P = 0.773).

### Renal and other efficacy endpoints

**Fig. 3:**
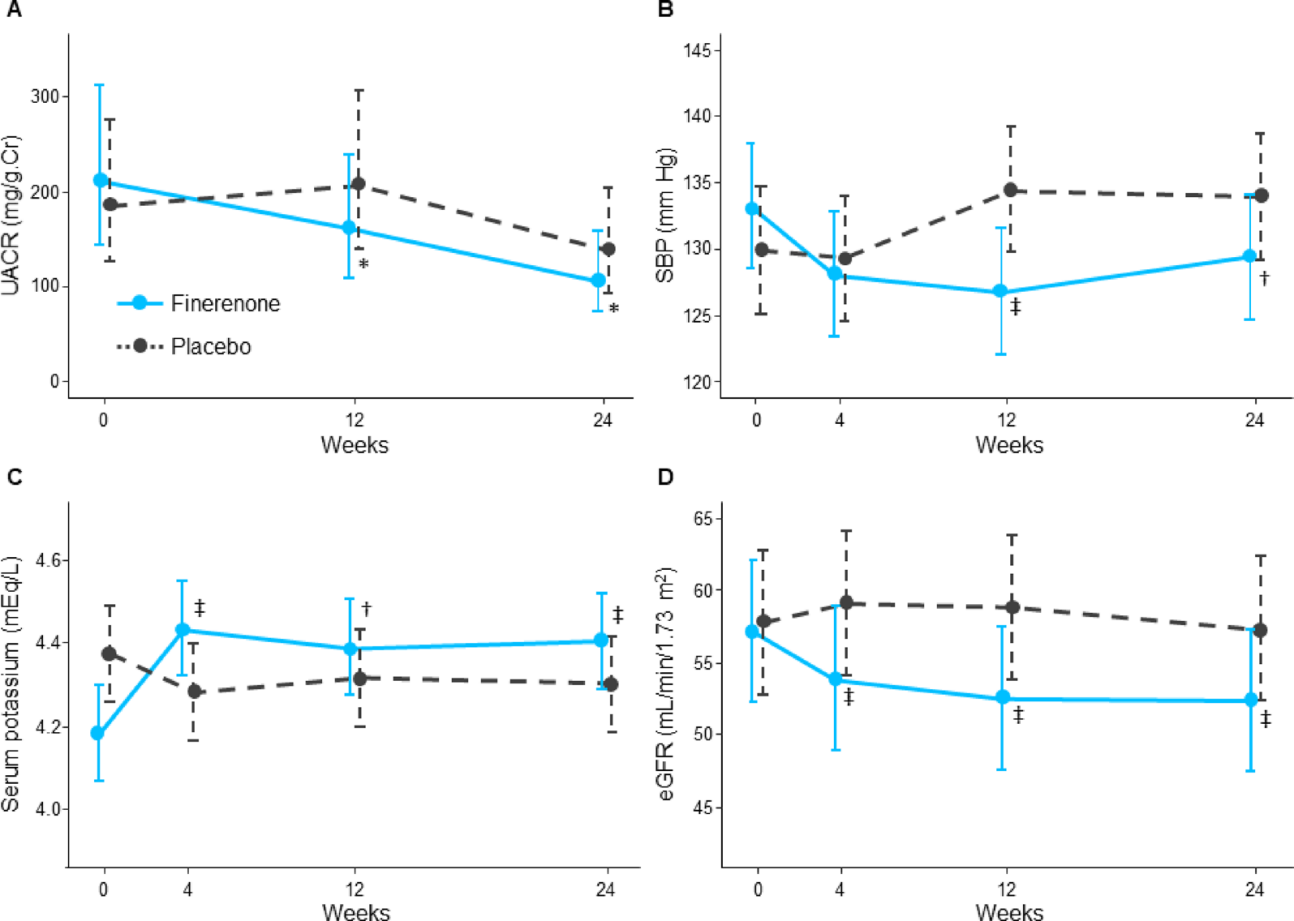
Changes in UACR (**A**), SBP (**B**), serum potassium (**C**), and eGFR (**D**) over 24 weeks. *P < 0.05 for the group ratio (finerenone vs. placebo) of the proportional change in the geometric means of UACR from baseline to weeks 12 and 24. ^†^*P* < 0.05 for the group difference (finerenone minus placebo) in the change in the corresponding parameters from baseline to weeks 12 for serum potassium and 24 for SBP. ^‡^*P* < 0.01 for the group difference (finerenone minus placebo) in the change in the corresponding parameters from baseline to weeks 4 for serum potassium and eGFR, 12 for SBP and eGFR, and 24 for serum potassium and eGFR. Detailed data on comparisons between treatment groups are shown in Table 2 (UACR) and Supplementary Table S2 (SBP, serum potassium, and eGFR). CI, confidence interval; eGFR, estimated glomerular filtration rate; SBP, systolic blood pressure; UACR, urinary albumin-to-creatinine ratio

The percent changes in UACR geometric means at weeks 12/24 were − 23.4%/ − 49.6% in the finerenone group and + 11.5%/ − 26.9% in the placebo group. The group ratios (finerenone vs. placebo) were 0.706 (95% CI, 0.504 to 0.989; P = 0.043) at week 12 and 0.709 (95% CI, 0.506 to 0.994; P = 0.046) at week 24, respectively (Fig. 3A** and **Table 2). A ≥ 30% reduction in UACR from baseline to week 24 was observed in 68.8% and 45.8% of patients treated with finerenone and placebo, respectively. Finerenone resulted in a greater odds of achieving a ≥ 30% reduction in UACR (odds ratio 2.593; 95% CI, 1.124 to 5.987; *P* = 0.026). No correlation between changes in CAVI and UACR levels was observed (Supplementary Figure S6).

**Table 2:** Changes in prespecified renal biomarkers (secondary endpoints)

Outcome and time	Finerenone	Placebo	Group ratio (finerenone vs. placebo)	
	Mean (95% CI)	Mean (95% CI)	Ratio of change (95% CI)	*P* value
UACR, mg/g Cr	(*n* = 49)	(*n* = 48)		
At baseline	211.8 (144.0 to 311.5)	186.4 (126.0 to 275.8)		
At week 12	161.5 (109.5 to 238.1)	207.5 (140.4 to 306.7)		
At week 24	106.5 (72.2 to 157.2)	137.5 (93.2 to 203.0)		
Proportional change at week 12	0.766 (0.598 to 0.981)	1.115 (0.867 to 1.434)	0.706 (0.504 to 0.989)	0.043
Proportional change at week 24	0.504 (0.393 to 0.647)	0.731 (0.570 to 0.939)	0.709 (0.506 to 0.994)	0.046
Serum pentosidine, μg/mL	(*n* = 49)	(*n* = 50)		
At baseline	0.0632 (0.0579 to 0.0690)	0.0580 (0.0531 to 0.0634)		
At week 24	0.0578 (0.0530 to 0.0631)	0.0535 (0.0491 to 0.0583)		
Proportional change at week 24	0.926 (0.849 to 1.010)	0.922 (0.846 to 1.004)	1.042 (0.936 to 1.159)	0.450
Urinary type IV collagen, μg/g Cr	(*n* = 49)	(*n* = 50)		
At baseline	9.4 (7.8 to 11.3)	8.8 (7.3 to 10.7)		
At week 24	7.6 (6.4 to 9.1)	8.4 (7.1 to 9.9)		
Proportional change at week 24	0.822 (0.713 to 0.948)	0.948 (0.823 to 1.092)	0.884 (0.742 to 1.053)	0.164
Urinary α1-microglobulin, mg/L	(*n* = 49)	(*n* = 50)		
At baseline	9.2 (7.2 to 11.6)	9.3 (7.3 to 11.8)		
At week 24	7.8 (6.3 to 9.8)	9.5 (7.6 to 11.9)		
Proportional change at week 24	0.889 (0.746 to 1.059)	1.026 (0.862 to 1.221)	0.853 (0.684 to 1.065)	0.158
Urinary β2-microglobulin, μg/L	(*n* = 49)	(*n* = 50)		
At baseline	324 (195 to 537)	258 (155 to 429)		
At week 24	175 (106 to 288)	208 (126 to 341)		
Proportional change at week 24	0.587 (0.371 to 0.930)	0.806 (0.511 to 1.271)	0.774 (0.436 to 1.375)	0.379
Urinary NGAL, μg/g Cr	(*n* = 30)	(*n* = 31)		
At baseline	54.2 (36.7 to 80.2)	50.4 (34.6 to 73.4)		
At week 24	46.9 (30.6 to 72.0)	44.5 (29.5 to 67.1)		
Proportional change at week 24	0.907 (0.674 to 1.221)	0.899 (0.672 to 1.205)	1.008 (0.667 to 1.523)	0.969
Urinary NAG, IU/L	(*n* = 49)	(*n* = 50)		
At baseline	6.6 (5.3 to 8.3)	6.2 (4.9 to 7.8)		
At week 24	6.3 (5.0 to 8.0)	6.7 (5.3 to 8.4)		
Proportional change at week 24	0.975 (0.797 to 1.192)	1.076 (0.881 to 1.314)	0.921 (0.711 to 1.192)	0.527
Urinary L-FABP, μg/g Cr	(*n* = 49)	(*n* = 50)		
At baseline	8.77 (6.54 to 11.77)	8.96 (6.66 to 12.04)		
At week 24	5.40 (4.04 to 7.22)	7.55 (5.66 to 10.06)		
Proportional change at week 24	0.634 (0.495 to 0.812)	0.843 (0.660 to 1.077)	0.739 (0.540 to 1.010)	0.058

Data are expressed as the geometric means (95% CI) or proportional change from baseline (95% CI)

*CI* confidence interval, *L-FABP* liver-type fatty acid-binding protein, *NAG* *N*-acetyl-β-d-glucosaminidase, *NGAL* neutrophil gelatinase-associated lipocalin, *UACR* urinary albumin-to-creatinine ratio

Changes in other prespecified renal biomarkers as secondary endpoints did not significantly differ between treatment groups (Table 2). Changes in other clinical and laboratory measures over 24 weeks are shown in Supplementary Table S2. Decreases in systolic blood pressure and eGFR and an increase in potassium levels were larger in the finerenone group (Fig. 3B–D). Changes in body mass index and glycated haemoglobin were similar in the two groups over 24 weeks. Finerenone therapy significantly increased the plasma/serum aldosterone concentration and renin activity at week 24.

### Proteomics

**Fig. 4:**
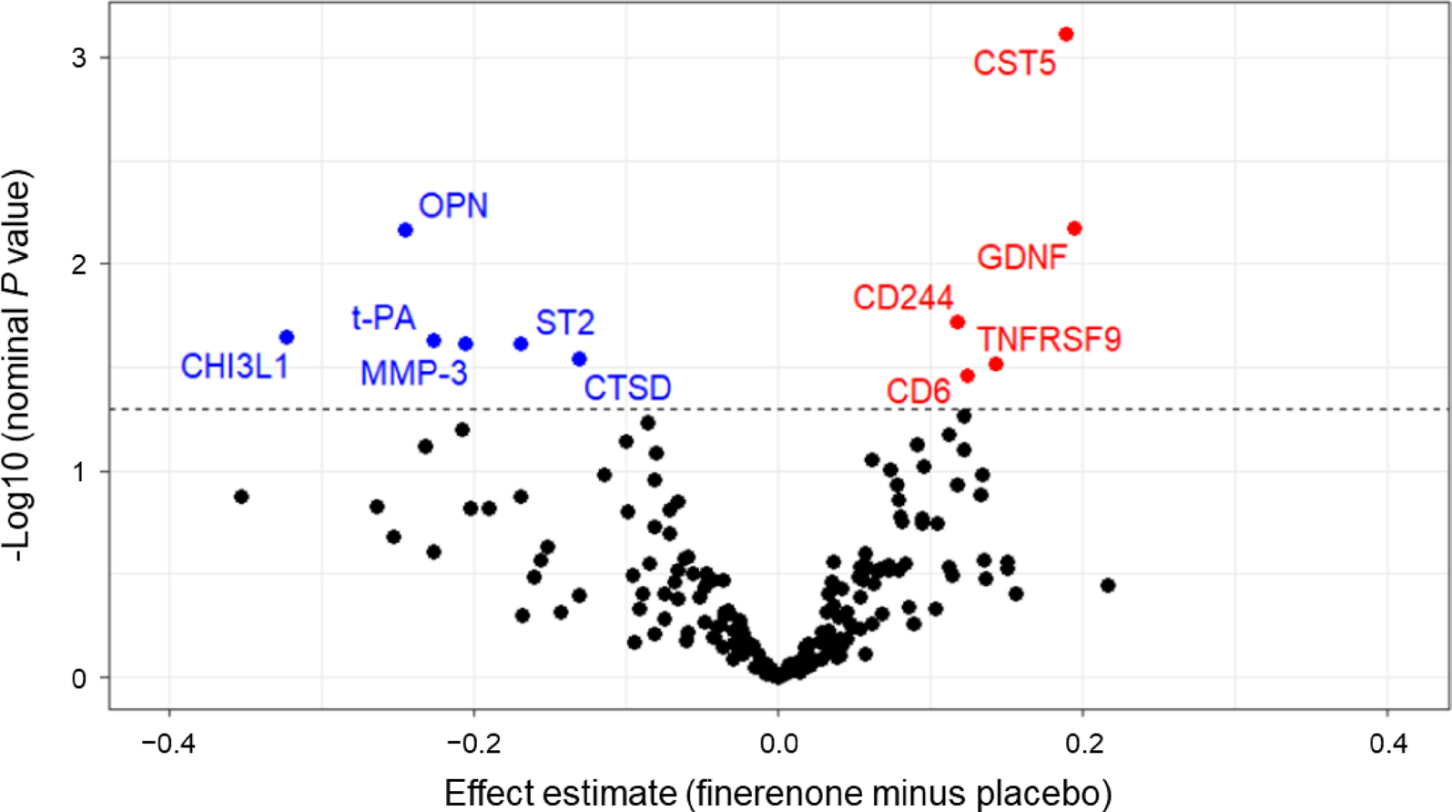
Volcano plot of the effects of finerenone on circulating cardiovascular and inflammation biomarkers. Effect estimates of finerenone versus placebo were based on the log2 fold change from baseline in normalised protein expression at week 24. Six proteins (OPN, CHI3L1, t-PA, MMP-3, ST2, and CTSD) were downregulated (blue), and five proteins (CST5, GDNF, CD244, TNFRSF9, and CD6) were upregulated (red). The horizontal dotted line indicates a nominal *P* value < 0.05. CHI3LI, chitinase-3 like-protein-1; CST5, cystatin D; CTSD, cathepsin D; GDNF, glial cell line-derived neurotrophic factor; MMP-3, matrix metalloproteinase-3; OPN, osteopontin; ST2, suppression of tumourigenesis-2; TNFRSF9, tumour necrosis factor receptor superfamily member 9; t-PA, tissue-type plasminogen activator

Among the 181 proteins in the panels (including overlapping proteins osteoprotegerin, monocyte chemotactic protein-1, and urokinase-type plasminogen activator in both panels), the finerenone group showed significant (nominal *P* value < 0.05) changes in the expression of 11 proteins, compared with the placebo group. However, these changes were not apparent after adjustment using the Benjamini–Hochberg method (Supplementary Table S3). The magnitude of statistical significance and effect estimates based on log2 fold change for each protein with finerenone therapy compared with placebo are shown in a volcano plot (Fig. 4). Compared with placebo, finerenone was associated with nominal changes in the expression of 11 proteins over 24 weeks (Supplementary Figure S7).

### Safety

The incidence of adverse events after study drug initiation, reported by local investigators, was similar between the treatment arms: serious adverse events occurred in five patients (9.8%) treated with finerenone and five patients (10.0%) treated with placebo (Supplementary Table S4). No acute kidney injury-related adverse events or cases of hyperkalaemia leading to hospitalisation or death were reported. Hypokalaemia (potassium < 3.5 mEq/L) occurred in none of the patients in the finerenone group and in 6.0% of those in the placebo group.

## Discussion

The key findings of this mechanistic clinical trial (FIVE-STAR) are as follows: (1) finerenone had no obvious effect on arterial stiffness as assessed via CAVI, (2) finerenone significantly and durably reduced albuminuria, and (3) an exploratory proteomic analysis suggested that the therapy could modify several cardiovascular and inflammatory biomarkers (Graphical Abstract).

MR overactivation, particularly in the vasculature, contributes to adverse vascular aging, remodelling, and injury, promoting cardiovascular pathologies.^10^ In an experimental rat model of CKD [13], finerenone reduced the intrinsic arterial stiffness β-index, accompanied by reduced oxidative stress and albuminuria. In a meta-analysis of randomised clinical trials [14], conventional MR antagonist therapy (excluding finerenone) significantly mitigated arterial stiffness, as assessed via pulse-wave velocity, in 11 studies involving cohorts with diverse backgrounds, relatively small sample sizes, and younger participants (mean age, 58.3 years). In contrast, the present study did not find obvious evidence to support the beneficial effect of finerenone on another arterial stiffness maker, CAVI, in the primary evaluation. The treatment effect was largely consistent across prespecified subgroups, including baseline CAVI and renal function levels and background SGLT2 inhibitor use. Although the reasons for the discrepancy with previous studies are unclear, our findings may imply that clinical benefits observed in recent cardiovascular and kidney outcome trials of finerenone may not be attributable to its vascular effects. Additionally, our findings may be partially explained by the fact that finerenone did not reduce the risk of individual atherosclerotic cardiovascular events, such as myocardial infarction and stroke, in those studies [3–5]. In contrast, the three subgroups (younger, lower HbA1c levels, and higher LVEF) in which finerenone favoured a reduction in CAVI may be responders to the therapy, at least regarding the improvement in vascular stiffness.

Reduction in albuminuria through MR signalling blockade is evident and common in diverse patient populations, including those with T2D and CKD [15–17]. Finerenone also led to a sustained 30% reduction in UACR over 6 months relative to placebo in patients with HF and LVEF ≥ 40% [18], and the magnitude of UACR reduction was similar to that observed in the present study. Nevertheless, we found no association between the changes in CAVI and UACR levels. This emphasises that the clinical benefits of finerenone stem from divergent mechanisms apart from its effect on vascular stiffness. In a mediation analysis using pooled data from the FIDELIO-DKD and FIGARO-DKD trials [19], early UACR reduction accounted for a substantial (84%) proportion of the effect of finerenone therapy on kidney outcomes and a more modest (37%) proportion of treatment effect on cardiovascular outcomes. These findings highlight the mechanistic role of lowering intraglomerular pressure in the clinical benefits of finerenone.

Recent preclinical work by Chen et al. [20, 21] demonstrated that empagliflozin's renoprotective and cardioprotective effects were linked to the activation of the tubuloglomerular feedback mechanism, leading to reduced intraglomerular pressure and blunting of the complement system and cardiomyocyte hypertrophy. The reduction in albuminuria without affecting CAVI, which was observed during finerenone therapy in the present study (where the prevalence of SGLT2 inhibitor use was high at 64.4%), suggests that its primary mechanism is likely renal haemodynamic modulations. Therefore, the combined use of these agents may provide complementary renal haemodynamic protective actions (afferent vasoconstriction via SGLT2 inhibition and efferent vasodilation via finerenone) to optimally and efficiently attenuate intraglomerular hypertension. This supports the findings of a more recent clinical trial showing that simultaneous administration of both drug classes reduced urinary albumin more than either treatment alone in patients with T2D and CKD [22, 23].

Regarding the renal biomarkers tested, we observed an early decline in eGFR following the initiation of finerenone, consistent with observations in recent clinical trials for patients with advanced CKD and HF [3, 18]. In the FIDELIO-DKD trial, after the initial decline in eGFR, eGFR declined at a slower rate in finerenone-treated participants and eGFR trajectories crossed that of placebo-treated participants after 24 months [3]. Although the clinical impact of the early decline in eGFR with finerenone remains unclear, recent evidence suggests that this phenomenon is not associated with worse outcomes in patients with HF and LVEF ≥ 40% [24]. Additionally, the magnitude of the decline did not influence the treatment effect of finerenone. Notably, in the present study, the early eGFR dip was not accompanied by evidence of kidney injury based on the specific biomarkers tested. Moreover, considering a marginal decline in urinary liver fatty acid-binding protein levels after 24 weeks of finerenone therapy (Table 2), the therapy may also attenuate the tubulointerstitial damage and renal ischemic stress, potentially contributing to the improvement of renal outcomes. Thus, these biomarker changes may signal potential kidney injury protection following finerenone therapy, but this requires external validation and further testing. Taken together, these mechanistic data support current clinical guidance recommended in the KDIGO 2024 guidelines [2] that the early eGFR dip expected with finerenone does not denote evidence of kidney injury and should not prompt automatic treatment disruption or discontinuation.

We observed a modest but significant decrease in systolic blood pressure [− 6.1 mm Hg (95%CI − 12.1 to − 0.1)] over 24 weeks in patients treated with finerenone compared with those treated with placebo. Given modest increases in serum potassium, aldosterone concentration, and renin activity, finerenone-induced blood pressure lowering was likely mediated by natriuresis resulting from renal MR antagonism. Additionally, vascular contraction and tone may have been partly favourably affected and modified through SMC-MR blocking [10]. However, the FIDELIO-DKD trial revealed that reductions in systolic blood pressure [− 2.7 mm Hg (95%CI − 3.3 to − 2.1)] explained only a small proportion of the treatment effect of finerenone on cardiorenal outcomes in patients with T2D and CKD [25]. Similarly, in participants with heart failure in the FINEARTS-HF [26], early modest reductions in systolic blood pressure [− 3.4 mm Hg (95%CI − 2.6 to − 4.2)] with finerenone did not account for the long-term cardiovascular benefits of the therapy. The reason for this difference in the blood pressure reductions among trials remains uncertain; the background differences in the patient populations might have contributed at least in part. Moreover, the blood pressure reduction caused by finerenone was generally modest, and simultaneously, such blood pressure reduction is unlikely to drive the large proportion of cardiorenal benefits with finerenone therapy. Hence, these data suggest that non-haemodynamic pathways are closely involved in the clinical benefits of finerenone.

The present proteomic analysis identified, for the first time, a nominal decrease in six proteins and an increase in five proteins with finerenone therapy compared with placebo. Most of the downregulated proteins are associated with the inflammatory response and are predictive and prognostic markers of cardiovascular and kidney diseases [27–34]. Particularly, a downregulation of osteopontin levels was consistent to previous experimental study with deoxycorticosterone acetate-/salt-challenged rats [35]. Experimental studies have also demonstrated that some proteins, such as chitinase-3 like-protein-1, catepsin D, and suppression of tumourigenesis-2, could be potential target proteins for reducing renal fibrosis [36–38]. However, uncertainty remains regarding whether and how the elevated proteins, including those with roles in protease activity, cell survival, and immune response, explain the mode of action of finerenone. In a previous proteomic analysis of spironolactone, a steroidal MR antagonist, for patients at risk of HF [39], spironolactone therapy also increased several proteins from the Olink® Target 96 Cardiovascular II, Cardiovascular III, and Inflammation panels. Nominal increases in tumour necrosis factor receptor superfamily member 9 and cystatin D were consistent with our findings. Sun et al. [40, 41]. reported that glial cell line-derived neurotrophic factor enhances the anti-inflammatory effect of human adipose-tissue-derived mesenchymal stem cells, mitigating renal fibrosis and functional impairment. Thus, proteins detected in the present study and their pathological network may provide further mechanistic insights into the effects of finerenone.

This study has some limitations. First, the effect size (− 0.6 SD) was used for the power calculation, which was extrapolated from an SGLT2 inhibitor study in patients with HF and reduced LVEF [12]. This might raise the possibility that the assumed effect was too large for the present study cohort, potentially leading to an underpowered study for the primary endpoint. Second, this study had a relatively small sample size, and the subgroup analyses were based on very small subgroups within an already small sample. Therefore, these should be framed strictly as exploratory, hypothesis-generating findings that require confirmation in larger datasets to avoid overinterpretation. Third, the clinical characteristics of the FIVE-STAR participants differ somewhat from previous large-scale trials of finerenone (FIDELIO-DKD, FIGARO-DKD) [3, 4]. The present study included older patients with relatively controlled blood pressure and diabetes status, and lower albuminuria. These differences might in part reflect the regional enrolment in FIVE-STAR (restricted to Japanese sites) compared with prior global experiences and better treatment in our contemporary trial (with higher baseline use of SGLT2 inhibitors and glucagon-like peptide-1 receptor agonists). Particularly, the study population exhibited a remarkably high baseline use of SGLT2 inhibitors (64.4%), a class known to improve vascular stiffness in patients with diabetes [42, 43], possibly influencing the null result for the primary CAVI endpoint. It is plausible that the existing vasculoprotective effects of SGLT2i created a "ceiling effect", leaving minimal room for finerenone to demonstrate further improvement in arterial stiffness over the 24-week trial period. Conversely, the administration rate of RAS inhibitors at baseline remained just under 80% in this study cohort, despite these medications being the established standard treatment for patients with CKD concomitant with albuminuria. These factors may have affected the impact of finerenone therapy on study endpoints, and this context is critical for interpreting the generalisability of the study findings. Fourth, the follow-up duration was relatively short, and a longer duration may be necessary for the pharmacological intervention to affect arterial stiffness [44]. Additionally, our findings could provide mechanistic insights into finerenone therapy, but the direct relationship with clinical outcomes could not be assessed due to sample size and study duration for event accrual. Fifth, this study did not establish specific diagnostic criteria for each aetiology of CKD, including diabetic nephropathy, although each attending physician performed differential diagnosis exclusion and definitive diagnosis according to relevant domestic guidelines [45]. This might have led to heterogenous patient population with regards to CKD aetiology. Sixth, among the on-treatment population treated with finerenone at week 24 (n = 47), eight patients (17.0%) did not receive the planned maintenance dose of 20 mg daily. Finally, as the proteomic analysis was an exploratory endpoint, the lack of significance after correcting for multiplicity testing should be noted, and our findings require further validation. Measurements of more proteins beyond the Olink® Target 96 panels examined could further elucidate the pathophysiological and pharmacological pathways of finerenone. Further research is required to better understand potential proteomic signatures having an association with vascular responders to finerenone therapy and its clinical benefits.

In conclusion, in this 24-week mechanistic trial in patients with T2D and CKD, finerenone did not affect changes in vascular stiffness but led to a significant and sustained reduction in albuminuria levels. Our findings suggest that the clinical benefits of finerenone stem from lowering intraglomerular pressure rather than from its effect on vascular stiffness in this population. Additionally, finerenone could modify several cardiovascular and inflammatory biomarkers, potentially highlighting non-haemodynamic pathways of the therapy.

## Supplementary Information

Below is the link to the electronic supplementary material.

Supplementary Material 1

## Data Availability

The data underlying this work are available upon reasonable request from researchers who submit a detailed proposal outlining their intended use and after approval by the principal investigator of the FIVE-STAR study. Inquiries are to be addressed to the corresponding authors.

## Abbreviations

CAVI: Cardio-ankle vascular index
CI: Confidence interval
CKD: Chronic kidney disease
eGFR: Estimated glomerular filtration rate
FAS: Full analysis set
HF: Heart failure
KDIGO: Kidney Disease: Improving Global Outcomes
LVEF: Left ventricular ejection fraction
MR: Mineralocorticoid receptor
NPX: Normalised protein expression
SGLT2: Sodium-glucose co-transporter 2
SMC: Smooth muscle cell
T2D: Type 2 diabetes
UACR: Urinary albumin-to-creatinine ratio

## References

[1] Navaneethan SD, Zoungas S, Caramori ML, Chan JCN, Heerspink HJL, Hurst C, et al. Diabetes management in chronic kidney disease: synopsis of the KDIGO 2022 clinical practice guideline update. Ann Intern Med. 2023;176:381–7. 10.7326/M22-2904. 36623286 10.7326/M22-2904

[2] Kidney Disease: Improving Global Outcomes (KDIGO) CKD Work Group. KDIGO 2024 clinical practice guideline for the evaluation and management of chronic kidney disease. Kidney Int. 2024;105(4):S117–314. 10.1016/j.kint.2023.10.018. 38490803 10.1016/j.kint.2023.10.018

[3] Bakris GL, Agarwal R, Anker SD, Pitt B, Ruilope LM, Rossing P, et al. Effect of finerenone on chronic kidney disease outcomes in type 2 diabetes. N Engl J Med. 2020;383:2219–29. 10.1056/NEJMoa2025845. 33264825 10.1056/NEJMoa2025845

[4] Pitt B, Filippatos G, Agarwal R, Anker SD, Bakris GL, Rossing P, et al. Cardiovascular events with finerenone in kidney disease and type 2 diabetes. N Engl J Med. 2021;385:2252–63. 10.1056/NEJMoa2110956. 34449181 10.1056/NEJMoa2110956

[5] Agarwal R, Filippatos G, Pitt B, Anker SD, Rossing P, Joseph A, et al. Cardiovascular and kidney outcomes with finerenone in patients with type 2 diabetes and chronic kidney disease: the FIDELITY pooled analysis. Eur Heart J. 2022;43:474–84. 10.1093/eurheartj/ehab777. 35023547 10.1093/eurheartj/ehab777 PMC8830527

[6] Barrera-Chimal J, Girerd S, Jaisser F. Mineralocorticoid receptor antagonists and kidney diseases: pathophysiological basis. Kidney Int. 2019;96(2):302–19. 10.1016/j.kint.2019.02.030. 31133455 10.1016/j.kint.2019.02.030

[7] Agarwal R, Kolkhof P, Bakris G, Bauersachs J, Haller H, Wada T, et al. Steroidal and non-steroidal mineralocorticoid receptor antagonists in cardiorenal medicine. Eur Heart J. 2021;42(2):152–61. 10.1093/eurheartj/ehaa736. 33099609 10.1093/eurheartj/ehaa736 PMC7813624

[8] Savarese G, Lindberg F, Filippatos G, Butler J, Anker SD. Mineralocorticoid receptor overactivation: targeting systemic impact with non-steroidal mineralocorticoid receptor antagonists. Diabetologia. 2024;67:246–62. 10.1007/s00125-023-06031-1. 38127122 10.1007/s00125-023-06031-1 PMC10789668

[9] DuPont JJ, Jaffe IZ. 30 years of the mineralocorticoid receptor: the role of the mineralocorticoid receptor in the vasculature. J Endocrinol. 2017;234(1):T67–82. 10.1530/JOE-17-0009. 28634267 10.1530/JOE-17-0009 PMC5518626

[10] Camarda ND, Ibarrola J, Biwer LA, Jaffe IZ. Mineralocorticoid receptors in vascular smooth muscle: blood pressure and beyond. Hypertension. 2024;81:1008–20. 10.1161/HYPERTENSIONAHA.123.21358. 38426347 10.1161/HYPERTENSIONAHA.123.21358 PMC11023801

[11] Tanaka A, Shibata H, Imai T, Yoshida H, Miyazono M, Takahashi N, et al. Rationale and design of an investigator-initiated, multicenter, prospective, placebo-controlled, double-blind, randomized trial to evaluate the effects of finerenone on vascular stiffness and cardiorenal biomarkers in type 2 diabetes and chronic kidney disease (FIVE-STAR). Cardiovasc Diabetol. 2023;22(1):194. 10.1186/s12933-023-01928-y. 37525257 10.1186/s12933-023-01928-y PMC10391880

[12] Requena-Ibáñez JA, Santos-Gallego CG, Rodriguez-Cordero A, Vargas-Delgado AP, Mancini D, Sartori S, et al. Mechanistic insights of empagliflozin in nondiabetic patients with HFrEF: from the EMPA-TROPISM study. JACC Heart Fail. 2021;9:578–89. 10.1016/j.jchf.2021.04.014. 34325888 10.1016/j.jchf.2021.04.014

[13] Gil-Ortega M, Vega-Martín E, Martín-Ramos M, González-Blázquez R, Pulido-Olmo H, Ruiz-Hurtado G, et al. Finerenone reduces intrinsic arterial stiffness in Munich Wistar Frömter rats, a genetic model of chronic kidney disease. Am J Nephrol. 2020;51(4):294–303. 10.1159/000506275. 32088716 10.1159/000506275

[14] Sakima A, Arima H, Matayoshi T, Ishida A, Ohya Y. Effect of Mineralocorticoid receptor blockade on arterial stiffness and endothelial function: a meta-analysis of randomized trials. Hypertension. 2021;77(3):929–37. 10.1161/HYPERTENSIONAHA.120.16397. 33461316 10.1161/HYPERTENSIONAHA.120.16397

[15] Alexandrou ME, Papagianni A, Tsapas A, Loutradis C, Boutou A, Piperidou A, et al. Effects of mineralocorticoid receptor antagonists in proteinuric kidney disease: a systematic review and meta-analysis of randomized controlled trials. J Hypertens. 2019;37(12):2307–24. 10.1097/HJH.0000000000002187. 31688290 10.1097/HJH.0000000000002187

[16] Barrera-Chimal J, Lima-Posada I, Bakris GL, Jaisser F. Mineralocorticoid receptor antagonists in diabetic kidney disease—mechanistic and therapeutic effects. Nat Rev Nephrol. 2022;18:56–70. 10.1038/s41581-021-00490-8. 34675379 10.1038/s41581-021-00490-8

[17] Yuan CY, Gao YC, Lin Y, Liu L, Shen XG, Zou WL, et al. Effects of mineralocorticoid receptor antagonists for chronic kidney disease: a systemic review and meta-analysis. Am J Nephrol. 2024;55:1–17. 10.1159/000534366. 37793348 10.1159/000534366

[18] Mc Causland FR, Vaduganathan M, Claggett BL, Kulac IJ, Desai AS, Jhund PS, et al. Finerenone and kidney outcomes in patients with heart failure: the FINEARTS-HF trial. J Am Coll Cardiol. 2025;85(2):159–68. 10.1016/j.jacc.2024.10.091. 39490700 10.1016/j.jacc.2024.10.091

[19] Agarwal R, Tu W, Farjat AE, Farag YMK, Toto R, Kaul S, et al. Impact of finerenone-induced albuminuria reduction on chronic kidney disease outcomes in type 2 diabetes : a mediation analysis. Ann Intern Med. 2023;176:1606–16. 10.7326/M23-1023. 38048573 10.7326/M23-1023

[20] Chen X, Delić D, Cao Y, Shen L, Shao Q, Zhang Z, et al. Renoprotective effects of empagliflozin are linked to activation of the tubuloglomerular feedback mechanism and blunting of the complement system. Am J Physiol Cell Physiol. 2023;324(4):C951–62. 10.1152/ajpcell.00528.2022. 36779666 10.1152/ajpcell.00528.2022 PMC10085567

[21] Chen X, Wohnhaas CT, Delić D, Schommer N, Duerschmied D, Cao Y, et al. Empagliflozin reduces left ventricular mass increase and improves cardiomyocyte hypertrophy after 5/6 nephrectomy. Biomed Pharmacother. 2025;191:118497. 10.1016/j.biopha.2025.118497. 40882522 10.1016/j.biopha.2025.118497

[22] Agarwal R, Green JB, Heerspink HJL, Mann JFE, McGill JB, Mottl AK, et al. Finerenone with empagliflozin in chronic kidney disease and type 2 diabetes. N Engl J Med. 2025;393:533–43. 10.1056/NEJMoa2410659. 40470996 10.1056/NEJMoa2410659

[23] Agarwal R, Green JB, Heerspink HJL, Mann JFE, McGill JB, Mottl A, et al. Impact of simultaneous initiation of finerenone and empagliflozin on urinary albumin-to-creatinine ratio in Asia: pre-specified analysis of CONFIDENCE. Clin J Am Soc Nephrol. 2025. 10.2215/CJN.0000000865. 40965042 10.2215/CJN.0000000865 PMC13135046

[24] Matsumoto S, Jhund PS, Henderson AD, Bauersachs J, Claggett BL, Desai AS, et al. Initial decline in glomerular filtration rate with finerenone in HFmrEF/HFpEF: a prespecified analysis of FINEARTS-HF. J Am Coll Cardiol. 2025;85(2):173–85. 10.1016/j.jacc.2024.11.020. 39814476 10.1016/j.jacc.2024.11.020

[25] Ruilope LM, Agarwal R, Anker SD, Filippatos G, Pitt B, Rossing P, et al. Blood pressure and cardiorenal outcomes with finerenone in chronic kidney disease in type 2 diabetes. Hypertension. 2022;79:2685–95. 10.1161/HYPERTENSIONAHA.122.19744. 36252131 10.1161/HYPERTENSIONAHA.122.19744 PMC9640256

[26] Solomon SD, McMurray JJV, Vaduganathan M, Claggett B, Jhund PS, Desai AS, et al. Finerenone in heart failure with mildly reduced or preserved ejection fraction. N Engl J Med. 2024;391:1475–85. 10.1056/NEJMoa2407107. 39225278 10.1056/NEJMoa2407107

[27] Patel SM, Lopes MS, Morrow DA, Bellavia A, Bhatt AS, Butler KK, et al. Targeted proteomic profiling of cardiogenic shock in the cardiac intensive care unit. Eur Heart J Acute Cardiovasc Care. 2024;13(8):624–8. 10.1093/ehjacc/zuae068. 38815149 10.1093/ehjacc/zuae068 PMC11350432

[28] Qu Z, Lu Y, Ran Y, Xu D, Guo Z, Cheng M. Chitinase-3 like-protein-1: a potential predictor of cardiovascular disease (Review). Mol Med Rep. 2024;30:176. 10.3892/mmr.2024.13300. 39129301 10.3892/mmr.2024.13300 PMC11332322

[29] Gungor O, Unal HU, Guclu A, Gezer M, Eyileten T, Guzel FB, et al. IL-33 and ST2 levels in chronic kidney disease: associations with inflammation, vascular abnormalities, cardiovascular events, and survival. PLoS ONE. 2017;12(6):e0178939. 10.1371/journal.pone.0178939. 28614418 10.1371/journal.pone.0178939 PMC5470678

[30] Lok ZSY, Lyle AN. Osteopontin in vascular disease. Arterioscler Thromb Vasc Biol. 2019;39(4):613–22. 10.1161/ATVBAHA.118.311577. 30727754 10.1161/ATVBAHA.118.311577 PMC6436981

[31] Núñez J, de la Espriella R, Rossignol P, Voors AA, Mullens W, Metra M, et al. Congestion in heart failure: a circulating biomarker‐based perspective. A review from the Biomarkers Working Group of the Heart Failure Association, European Society of Cardiology. Eur J Heart Fail. 2022;24:1751–66. 10.1002/ejhf.2664. 36039656 10.1002/ejhf.2664

[32] Maddaloni E, Coraggio L, Amendolara R, Baroni MG, Cavallo MG, Copetti M, et al. Association of osteocalcin, osteoprotegerin, and osteopontin with cardiovascular disease and retinopathy in type 2 diabetes. Diabetes Metab Res Rev. 2023;39(5):e3632. 10.1002/dmrr.3632. 36880127 10.1002/dmrr.3632

[33] Steinbrenner I, Sekula P, Kotsis F, von Cube M, Cheng Y, Nadal J, et al. Association of osteopontin with kidney function and kidney failure in chronic kidney disease patients: the GCKD study. Nephrol Dial Transplant. 2023;38(6):1430–8. 10.1093/ndt/gfac173. 35524694 10.1093/ndt/gfac173

[34] Fu Y, Song C, Qin Y, Zheng T, Zhou X, Zhao X, et al. Clinical value of serum MMP-3 in chronic kidney disease. Clin Chim Acta. 2024;553:117725. 10.1016/j.cca.2023.117725. 38128817 10.1016/j.cca.2023.117725

[35] Kolkhof P, Delbeck M, Kretschmer A, Steinke W, Hartmann E, Bärfacker L, et al. Finerenone, a novel selective nonsteroidal mineralocorticoid receptor antagonist protects from rat cardiorenal injury. J Cardiovasc Pharmacol. 2014;64(1):69–78. 10.1097/FJC.0000000000000091. 24621652 10.1097/FJC.0000000000000091

[36] Montgomery TA, Xu L, Mason S, Chinnadurai A, Lee CG, Elias JA, et al. Breast regression protein–39/chitinase 3–like 1 promotes renal fibrosis after kidney injury via activation of myofibroblasts. J Am Soc Nephrol. 2017;28(11):3218–26. 10.1681/ASN.2017010110. 28679671 10.1681/ASN.2017010110 PMC5661290

[37] Fox C, Cocchiaro P, Oakley F, Howarth R, Callaghan K, Leslie J, et al. Inhibition of lysosomal protease cathepsin D reduces renal fibrosis in murine chronic kidney disease. Sci Rep. 2016;6:20101. 10.1038/srep20101. 26831567 10.1038/srep20101 PMC4735715

[38] Kim YC, Kim KH, Lee S, Jo JW, Park JY, Park MS, et al. ST2 blockade mitigates peritoneal fibrosis induced by TGF-β and high glucose. J Cell Mol Med. 2019;23(10):6872–84. 10.1111/jcmm.14571. 31397957 10.1111/jcmm.14571 PMC6787438

[39] Ferreira JP, Verdonschot J, Wang P, Pizard A, Collier T, Ahmed FZ, et al. Proteomic and mechanistic analysis of spironolactone in patients at risk for HF. JACC: Heart Failure. 2021;9(4):268–77. 10.1016/j.jchf.2020.11.010. 33549556 10.1016/j.jchf.2020.11.010

[40] Wang Z, Li S, Wang Y, Zhang X, Chen L, Sun D. GDNF enhances the anti-inflammatory effect of human adipose-derived mesenchymal stem cell-based therapy in renal interstitial fibrosis. Stem Cell Res. 2019;41:101605. 10.1016/j.scr.2019.101605. 31706095 10.1016/j.scr.2019.101605

[41] Li S, Wang Y, Wang Z, Chen L, Zuo B, Liu C, et al. Enhanced renoprotective effect of GDNF-modified adipose-derived mesenchymal stem cells on renal interstitial fibrosis. Stem Cell Res Ther. 2021;12:27. 10.1186/s13287-020-02049-z. 33413640 10.1186/s13287-020-02049-z PMC7792009

[42] Patoulias D, Papadopoulos C, Kassimis G, Fragakis N, Vassilikos V, Karagiannis A, et al. Effect of sodium-glucose co-transporter-2 inhibitors on arterial stiffness: a systematic review and meta-analysis of randomized controlled trials. Vasc Med. 2022;27:433–9. 10.1177/1358863X221101653. 35754338 10.1177/1358863X221101653

[43] Sridharan K, Sivaramakrishnan G. Sodium glucose cotransporter-2 inhibitors improve endothelial function and arterial stiffness in diabetic individuals: a systematic review and network meta-analysis. Curr Vasc Pharmacol. 2025;23(4):272–80. 10.2174/0115701611337138241226101956. 39779556 10.2174/0115701611337138241226101956

[44] Kinouchi K, Ichihara A, Sakoda M, Kurauchi-Mito A, Murohashi-Bokuda K, Itoh H. Effects of telmisartan on arterial stiffness assessed by the cardio-ankle vascular index in hypertensive patients. Kidney Blood Press Res. 2010;33:304–12. 10.1159/000316724. 20664284 10.1159/000316724

[45] Japanese Society of Nephrology. Essential points from evidence-based clinical practice guideline for chronic kidney disease 2023. Clin Exp Nephrol. 2024;28:473–95. 10.1007/s10157-024-02497-4. 38713253 10.1007/s10157-024-02497-4 PMC11116248

