# Supplementary material for: Effects of finerenone on arterial stiffness and cardiorenal biomarkers in patients with type 2 diabetes and chronic kidney disease: a randomised placebo-controlled mechanistic trial (FIVE-STAR)
Source: Cardiovasc Diabetol. 2025 Dec 5;24:454. doi: 10.1186/s12933-025-03014-x (PMC12681115; doi:10.1186/s12933-025-03014-x)
Supplement: Supplementary file 1 — Supplementary Material 1 [file 12933_2025_3014_MOESM1_ESM.docx]

**Supplementary Appendix**

**Effects of finerenone on arterial stiffness and cardiorenal biomarkers in patients with type 2 diabetes and chronic kidney disease: a randomised placebo-controlled mechanistic trial (FIVE-STAR)**

Atsushi Tanaka, MD, PhD,^1^ Muthiah Vaduganathan, MD, MPH,^2^ Takumi Imai, PhD,^3,4^ Yosuke Okada, MD, PhD,^5^ Satomi Sonoda, MD, PhD,^6^ Keiichi Torimoto, MD, PhD,^6^ Satoru Suwa, MD,^7^ Hiroki Teragawa, MD, PhD,^8^ Motoaki Miyazono, MD, PhD,^9^ Makoto Fukuda, MD,^9^ Keisuke Yonezu, MD, PhD,^10^ Naohiko Takahashi, MD, PhD,^10^ Yuichi Yoshida, MD, PhD,^11^ Kenichi Tanaka, MD, PhD,^12^ Michio Shimabukuro, MD, PhD,^13^ Yuki Hotta, MD,^13^ Masao Moroi, MD, PhD,^14^ Hiroki Niikura, MD,^14^ Keisuke Kida, MD, PhD,^15^ Kenichi Yokota, MD, PhD,^16^ Daiju Fukuda, MD, PhD,^17^ Kengo Tanabe, MD, PhD,^18^ Yu Horiuchi, MD, PhD,^18^ Shigeru Toyoda, MD, PhD,^19^ Isao Taguchi, MD, PhD,^20^ Hisako Yoshida, PhD,^21^ Toru Miyoshi, MD, PhD,^22^ Masaomi Nangaku, MD, PhD,^23^ Hirotaka Shibata, MD, PhD,^11^ Koichi Node, MD, PhD,^1^ on behalf of the FIVE-STAR Investigators.

^1^ Department of Cardiovascular Medicine, Saga University, Saga, Japan

^2^Division of Cardiovascular Medicine, Brigham and Women's Hospital, Harvard Medical School, Boston, MA, USA

^3^Clinical Research Division, Organization for Clinical Medicine Promotion, Tokyo, Japan

^4^ Clinical and Translational Research Center, Kobe University Hospital, Kobe, Japan

^5^ Clinical Research Center, Hospital of the University of Occupational and Environmental Health, Japan, Kitakyushu, Japan

^6^ First Department of Internal Medicine, University of Occupational and Environmental Health, Japan, Kitakyushu, Japan

^7^Department of Cardiology, Juntendo University Shizuoka Hospital, Izunokuni, Japan

^8^Department of Cardiovascular Medicine, JR Hiroshima Hospital, Hiroshima, Japan

^9^Department of Nephrology, Saga University, Saga, Japan

^10^Department of Cardiology and Clinical Examination, Faculty of Medicine, Oita University, Yufu, Japan

^11^Department of Endocrinology, Metabolism, Rheumatology and Nephrology, Faculty of Medicine, Oita University, Yufu, Japan

^12^Wakamatsu Hospital of the University of Occupational and Environmental Health, Japan, Kitakyushu, Japan

^13^Department of Diabetes, Endocrinology, and Metabolism Fukushima Medical University School of Medicine, Fukushima, Japan

^14^Division of Cardiovascular Medicine, Toho University Ohashi Medical Center, Tokyo, Japan

^15^Department of Pharmacology, St. Marianna University School of Medicine, Kawasaki, Japan

^16^Division of Metabolism and Endocrinology, Department of Internal Medicine, St. Marianna University School of Medicine, Kawasaki, Japan

^17^ Department of Cardiovascular Medicine, Osaka Metropolitan University Graduate School of Medicine, Osaka, Japan

^18^Division of Cardiology, Mitsui Memorial Hospital, Tokyo, Japan

^19^Department of Cardiovascular Medicine, Dokkyo Medical University, Mibu, Japan

^20^Department of Cardiology, Dokkyo Medical University Saitama Medical Center, Koshigaya, Japan

^21^Department of Medical Statistics, Osaka Metropolitan University Graduate School of Medicine, Osaka, Japan

^22^Department of Cardiovascular Medicine, Okayama University Graduate School of Medicine, Dentistry and Pharmaceutical Sciences, Okayama, Japan

^23^Division of Nephrology and Endocrinology, The University of Tokyo Graduate School of Medicine, Tokyo, Japan

**Address for correspondence:** Dr. Atsushi Tanaka and Dr. Koichi Node, Department of Cardiovascular Medicine, Saga University, 5-1-1 Nabeshima, Saga 849-8501, Japan.

TEL: +81-952-34-2364

FAX: +81-952-34-2089

Email: tanakaa2@cc.saga-u.ac.jp (Dr. Tanaka) and node@cc.saga-u.ac.jp (Dr. Node)

**Table of contents**

[P4. Supplementary Text S1. Study Protocol](#Supplementary1)

[P81. Supplementary Text S2. Study sites list](#Supplementary2)

[P82. Supplementary Text S3. Statistical Analysis Plan](#Supplementary3)

[P99. Supplementary Figure S1. Background KDIGO risk categories of patients included in FIVE-STAR trial](#Supplementary4)

[P100. Supplementary Figure S2. CAVI response in prespecified subgroups](#Supplementary5)

[P102. Supplementary Figure S3. Changes in CAVI in a subgroup (age <70 years)](#Supplementary6)

[P103. Supplementary Figure S4. Changes in CAVI in a subgroup (HbA1c <6.8%)](#Supplementary7)

[P104. Supplementary Figure S5. Changes in CAVI in a subgroup (LVEF ≥60%)](#Supplementary8)

[P105. Supplementary Figure S6. Correlation between changes in CAVI and UACR](#Supplementary9)

[P106. Supplementary Figure S7. Focused network of proteins influenced by finerenone with a nominal P value of <0.05.](#Supplementary10)

[P107. Supplementary Table S1. Doses of study drugs in on-treatment patients at week 24](#Supplementary11)

[P108. Supplementary Table S2. Changes in clinical and laboratory measures](#Supplementary12)

[P114. Supplementary Table S3. Effect on circulating proteins from Cardiovascular III and Inflammation panels](#Supplementary13)

[P116. Supplementary Table S4. Safety endpoints](#Supplementary14)

| **Supplementary Text S1** |
| --- |
| Effects of FInerenone on Vascular stiffnEss and cardiorenal biomarkerS  in Type 2 diAbetes and chRonic kidney disease  (FIVE-STAR) |
| Study Protocol |
| Chief Principal Investigator: Koichi Node  Affiliation: Department of Cardiology, Saga University Hospital  Address: 5-1-1 Nabeshima, Saga-shi, Saga, 849-8501  Contact: TEL 0952-34-2364 |
| Creation date: April 12, 2023 |
| Version: 1.1 |

Confidentiality

The information included in this research plan must not be disclosed to third parties except those directly involved in this research, members of the certified review board and the review organization of the medical institution conducting this research, and officials of the authorities, including the Minister of Health, Labour and Welfare. Furthermore, the information in this research plan must not be used for purposes other than the implementation and evaluation of this research without the prior written consent of the chief principal investigator.

Revision history

| Version | Creation and revision dates | Reasons for revision | Scheduled implementation date |
| --- | --- | --- | --- |
| Version 1.0  (Draft Edition) | January 27, 2023 | Newly created | - |
| Version 1.1 | April 12, 2023 | For approval by the Certified Review Board | 2023/5/1 |

Definitions of abbreviations

| Abbreviation | Definition (English) |
| --- | --- |
| ACE | Angiotensin-converting enzyme |
| AI | Augmentation index |
| ARB | Angiotensin II receptor blocker |
| ARNI | Angiotensin receptor-neprilysin inhibitors |
| BMI | Body mass index |
| CAVI | Cardio-ankle vascular index |
| CKD | Chronic kidney disease |
| CRB | Certified review board |
| CRF | Case report form |
| DPP-4 | Dipeptidyl peptidase 4 |
| EDC | Electric data capture |
| eEV | Estimated extracellular volume |
| eGFR | Estimated glomerular filtration rate |
| ESA | Erythropoiesis stimulating agent |
| FAS | Full analysis set |
| GLP-1 | Glucagon-like peptide-1 |
| HbA1c | Hemoglobin A1c |
| HFrEF | Heart failure with reduced ejection fraction |
| HIF-PH | Hypoxia-inducible factor prolyl-hydroxylase inhibitor |
| IgA | Immunoglobulin A |
| jRCT | Japan Registry of Clinical Trials |
| LAD | Left atrial dimension |
| LAVI | Left atrial volume index |
| L-FABP | Liver-type fatty acid-binding protein |
| LLT | Lowest level term |
| LVEF | Left ventricular ejection fraction |
| LVMI | Left ventricular mass index |
| MedDRA/J | Medical Dictionary for Regulatory Activities/Japanese version |
| MMRM | Mixed-effects models for repeated measures |
| MRA | Mineralocorticoid receptor antagonist |
| NAG | N-acetyl-β-d-glucosaminidase |
| NGAL | Neutrophil gelatinase-associated lipocalin |
| NYHA | New York Heart Association |
| PPS | Per-protocol set |
| PT | Preferred term |
| RA | Renin-angiotensin |
| SGLT2 | Sodium glucose cotransporter 2 |
| SOC | System organ class |
| SS | Safety set |
| TEAE | Treatment-emergent adverse event |
| UACR | Urine albumin-to-creatinine ratio |
| α1-MG | α1-microglobulin |
| β2-MG | β2-microglobulin |

# **Research summary**

| Research summary | |
| --- | --- |
| Research title | Effects of Finerenone on Vascular Stiffness and Cardiorenal Biomarkers in Chronic Kidney Disease and Type 2 Diabetes |
| Abbreviation | FIVE-STAR |
| Research objectives | To evaluate the effects of finerenone on vascular stiffness using cardio-ankle vascular index (CAVI) and cardiorenal biomarkers in patients with chronic kidney disease and type 2 diabetes. |
| Endpoints | Primary endpoint:  Change in CAVI at 24 weeks after protocol treatment initiation compared to that at baseline  Secondary endpoints:   1. Geometric mean percent change in urine albumin-to-creatinine ratio (UACR) at 12 and 24 weeks after protocol treatment initiation compared to that at baseline (ranked secondary endpoint) 2. Geometric mean percent change in blood biomarkers (pentosidine) and urinary biomarkers including type IV collagen, α1-microglobulin (α1-MG), β2-microglobulin (β2-MG), neutrophil gelatinase-associated lipocalin (NGAL), N-acetyl-β-d-glucosaminidase (NAG), and liver-type fatty acid-binding protein (L-FABP)/ creatinine ratio at 24 weeks after protocol treatment initiation compared to that at baseline   Other endpoints   1. Changes from baseline in vital signs including weight, body mass index (BMI), estimated extracellular volume (eEV), blood pressure, pulse pressure, and pulse rate in the clinic, and blood pressure, pulse pressure, and pulse rate at home at 4, 12, and 24 weeks after protocol treatment initiation 2. Changes from baseline in blood test indices including serum creatinine, estimated glomerular filtration rate (eGFR), serum cystatin C*, serum potassium, hemoglobin A1c (HbA1c)*, plasma aldosterone concentration**, and plasma renin activity** at 4, 12, and 24 weeks after protocol treatment initiation (*Evaluated only at baseline, and 12 and 24 weeks, **Evaluated only at baseline and 24 weeks) 3. Changes from baseline in augmentation index (AI) and % mean arterial pressure at 24 weeks after protocol treatment initiation 4. Changes from baseline in cardiac function indices including left ventricular ejection fraction (LVEF), septal e′, lateral e′, E, E/e′*, left ventricular mass index (LVMI), left atrial dimension (LAD), and left atrial volume index (LAVI) assessed by echocardiography 24 weeks after protocol treatment initiation 5. Changes in abundance of up to 181 proteins comprehensively analyzed by proteomic analysis (Target 96 panel) at 24 weeks after protocol treatment initiation compared to that at baseline (exploratory endpoint)   Safety endpoints:  Adverse events occurring after protocol treatment initiation including hyperkalemia |
| Study design | Investigator-initiated, multicenter, prospective, parallel-group, placebo-controlled, double-blind, randomized controlled trial |
| Study drug | Finerenone (brand name: Kerendia® tablets 10 mg/20 mg) |
| Study participants | Patients with chronic kidney disease and type 2 diabetes |
| Inclusion and exclusion criteria | Inclusion criteria  Patients who meet all of the following criteria are eligible:   1. Patients providing written informed consent to participate in this study 2. Patients aged ≥ 20 years at the time of providing consent (regardless of sex) 3. Patients with type 2 diabetes 4. Patients with chronic kidney disease meeting both the following criteria: 5. eGFR, 25–90 mL/min/1.73 m^2^ 6. UACR, 30–3500 mg/g.cr. 7. Patients who did not change their medication for type 2 diabetes or chronic kidney disease in the 4 weeks before providing consent     Exclusion criteria:  Patients meeting the following criteria will be excluded from this study:   1. Patients currently treated with a mineralocorticoid receptor antagonist (MRA) including finerenone, or those administered MRA in the 4 weeks before providing informed consent 2. Patients with a history of hypersensitivity to finerenone 3. Patients with HbA1c ≥ 10% 4. Patients with serum potassium levels ≥ 4.9 mEq/L 5. Patients with New York Heart Association (NYHA) class II to IV, LVEF ≤ 35%) 6. Patients with poorly controlled hypertension, including those with systolic blood pressure ≥ 170 mmHg, diastolic blood pressure ≥ 110 mmHg, and hypertensive emergency. 7. Patients with a history of ischemic stroke, acute coronary syndrome, cardiovascular surgery or percutaneous treatment, or hospitalization due to worsening heart or renal failure within the 8 weeks before providing consent 8. Patients scheduled to undergo coronary revascularization or other surgical or percutaneous treatment for cardiovascular disease during the observation period 9. Patients scheduled to undergo treatments including electrical defibrillation, cardiac resynchronization therapy, or pacemaker implantation during the observation period 10. Patients scheduled to undergo dialysis or kidney transplantation during the observation period 11. Patients with severe liver dysfunction (Child-Pugh class C) 12. Patients administered itraconazole, ritonavir-containing preparations, atazanavir, darunavir, fosamprenavir, cobicistat-containing preparations, or clarithromycin 13. Patients with Addison’s disease 14. Patients with active infectious disease 15. Patients with pregnancy, possibly pregnancy, or breastfeeding 16. Patients deemed unsuitable for this study by the principal investigator or co-investigator including those with renal artery stenosis, one kidney, and active malignant tumor. |
| Target number of cases | 100 cases (Finerenone group, 50 cases; placebo group, 50 cases) |
| Research period | Case registration period: Japan Registry of Clinical Trials (jRCT) release date  to July 31, 2024  Observation period: 24 weeks  Research implementation period: Japan Registry of Clinical Trials (jRCT) release date to July 31, 2026 |
| Research methodology | After verifying the eligibility of patients who have provided written consent, patients who meet all inclusion criteria will be enrolled in the study. Patients will be randomized (allocated) into the finerenone or placebo groups. They will undergo double-blind administration and observation at baseline (before the initiation of the study drug administration) and weeks 4, 12, and 24, as well as prescribed tests. In principle, baseline tests will be conducted within 60 days of obtaining consent, and the study drug administration will be initiated the following day. |

# **Research background and objectives**

## Research background

Type 2 diabetes is one of the major causes of chronic kidney disease (CKD), with the combination of the two conditions being a high-risk state for end-stage renal failure and cardiovascular disease; therefore, measures for prevention of progression of these conditions are important (1, 2). Conventionally, in addition to dietary and glucose-lowering therapies, antihypertensive therapy with renin-angiotensin (RA) system inhibitors, including angiotensin-converting enzyme (ACE) inhibitors and angiotensin II receptor blockers (ARBs), has been recommended for patients with hypertension to prevent nephropathy progression. In recent years, sodium glucose cotransporter 2 (SGLT2) inhibitors have been reported to suppress cardiovascular disease risk and nephropathy progression in patients with CKD regardless of the presence or absence of type 2 diabetes (3, 4), and are expected to be a new therapeutic agent for CKD (5). However, diabetes is the most significant factor associated with the need for introduction of maintenance dialysis in Japan, and sufficient drug therapy for patients with CKD and type 2 diabetes has not been established yet. Therefore, developing a treatment module to reduce the potential residual risk of nephropathy progression in patients with CKD and diabetes is necessary along with verification of its efficacy and safety in clinical practice.

Excessive mineralocorticoid receptor activation at systemic and local levels induces inflammation and fibrosis, and this plays a significant role in organ and tissue injury in the cardiovascular and renal systems (6-9), making them an important therapeutic target. Specifically, mineralocorticoid receptor antagonists (MRAs) are widely used in clinical practice as therapeutic agents for patients with hypertension and heart failure with reduced ejection fraction (HFrEF) (10,11). Moreover, previous clinical trials have reported that MRAs reduce proteinuria (albuminuria) in patients with CKD (12-14). However, evidence on the effects of MRAs on clinical outcomes and their therapeutic potential in patients with CKD and diabetes (15) is limited.

Finerenone is a novel nonsteroidal selective MRA with higher selectivity and affinity for mineralocorticoid receptors than that of conventional steroidal MRAs, and has even distribution in cardiac and renal tissues (16,17). The international joint phase III trials using finerenone reported in succession since 2020 (FIDELIO-DKD, FIGARO-DKD) revealed that finerenone inhibited nephropathy progression and reduced cardiovascular event risks in patients with CKD and type 2 diabetes who were administered with standard treatment including RA system inhibitors (18,19). Therefore, finerenone was approved in Japan in March 2022 as a treatment for patients with CKD and type 2 diabetes.

Based on its pharmacological action and the mechanism of the clinical benefits, finerenone likely exhibits systemic and local anti-inflammatory and anti-fibrotic effects via mineralocorticoid receptors. However, its effects on specific cardiac and renal clinical indicators have not been completely understood in clinical practice. Moreover, various studies have reported that increased vascular stiffness is closely associated with CKD and nephropathy progression and cardiovascular disease risk in various populations, including patients with diabetes (20-23). Furthermore, MRA administration has been reported to improve vascular function, including vascular stiffness (24); thus, one of the mechanisms of finerenone’s clinical benefits may be based on cardiovascular and renal protective effects via the improvement of systemic and local vascular stiffness; however, the detailed mechanism remains unclear. The CAVI, the primary endpoint in this study, has been reported to have a positive correlation with the risk of developing cardiovascular events and worsening renal function (25-31). Thus, characterization of the effect of finerenone on CAVI, a surrogate marker for cardiorenal vascular events, as well as comprehensive and multifaceted evaluation of the impact of finerenone on various other surrogate biomarkers for cardiac and renal diseases will lead to detailed elucidation of the mechanism of action of finerenone and the establishment of clinical evidence for the drug in Japan.

## References:

1. Li H, Lu W, Wang A, Jiang H, Lyu J. Changing epidemiology of chronic kidney disease as a result of type 2 diabetes mellitus from 1990 to 2017: Estimates from Global Burden of Disease 2017. J Diabetes Investig. 2021 Mar;12(3):346–356.
2. GBD Chronic Kidney Disease Collaboration. Global, regional, and national burden of chronic kidney disease, 1990-2017: a systematic analysis for the Global Burden of Disease Study 2017. Lancet. 2020 Feb 29;395(10225):709–733.
3. Perkovic V, Jardine MJ, Neal B, Bompoint S, Heerspink HJL, Charytan DM, Edwards R, Agarwal R, Bakris G, Bull S, Cannon CP, Capuano G, Chu PL, de Zeeuw D, Greene T, Levin A, Pollock C, Wheeler DC, Yavin Y, Zhang H, Zinman B, Meininger G, Brenner BM, Mahaffey KW; CREDENCE Trial Investigators. Canagliflozin and renal outcomes in type 2 diabetes and nephropathy. N Engl J Med. 2019 Jun 13;380(24):2295–2306.
4. Heerspink HJL, Stefánsson BV, Correa-Rotter R, Chertow GM, Greene T, Hou FF, Mann JFE, McMurray JJV, Lindberg M, Rossing P, Sjöström CD, Toto RD, Langkilde AM, Wheeler DC; DAPA-CKD Trial Committees and Investigators. Dapagliflozin in patients with chronic kidney disease. N Engl J Med. 2020 Oct 8;383(15):1436–1446.
5. American Diabetes Association Professional Practice Committee, Draznin B, Aroda VR, et al. 9. Pharmacologic approaches to glycemic treatment: standards of medical care in diabetes-2022. Diabetes Care. 2022;45(Suppl 1):S125–S143.
6. Shibata H, Itoh H. Mineralocorticoid receptor-associated hypertension and its organ damage: clinical relevance for resistant hypertension. Am J Hypertens. 2012;25(5):514–523.
7. Buonafine M, Bonnard B, Jaisser F. Mineralocorticoid receptor and cardiovascular disease. Am J Hypertens. 2018 Oct 15;31(11):1165–1174.
8. Jia G, Aroor AR, Sowers JR. The role of mineralocorticoid receptor signaling in the cross-talk between adipose tissue and the vascular wall. Cardiovasc Res. 2017 Jul 1;113(9):1055–1063.
9. Barrera-Chimal J, Girerd S, Jaisser F. Mineralocorticoid receptor antagonists and kidney diseases: pathophysiological basis. Kidney Int. 2019 Aug;96(2):302–319.
10. Bauersachs J. Heart failure drug treatment: the fantastic four. Eur Heart J. 2021 Feb 11;42(6):681–683.
11. Tsutsui H, Ide T, Ito H, Kihara Y, Kinugawa K, Kinugawa S, Makaya M, Murohara T, Node K, Saito Y, Sakata Y, Shimizu W, Yamamoto K, Bando Y, Iwasaki YK, Kinugasa Y, Mizote I, Nakagawa H, Oishi S, Okada A, Tanaka A, Akasaka T, Ono M, Kimura T, Kosaka S, Kosuge M, Momomura SI. JCS/JHFS 2021 Guideline focused update on diagnosis and treatment of acute and chronic heart failure. J Card Fail. 2021 Dec;27(12):1404–1444.
12. Ando K, Ohtsu H, Uchida S, Kaname S, Arakawa Y, Fujita T; EVALUATE Study Group. Anti-albuminuric effect of the aldosterone blocker eplerenone in non-diabetic hypertensive patients with albuminuria: a double-blind, randomized, placebo-controlled trial. Lancet Diabetes Endocrinol. 2014 Dec;2(12):944–953.
13. Bakris GL, Agarwal R, Chan JC, Cooper ME, Gansevoort RT, Haller H, Remuzzi G, Rossing P, Schmieder RE, Nowack C, Kolkhof P, Joseph A, Pieper A, Kimmeskamp-Kirschbaum N, Ruilope LM. Mineralocorticoid Receptor Antagonist Tolerability Study–Diabetic Nephropathy (ARTS-DN) Study Group. Effect of finerenone on albuminuria in patients with diabetic nephropathy: A Randomized Clinical Trial. JAMA. 2015 Sep 1;314(9):884–894.
14. Ito S, Kashihara N, Shikata K, Nangaku M, Wada T, Okuda Y, Sawanobori T. Esaxerenone (CS-3150) in patients with type 2 diabetes and microalbuminuria (ESAX-DN): phase 3 randomized controlled clinical trial. Clin J Am Soc Nephrol. 2020 Dec 7;15(12):1715–1727.
15. Blankenburg M, Kovesdy CP, Fett AK, Griner RG, Gay A. Disease characteristics and outcomes in patients with chronic kidney disease and type 2 diabetes: a matched cohort study of spironolactone users and non-users. BMC Nephrol. 2020 Feb 26;21(1):61.
16. Bärfacker L, Kuhl A, Hillisch A, Grosser R, Figueroa-Pérez S, Heckroth H, Nitsche A, Ergüden JK, Gielen-Haertwig H, Schlemmer KH, Mittendorf J, Paulsen H, Platzek J, Kolkhof P. Discovery of BAY 94-8862: a nonsteroidal antagonist of the mineralocorticoid receptor for the treatment of cardiorenal diseases. ChemMedChem. 2012 Aug;7(8):1385–1403.
17. Kolkhof P, Delbeck M, Kretschmer A, Steinke W, Hartmann E, Bärfacker L, Eitner F, Albrecht-Küpper B, Schäfer S. Finerenone, a novel selective nonsteroidal mineralocorticoid receptor antagonist protects from rat cardiorenal injury. J Cardiovasc Pharmacol. 2014 Jul;64(1):69–78.
18. Bakris GL, Agarwal R, Anker SD, Pitt B, Ruilope LM, Rossing P, Kolkhof P, Nowack C, Schloemer P, Joseph A, Filippatos G; FIDELIO-DKD Investigators. Effect of finerenone on chronic kidney disease outcomes in type 2 diabetes. N Engl J Med. 2020 Dec 3;383(23):2219–2229.
19. Pitt B, Filippatos G, Agarwal R, Anker SD, Bakris GL, Rossing P, Joseph A, Kolkhof P, Nowack C, Schloemer P, Ruilope LM; FIGARO-DKD Investigators. Cardiovascular events with finerenone in kidney disease and type 2 diabetes. N Engl J Med. 2021 Dec 9;385(24):2252–2263.
20. Pierce GL. Mechanisms and subclinical consequences of aortic stiffness. Hypertension. 2017 Nov;70(5):848–853.
21. Kim KJ, Lee BW, Kim HM, Shin JY, Kang ES, Cha BS, Lee EJ, Lim SK, Lee HC. Associations between cardio-ankle vascular index and microvascular complications in type 2 diabetes mellitus patients. J Atheroscler Thromb. 2011;18(4):328–336.
22. Miyoshi T, Ito H, Shirai K, Horinaka S, Higaki J, Yamamura S, Saiki A, Takahashi M, Masaki M, Okura T, Kotani K, Kubozono T, Yoshioka R, Kihara H, Hasegawa K, Satoh-Asahara N, Orimo H; CAVI‐J (Prospective Multicenter Study to Evaluate Usefulness of Cardio-Ankle Vascular Index in Japan) investigators *. Predictive value of the cardio-ankle vascular index for cardiovascular events in patients at cardiovascular risk. J Am Heart Assoc. 2021 Aug 17;10(16):e020103.
23. Itano S, Yano Y, Nagasu H, Tomiyama H, Kanegae H, Makino H, Higashi Y, Kobayashi Y, Sogawa Y, Satoh M, Suzuki K, Townsend RR, Budoff M, Bakris G, Kashihara N. Association of arterial stiffness with kidney function among adults without chronic kidney disease. Am J Hypertens. 2020 Nov 3;33(11):1003–1010.
24. Kishimoto S, Oki K, Maruhashi T, Kajikawa M, Matsui S, Hashimoto H, Takaeko Y, Kihara Y, Chayama K, Goto C, Aibara Y, Yusoff FM, Nakashima A, Noma K, Liao JK, Higashi Y. Eplerenone improves endothelial function and arterial stiffness and inhibits Rho-associated kinase activity in patients with idiopathic hyperaldosteronism: a pilot study. J Hypertens. 2019 May;37(5):1083–1095.
25. Chung SL, Yang CC, Chen CC, Hsu YC, Lei MH. Coronary artery calcium score compared with cardio-ankle vascular index in the prediction of cardiovascular events in asymptomatic patients with type 2 diabetes. J Atheroscler Thromb. 2015;22:1255–1265.
26. Hitsumoto T. Clinical significance of cardio-ankle vascular index as a cardiovascular risk factor in elderly patients with type 2 diabetes mellitus. J Clin Med Res. 2018 Apr;10(4):330–336.
27. Zhang C, Zhong Y, Tian H. Increased cardio-ankle vascular index is independently associated with chronic kidney disease: A cross-sectional study in Chinese patients with type 2 diabetes mellitus. J Diabetes Complications. 2019 Sep;33(9):623–627.
28. Saigusa T, Watanabe K, Hada Y, Ishii K, Kameda W, Susa S, Ishizawa K, Ishihara H. Cardio-ankle vascular index is more closely associated than brachial-ankle pulse wave velocity with arterial damage and risk of cardiovascular disease in patients with diabetes. BMC Cardiovasc Disord. 2022 Aug 9;22(1):365.
29. Nakamura K, Iizuka T, Takahashi M, Shimizu K, Mikamo H, Nakagami T, Suzuki M, Hirano K, Sugiyama Y, Tomaru T, Miyashita Y, Shirai K, Noike H. Association between cardio-ankle vascular index and serum cystatin C levels in patients with cardiovascular risk factor. J Atheroscler Thromb. 2009 Aug;16(4):371–379.
30. Kubozono T, Miyata M, Ueyama K, et al. Association between arterial stiffness and estimated glomerular filtration rate in the Japanese general population. J Atheroscler Thromb. 2009;16(6):840-845.
31. Namikoshi T, Fujimoto S, Yorimitsu D, Ihoriya C, Fujimoto Y, Komai N, Sasaki T, Kashihara N. Relationship between vascular function indexes, renal arteriolosclerosis, and renal clinical outcomes in chronic kidney disease. Nephrology (Carlton). 2015 Sep;20(9):585–590.

## Research objectives

To evaluate the effects of finerenone on vascular stiffness using CAVI and cardiorenal biomarkers in patients with CKD and type 2 diabetes.

# **Research contents**

## Endpoints

1) Primary endpoint:

Change in CAVI at 24 weeks after protocol treatment initiation compared to that at baseline

Rationale:

CAVI is widely used in clinical settings at major medical institutions, including university hospitals and health examination facilities, as an index of vascular function (mainly arteriosclerosis and arterial stiffness) not affected by blood pressure. Various studies have reported the association of CAVI with the onset of cardiovascular events and cardiac and renal function in patients with diabetes and CKD (1-7). Furthermore, CAVI is often used as a surrogate marker in clinical trial evaluation items (8-13).

References:

1. Chung SL, Yang CC, Chen CC, Hsu YC, Lei MH. Coronary artery calcium score compared with cardio-ankle vascular index in predicting cardiovascular events in asymptomatic patients with type 2 diabetes. J Atheroscler Thromb. 2015;22:1255–1265.
2. Hitsumoto T. Clinical significance of cardio-ankle vascular index as a cardiovascular risk factor in elderly patients with type 2 diabetes mellitus. J Clin Med Res. 2018 Apr;10(4):330–336.
3. Zhang C, Zhong Y, Tian H. Increased cardio-ankle vascular index is independently associated with chronic kidney disease: A cross-sectional study in Chinese patients with type 2 diabetes mellitus. J Diabetes Complications. 2019 Sep;33(9):623–627.
4. Saigusa T, Watanabe K, Hada Y, Ishii K, Kameda W, Susa S, Ishizawa K, Ishihara H. Cardio-ankle vascular index is more closely associated than brachial-ankle pulse wave velocity with arterial damage and risk of cardiovascular disease in patients with diabetes. BMC Cardiovasc Disord. 2022 Aug 9;22(1):365.
5. Nakamura K, Iizuka T, Takahashi M, Shimizu K, Mikamo H, Nakagami T, Suzuki M, Hirano K, Sugiyama Y, Tomaru T, Miyashita Y, Shirai K, Noike H. Association between cardio-ankle vascular index and serum cystatin C levels in patients with cardiovascular risk factor. J Atheroscler Thromb. 2009 Aug;16(4):371–379.
6. Kubozono T, Miyata M, Ueyama K, et al. Association between arterial stiffness and estimated glomerular filtration rate in the Japanese general population. J Atheroscler Thromb. 2009;16(6):840-845.
7. Namikoshi T, Fujimoto S, Yorimitsu D, Ihoriya C, Fujimoto Y, Komai N, Sasaki T, Kashihara N. Relationship between vascular function indexes, renal arteriolosclerosis, and renal clinical outcomes in chronic kidney disease. Nephrology (Carlton). 2015 Sep;20(9):585–590.
8. Kurata M, Okura T, Watanabe S, Irita J, Enomoto D, Johtoku M, Miyoshi K, Koresawa M, Fukuoka T, Higaki J. Effects of amlodipine and candesartan on arterial stiffness estimated by cardio-ankle vascular index in patients with essential hypertension: A 24-week study. Curr Ther Res Clin Exp. 2008 Oct;69(5):412–422.
9. Miyoshi T, Murakami T, Sakuragi S, Doi M, Nanba S, Mima A, Tominaga Y, Oka T, Kajikawa Y, Nakamura K, Ito H. Comparable effect of aliskiren or a diuretic added on an angiotensin II receptor blocker on augmentation index in hypertension: a multicentre, prospective, randomized study. Open Heart. 2017 Mar 11;4(1):e000591.
10. Maliha G, Townsend RR. A study of the VaSera arterial stiffness device in US patients. J Clin Hypertens (Greenwich). 2017 Jul;19(7):661–668.
11. Mills CE, Govoni V, Faconti L, Casagrande ML, Morant SV, Crickmore H, Iqbal F, Maskell P, Masani A, Nanino E, Webb AJ, Cruickshank JK. A randomized, factorial trial to reduce arterial stiffness independently of blood pressure: Proof of concept? The VaSera trial testing dietary nitrate and spironolactone. Br J Clin Pharmacol. 2020 May;86(5):891–902.
12. Kario K, Okada K, Murata M, Suzuki D, Yamagiwa K, Abe Y, Usui I, Tsuchiya N, Iwashita C, Harada N, Okawara Y, Ishibashi S, Hoshide S. Effects of luseogliflozin on arterial properties in patients with type 2 diabetes mellitus: The multicenter, exploratory LUSCAR study. J Clin Hypertens (Greenwich). 2020 Sep;22(9):1585–1593.
13. Kario K, Nishizawa M, Kiuchi M, Kiyosue A, Tomita F, Ohtani H, Abe Y, Kuga H, Miyazaki S, Kasai T, Hongou M, Yasu T, Kuramochi J, Fukumoto Y, Hoshide S, Hisatome I. Comparative effects of topiroxostat and febuxostat on arterial properties in hypertensive patients with hyperuricemia. J Clin Hypertens (Greenwich). 2021 Feb;23(2):334–344

2) Secondary endpoints:

1. Geometric mean percent change in UACR at 12 and 24 weeks after protocol treatment initiation compared to that at baseline (ranked secondary endpoint)
2. Geometric mean percent change in blood biomarkers (pentosidine) and urinary biomarkers including type IV collagen, α1-MG, β2-MG, NGAL, NAG, L-FABP/creatinine ratio at 24 weeks after protocol treatment initiation compared to that at baseline

Rationale:

The purpose of this study was to evaluate the clinical effects of finerenone on biomarkers associated with the pathology of CKD with type 2 diabetes (1-2).

3) Other endpoints:

1. Changes in vital signs, including weight, BMI, eEV, blood pressure, pulse pressure, and pulse rate in the clinic, and blood pressure, pulse pressure, and pulse rate at home at 4, 12, and 24 weeks after protocol treatment initiation, from those at the baseline
2. Changes in blood test indices, including serum creatinine, eGFR, serum cystatin C*, serum potassium, HbA1c*, plasma aldosterone concentration**, plasma renin activity**) at 4, 12, and 24 weeks after protocol treatment initiation (*Evaluated only at baseline, and 12, and 24 weeks, **Evaluated only at baseline and 24 weeks), from those at the baseline
3. Changes in AI and % mean arterial pressure at 24 weeks after protocol treatment initiation from those at the baseline
4. Changes in cardiac function indices, including LVEF, septal e′, lateral e′, E, E/e′*, LVMI, LAD, and LAVI assessed by echocardiography 24 weeks after protocol treatment initiation, from those at the baseline
5. Changes in abundance of up to 181 proteins comprehensively analyzed by proteomic analysis (Target 96 panel) at 24 weeks after protocol treatment initiation compared to that at baseline (exploratory endpoint)

Rationale:

These parameters were set to evaluate the clinical effects of finerenone on the relevant indicators in the target population (1-2). They were also set to complement the primary endpoint assessing the effect of finerenone on cardiovascular function (3), as an exploratory evaluation of the effect of finerenone on cardiac function (4) and to complement the primary and secondary endpoints and further explore the comprehensive impacts of finerenone on cardiac, renal, and metabolic biomarkers (5).

4) Safety endpoints

Adverse events including hyperkalemia occurring after protocol treatment initiation.

Rationale:

This was set to evaluate the safety of finerenone.

## Research methodology (type and design of clinical research)

Investigator-initiated, multicenter, prospective, parallel-group, placebo-controlled, double-blind, randomized controlled trial.

## Research methodology (clinical research procedures)

After verifying the eligibility of patients who have provided written consent, patients who meet all inclusion criteria will be enrolled in the study. Patients will be randomized (allocated) to either the finerenone or placebo groups. They will undergo double-blind administration and observation at baseline (before the start of administration of the study drug) and at 4, 12, and 24 weeks after protocol treatment initiation, as well as prescribed tests (Figure). In principle, baseline tests will be conducted within 60 days of obtaining consent, and study drug administration will begin the following day.


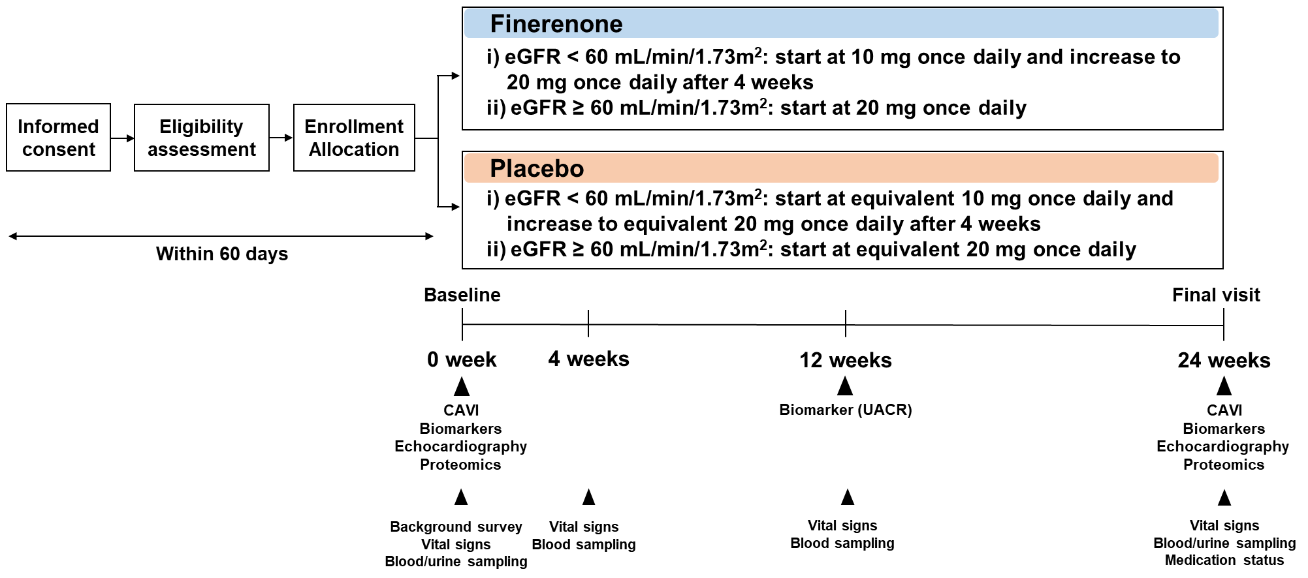


Figure (from Tanaka et al. Cardiovasc Diabetol. 2023;22:194)

## Method of study drug administration

### Method of study drug administration

Study participants will be instructed to orally self-administer finerenone or placebo once daily (preferably at approximately the same time in the morning every day). The study participants with a baseline eGFR < 60 mL/min/1.73 m^2^ will be administered a starting dose of 10 mg/day of finerenone (placebo group, the equivalent of 10 mg/day), the dose will be increased to 20 mg/day (placebo group, the equivalent of 20 mg/day) in principle 4 weeks after the first administration in accordance with the manufacturer’s latest instructions provided in the package insert. Moreover, study participants with a baseline eGFR ≥ 60 mL/min/1.73 m^2^ will be administered a starting dose of 20 mg/day of finerenone (placebo group, the equivalent of 20 mg/day). After starting or increasing the study drug dose, it will be reduced or suspended depending on the participants medical condition including changes in serum potassium levels and eGFR, referring to the manufacturer’s latest instructions provided in the package insert and the table below, and the details will be recorded in the case report forms (CRFs).

| Serum potassium level (mEq/L) | Dose adjustment |
| --- | --- |
| ≤ 4.8 | If the dose is 20 mg once daily, maintain dose  If the dose is 10 mg once daily, increase the dose to 20 mg once daily (only if eGFR has not decreased by > 30% since the previous measurement). |
| 4.8–5.5 | Maintain dose |
| > 5.5 | Discontinue |

Quoted from the 3rd edition of the package insert

### Study drug discontinuation criteria

Administration of the study drug will be discontinued under the following circumstances:

1. The principal investigator or co-investigator determines that continuing the study administration is difficult owing to the occurrence of an adverse event.
2. The principal investigator or co-investigator determines that the study drug administration should be discontinued for other reasons.

## Regulations for handling the study drug (management and distribution procedures)

### Centralized management of the study drug

Bayer Yakuhin, Ltd. will deliver the active and placebo test drugs to the Department of Cardiology at Saga University Hospital, where they will be temporarily stored. Each bottle will be assigned a different number, and a correspondence table of the active drug and placebo will be delivered simultaneously. After delivery, the study drug allocation officer will prepare a package for each case individually while checking the correspondence table.

### Delivery of the study drug to each medical institution

The drugs will be delivered to each participating medical institution by an independent Study Drug Management Officer in the Pharmacy Department of the Saga University Hospital.

### Prescribing and administering the study drug to study participants

The “study drug number” on the electric data capture (EDC) will be confirmed during allocation, and prescriptions will be provided in bottles. Moreover, any remaining prescribed study drugs remaining with the study participants after baseline will be available for administration during the observation period. All the remaining drugs, including unprescribed drugs, will be disposed collectively at the end of the study.

## Regulations on blinding

This study will be conducted as a double-blind study. After randomization, the study participants, investigators, and everyone else involved in the study will not know the allocation results until the data lock is complete. After the study is completed, the study drug allocation manager will unlock (unblind) the database after locking it.

The blind code will only be unlocked in emergency situations, including when appropriate medical treatment is needed owing to illness or when necessary to ensure the safety of the study participants. If the blind code is unlocked, the chief principal investigator will be notified immediately, and at that point, the participant’s participation in this study will be discontinued.

## Research discontinuation criteria

The chief principal investigator will consider discontinuing the study under the conditions mentioned below. If the study is discontinued, the principal investigators will promptly notify the participants of the discontinued study and provide appropriate medical care and other necessary measures. When the study is discontinued, the principal investigators will promptly report the discontinuation, the reason for it, and a summary of the results, in writing, to the administrator of the medical institution conducting the study.

1. The certified review board determines that the research should not continue
2. Doubts arise about the safety of research
3. Facts or information are obtained that undermine the ethical or scientific validity of the research
4. Facts or information are obtained that undermine the appropriateness of the research or the reliability of the results

### Discontinuation and termination procedures

If the chief principal investigator discontinues the entire study, he/she must state that and the reason for it in a “Notice of Discontinuation (Standard Form 11)” and submit it to the certified review board within 10 days of the discontinuation. The notice of discontinuation must state if any study participant needs observation.

In the event of discontinuation, the chief principal investigator will report the discontinuation and its reasons, in writing, to the principal investigators at each medical institution. The principal investigator will promptly inform the study participants during the study period and take appropriate measures, including changing treatment. If necessary, the chief principal investigator will seek the opinion of the certified review board on the timing and method of study termination considering the measures for the study participants. Even if a notice of discontinuation has been submitted, reports, including disease reports and regular reports, need to be submitted until the study is completed. Furthermore, if a change is needed in the implementation plan, a notification of the change to the implementation plan will be submitted.

When the chief principal investigator has submitted a notice of discontinuation and completed procedures for the study participants, he/she must, in principle, submit a comprehensive report within one year of the date of discontinuation or the end of the period for collecting data on all endpoints.

## Randomization procedure

Study participants will be randomly assigned using a central registration system. After confirming their eligibility, the principal investigators and co-investigators will promptly access the study’s website (EDC) and enter the required registration information. The information will be immediately verified on the EDC site, and if eligible, a patient identification number will be assigned. Then, dynamic allocation will be performed on the EDC.

Dynamic allocation to the finerenone and placebo groups will be performed using the minimization method in a 1:1 ratio with the following allocation stratification factors:

[Allocation factor]

Age at time of consent (< 70 years / ≥ 70 years)

Sex (female/male)

eGFR (< 60 mL/min/1.73 m^2^ / ≥ 60 mL/min/1.73 m^2^)*

Use of SGLT2 inhibitors at the time of consent (yes/no)

*Data obtained after obtaining consent will be prioritized; however, if such data cannot be obtained, data obtained within three months prior to obtaining consent may be used.

## Source documents

Source documents refer to the original records and data on the administration of drugs to study participants, clinical findings, observations, and other activities obtained through medical treatment, and prove the existence of the patients and their data. This study includes medical records, nursing records, prescription records, consent forms, clinical test data, and the clinical research contract between Bayer Yakuhin Ltd. and Saga University. Source documents will be maintained by each participating medical institution.

## Handling of CRFs

1. In this study, data will be collected using EDC, and the data collected will be compiled into CRFs. The results for the intensively measured blood biomarkers, urinary biomarkers, and proteomics analysis (all intensively measured test results will be blinded) will be compiled separately at the data center and ultimately included in the dataset. The chief principal investigator or the research office will send the results of the intensive measurements to the data center, where they will be entered into an Excel file. Two personnel will enter the data separately, and the values will be cross-referenced to confirm that they match perfectly.

2) Principal investigators, co-investigators, and research collaborators will input data into the EDC. Research collaborators may input data from the source documents into the EDC themselves if the source documents exist and their objectivity can be guaranteed.

3) After the initially entered data is saved on the server, the system records all revision history (date and time of revision, person who made the revision, data before and after revision, reason for revision).

4) Once all data entry into the EDC has been completed, the principal investigator will verify that the content is accurate.

## CRF content confirmation and inquiries

1. The data center will verify the following items for the entered CRF and centralized testing results (blood biomarkers, urinary biomarkers).
2. Input errors
3. Consistency with the current research plan
4. Consistency of CRF contents
5. Outliers in blood and urinary biomarkers
6. The data center will compile the points to be inquired about and send a data inquiry form to the principal investigator by email or via EDC.
7. The principal investigator, co-investigator, or research collaborator will promptly enter or modify the CRF on the EDC and notify the data center if no modification is required in response to the inquiry.

# **Inclusion criteria**

## Study participants

Patients with CKD and type 2 diabetes

## Inclusion criteria

Patients who meet all of the following criteria are eligible:

1. Patients who provide written informed consent to participate in this study
2. Patients aged ≥ 20 years at the time of consent (regardless of sex)
3. Patients with type 2 diabetes
4. Patients with CKD meeting both the following criteria:
   1. eGFR, 25–90 mL/min/1.73 m^2^

II. UACR, 30–3500 mg/g.cr.

1. Patients who have not changed their medication for type 2 diabetes or CKD in the 4 weeks prior to providing consent

Inclusion criteria rationale:

1. This was set to comply with the Clinical Research Act.
2. This was set to target people aged ≥ 20 years who can individually provide consent.

3) to 5) were set to select cases that met the objectives of this study.

## Exclusion criteria

Patients who meet any of the following criteria will be excluded from this study:

1. Patients currently on MRA, including finerenone, or those who have been administered it within the 4 weeks prior to providing informed consent
2. Patients with a history of hypersensitivity to finerenone
3. Patients with HbA1c ≥ 10%
4. Patients with serum potassium levels ≥ 4.9 mEq/L
5. Patients with NYHA class II–IV HFrEF (LVEF ≤ 35%)
6. Patients with poorly controlled hypertension including those with systolic blood pressure ≥ 170 mmHg, diastolic blood pressure ≥ 110 mmHg, and hypertensive emergency
7. Patients with a history of ischemic stroke, acute coronary syndrome, cardiovascular surgery or percutaneous treatment, or hospitalization due to worsening heart or renal failure within the 8 weeks prior to providing consent
8. Patients scheduled to undergo coronary revascularization or other surgical or percutaneous treatment for cardiovascular disease during the observation period
9. Patients scheduled to undergo treatment, including electrical defibrillation, cardiac resynchronization therapy, or pacemaker implantation, during the observation period
10. Patients scheduled to undergo dialysis or kidney transplantation during the observation period
11. Patients with severe liver dysfunction (Child-Pugh class C)
12. Patients administered itraconazole, ritonavir-containing preparations, atazanavir, darunavir, fosamprenavir, cobicistat-containing preparations, or clarithromycin
13. Patients with Addison’s disease
14. Patients with active infectious disease
15. Patients with pregnancy, possibly pregnancy, or breastfeeding
16. Patients deemed unsuitable for this study by the principal investigator or co-investigator including patients with renal artery stenosis, one kidney, and active malignant tumor

Exclusion criteria rationale:

1), 8) to 10) were set owing to their expected impact on the endpoints.

2) to 4) and 11) to 15) were set to ensure patient safety.

5) This was set because the guidelines recommend the administration of MRA, the same type of drug as the study drug.

6) and 7) were set because of their expected impact on ensuring patient safety and on the endpoints.

16) This was set assuming that the principal investigator and/or co-investigator would decide the suitability of a patient as a participant from a scientific and ethical perspective.

## Discontinuation criteria for study participants

The study will be discontinued for the circumstances mentioned below. If it is discontinued, the reason and the discontinuation date will be entered in the CRF. If the discontinuation date is within the 4-, 12-, or 24-weeks allowance, tests will be performed to the extent possible.

1. A study participant declines to participate in the study or withdraws consent.
2. Patient transfer to another hospital makes visiting the medical institution where the study is being conducted difficult for the participant.
3. Discovery of a participant not meeting the inclusion criteria or meeting the exclusion criteria after the study initiation.
4. The principal investigator or co-investigator determines that continuation of the study is difficult owing to the occurrence of an adverse event.
5. The principal investigator or co-investigator determines that continuation of the study is difficult owing to worsening of a primary disease or complications.
6. The study is unlocked.
7. The principal investigator or co-investigator determines it is appropriate to discontinue participation in the study for other reasons.

## Treatment of study participants discontinuing the study

For study participants discontinuing, except those under conditions 1–4 mentioned below, a discontinuation examination will be performed to the extent possible, and they will cease to participate in the study. For the examination schedule, refer to the relevant section of “observation and testing items.” Even if the observations specified in the research plan cannot be carried out owing to reasons including transfer to another hospital, outcome information will be obtained from the transferred hospital to the extent possible, and the obtained information will be included in the medical records including the chart along with the method and date of acquisition.

1. The study participant dies
2. The study participant withdraws consent for follow-up after discontinuation
3. The study participant is discovered to be ineligible after study initiation
4. Tracking the participant is difficult for other reasons

# **Treatment of the study participants**

## Observation and testing schedule

The observation and testing items and schedule for this study are presented below. The study investigators will conduct observation and testing according to the schedule. However, for tasks that can be completed by research collaborators, including obtaining patient background data and conducting clinical tests, the research collaborators may perform them under the supervision of the principal investigator or co-investigator.

Table 1. Observation and testing schedule

| Timing  Testing item | Consent  acquisition | Eligibility determination,  registration, and allocation | Observation period ^1)^ | | | |
| --- | --- | --- | --- | --- | --- | --- |
|  |  |  | Baseline ^2) 3)^ | Week 4 | Week 12 | Week 24 |
|  |  |  | Day 0 | Day 28  [21–35] | Day 84  [70–98] | Day 168  [154–182] |
| Obtaining consent | ● |  |  |  |  |  |
| Eligibility determination, ^4)^ Information required for registration and allocation ^5)^ |  | ● |  |  |  |  |
| Patient background information |  | ● |  |  |  |  |
| Physical examination findings ^6)^ |  |  | ● | ● | ● | ● |
| CAVI |  |  | ● |  |  | ● |
| UACR |  |  | ●^7）^ |  | ● | ● |
| Serum creatinine, eGFR, serum cystatin C |  |  | ●^7）^ | ●^8)^ | ● | ● |
| Other biomarkers (blood and urine) ^9,10)^ |  |  | ● |  |  | ● |
| Proteomics ^10)^ |  |  | ● |  |  | ● |
| Blood test ^11)^ |  |  | ●^12)^ | ●^13)^ | ●^14)^ | ●^15)^ |
| Cardiac ultrasound examination ^16)^ |  |  | ●^17)^ |  |  | ● |
| Concomitant medications ^18)^ |  |  | ● |  |  | ● |
| Compliance with the study drug |  |  |  |  |  |  |
| Adverse events |  |  |  |  |  |  |

[ - ]: Allowance range

1) If the discontinuation date is within the 4-, 12-, or 24-weeks allowance, observations and tests will be conducted to the extent possible.

2) In principle, a baseline test will be conducted within 60 days of obtaining consent, and administration of the study drug will begin the next day.

3) If eligibility determination and baseline are conducted on the same day, the same data can be used for overlapping items.

4) When determining eligibility, the eGFR, UACR, HbA1c, serum potassium, and LVEF levels will also be recorded and entered into the EDC. Data obtained after obtaining consent will be prioritized for eGFR, UACR, and LVEF levels; however, in the absence of this information, data obtained within 3 months prior to obtaining consent can be used.

5) At the time of registration and allocation, data on date of consent, age at the time of providing consent, date of birth, sex, use of SGLT2 inhibitors, and eGFR will be recorded. For eGFR, data obtained after obtaining consent will be prioritized; however, in the absence of this information, data from within 3 months prior to consent can be used.

6) Height (data obtained after obtaining consent will be prioritized; however, in the absence of this information, data from within 3 months prior to consent can be used. Height will only be measured at baseline.), weight, blood pressure (clinic/home*), and pulse rate (clinic/home*).

^*Optional.^

7) For UACR and serum cystatin C, baseline data will be prioritized; however, if the baseline data is unavailable, data within 3 months from the baseline may be used.

8) Serum creatinine and eGFR only.

9) Blood (pentosidine) and urine (type IV collagen, α1-MG, β2-MG, NGAL, NAG, and L-FABP creatinine ratio).

10) Measurement will be outsourced.

11) Hemoglobin, hematocrit, serum albumin, serum potassium, HbA1c, plasma aldosterone concentration, plasma renin activity

12) HbA1c, data obtained after obtaining consent will be prioritized; however, in the absence of this information, data at the time of eligibility determination may be used.

13) Only serum potassium levels are measured.

14) Only serum potassium and HbA1c levels are measured.

15) Only serum potassium and HbA1c levels, plasma aldosterone concentration, and plasma renin activity are measured.

16) This is an optional item.

17) Baseline data will be prioritized; however, if the baseline data is unavailable, data within 3 months from the baseline may be used.

18) In principle, no new administration of SGLT2 inhibitors and no changes in the dosage and administration of concomitant medications will be implemented during the observation period.

## Patient registration

1. The principal investigator or co-investigator will obtain written consent from the patient, determine eligibility via any tests or interviews required to confirm eligibility, and then register and allocate the patient.
2. The eligibility and allocation test items will be entered into the EDC, and registration and allocation will be performed based on the eligibility assessment results. Registered study participants (including those determined to be “ineligible” by the EDC) will be assigned a patient identification number, and the group to which the eligible cases will be assigned will be displayed.
3. The medical institution conducting the study will create an “anonymization correspondence table” and enter numbers in it to prevent patient mix-ups. The principal investigator, co-investigator, or research collaborator will maintain the original data at the medical institution.

### Eligibility determination, registration, and allocation testing items

Upon eligibility determination, the following will be recorded and entered into the EDC:

・eGFR*, UACR* levels

・HbA1c, serum potassium levels

・LVEF* values

Then, at the time of registration and allocation, the following will be recorded and entered into the EDC:

・Date of consent, age at the time of consent, date of birth, and sex

・Use of SGLT2 inhibitors, eGFR* level

*Data obtained after obtaining consent will be prioritized; however, in the absence of this information, data from within 3 months prior to consent can be used

### Patient background

Duration of diabetes, presence or absence of complications/history of hypertension, dyslipidemia, ischemic heart disease, stroke, lower limb arteriosclerosis obliterans, or heart failure, primary underlying chronic renal failure disease including diabetic and non-diabetic nephropathy (chronic glomerulonephritis, nephrosclerosis, polycystic kidney disease, collagen disease-related nephropathy, or others)

## Scheduled observation and testing items

1. Baseline (Day 0)

In principle, a baseline test will be conducted within 60 days of obtaining consent, and administration of the study drug will begin the next day. If eligibility determination and baseline are conducted on the same day, the same data can be used for overlapping items.

1. Physical examination findings:

Height*, weight, clinic blood pressure (systolic and diastolic), clinic pulse rate

Optional: Home blood pressure (systolic and diastolic), home pulse rate

1. CAVI:

The device that measures CAVI (Basera) is approved as a medical device (blood pressure pulse wave inspection device) and is used to measure non-invasive blood pressure, electrocardiogram, phonocardiogram, and pulse wave chart to test arterial distensibility or blood flow disorder in the blood vessels of the lower limbs. Measurements are performed with reference to Appendix 2 (Basera Test Guide).

1. UACR** levels
2. Serum creatinine, eGFR, serum cystatin C** levels
3. Other biomarkers: (Outsourced measurement)

Blood (Pentosidine)

Urine (type IV collagen, α1-MG, β2-MG, NGAL, NAG, and L-FABP creatinine ratio).

1. Proteomics (outsourced measurement)
2. Blood tests:

Hemoglobin, hematocrit, serum albumin and potassium levels, HbA1c***, plasma aldosterone concentration, plasma renin activity

1. Cardiac ultrasound examination (optional)****:

LVEF, septal e′, lateral e′, mitral orifice blood flow velocity waveform (E), E/e′, LVMI, LAVI

1. Concomitant medications:

ACE inhibitors, ARBs, ARNIs, β-blockers, calcium antagonists, diuretics, statins, insulin, metformin, SGLT2 inhibitors, GLP-1 receptor agonists, DPP-4 inhibitors, iron preparations, ESAs, HIF-PH inhibitors, and hyperkalemia medications

During the observation period, in principle, no new administration of SGLT2 inhibitors and no changes in the dosage and administration of concomitant medications will be implemented.

* Data obtained after obtaining consent will be prioritized; however, in the absence of this information, data from within 3 months prior to consent can be used.

**Baseline data will be prioritized; however, if the baseline data is unavailable, data within 3 months from the baseline may be used.

*** Baseline data will be prioritized; however, if the baseline data is unavailable, data from the time of eligibility determination may be used.

**** Baseline data will be prioritized; however, if the baseline data is unavailable, data from 3 months prior to obtaining consent up to baseline may be used.

1. Week 4 (Day 28, allowable range for observation and testing: Days 21–35)
2. Physical examination findings:

Weight, clinic blood pressure (systolic and diastolic), clinic pulse rate

Optional: Home blood pressure (systolic and diastolic), home pulse rate

1. Serum creatinine levels and eGFR
2. Blood test: serum potassium levels
3. Week 12 (Day 84, allowable range for observation and testing: Days 70–98)
4. Physical examination findings:

Weight, clinic blood pressure (systolic and diastolic), clinic pulse rate

Optional: Home blood pressure (systolic and diastolic), home pulse rate

1. UACR levels
2. Serum creatinine, eGFR, serum cystatin C levels
3. Blood tests: serum potassium and HbA1c levels
4. Week 24 (Day 186, allowable range for observation and testing: Days 154–182)
5. Physical examination findings:

Weight, clinic blood pressure (systolic and diastolic), clinic pulse rate

Optional: Home blood pressure (systolic and diastolic), home pulse rate

1. CAVI:
2. The device that measures CAVI (Basera) is approved as a medical device (blood pressure pulse wave inspection device) and is used to measure non-invasive blood pressure, electrocardiogram, phonocardiogram, and pulse wave chart to test arterial distensibility or blood flow disorder in the blood vessels of the lower limbs. Measurements are performed using Appendix 2 (Basera Test Guide) as a reference.
3. UACR levels
4. Serum creatinine, eGFR, serum cystatin C levels
5. Other biomarkers: (Outsourced measurement)

Blood (Pentosidine)

Urine (type IV collagen, α1-MG, β2-MG, NGAL, NAG, and L-FABP creatinine ratio).

1. Proteomics (outsourced measurement)
2. Blood tests:

Serum potassium and HbA1c levels, plasma aldosterone concentration, plasma renin activity

1. Cardiac ultrasound examination (optional):

LVEF, septal e′, lateral e′, mitral orifice blood flow velocity waveform (E), E/e′, LVMI, LAVI

1. Concomitant medications:

ACE inhibitors, ARBs, β-blockers, calcium antagonists, diuretics, statins, insulin, metformin, SGLT2 inhibitors, GLP-1 receptor agonists, DPP-4 inhibitors, iron preparations, ESAs, HIF-PH inhibitors, and hyperkalemia medications

## *The method for measuring blood pressure at home should be based on the blood pressure handbook provided by the Japanese Society of Hypertension, and in principle, morning measurement results should be collected.

## Observation and testing items as-needed

1. Compliance with the study drug

Date of initiating administration, dosage (mg/day), reason for discontinuing administration, end date of administration, date of change in dosage (increase or decrease), changed dosage, reason, remaining quantity of medication

1. Adverse events (from consent to week 24):

Name of the adverse event, date of onset, severity, seriousness (serious/non-serious), presence or absence of causal relationship with the study (if “yes,” name of drug or test item suspected to be related), causal relationship with the study drug, predictability (known/unknown), outcome (recovered, improved, not recovered, with sequelae, death, unknown), and date of outcome

## Central testing

SRL, Inc. will centrally measure blood biomarkers (pentosidine) and urinary biomarkers (type IV collagen, α1-MG, β2-MG, NGAL, NAG, L-FABP creatinine ratio). SRL, Inc. will also collect and measure anonymized blood and urinary biomarker samples. Moreover, SRL, Inc. will collect and freeze samples for proteomics analysis, which will then be transported to a testing institution designated by the Department of Cardiology at Saga University Hospital while still anonymized.

Procedures for collecting, transporting, and measuring samples, as well as providing test results, will follow the standard operating procedures separately established by SRL, Inc. and the testing institutions designated by the Department of Cardiology at Saga University Hospital.

## Overview of the study drug

This study will use the test drugs provided by Bayer Yakuhin, Ltd listed below. For details, please refer to the latest package insert.

### Active drug

Generic name: Finerenone

Brand name: Kerendia tablets 10 mg/20 mg

Manufactured and sold by: Bayer Yakuhin Co., Ltd.

### Placebo

Tablets that look identical to the real medicine but contain no active ingredients

Ingredients: Tablet (lactose hydrate, microcrystalline cellulose, and magnesium stearate), film coating (hypromellose, talc, titanium oxide, yellow ferric oxide, and red ferric oxide)

### Efficacy and effectiveness

CKD with type 2 diabetes

However, this does not include patients with end-stage renal disease or those undergoing dialysis.

Caution (efficacy and effects)

1. This drug should be administered to patients receiving ACE inhibitors or ARBs, except when these treatments are not appropriate.
2. Because the administration of this drug may decrease eGFR, the appropriateness of administering this drug to patients with eGFR < 25 mL/min/1.73 m^2^ should be carefully considered after considering the risks and benefits.
3. In the Japanese subpopulation, the hazard ratio of this drug versus placebo for the renal composite endpoint, the primary endpoint of the global phase III study (Study 16244), was 0.911, while the hazard ratio of this drug versus placebo for renal failure, a component of the primary endpoint of the global phase III study (Study 16244), and for the renal composite endpoint, a secondary endpoint of the global phase III study (Study 17530) was > 1. The drug’s effect in preventing progression to renal failure may be weaker in Japanese patients as compared to that in the overall study population.
4. Select appropriate patients after thoroughly understanding the clinical results and the background of the patients enrolled in the clinical trial (primary disease, concomitant medications, renal function, and albuminuria).

### Dosage and administration

The usual dose of finerenone for adults is administered orally once daily at the following dosage:

eGFR, ≥ 60 mL/min/1.73 m^2^: 20 mg

eGFR < 60 mL/min/1.73 m^2^: Start administration at 10 mg and increase to 20 mg approximately 4 weeks after the administration initiation, depending on serum potassium levels and eGFR.

Caution (dosage and administration)

1. Serum potassium levels and eGFR should be measured 4 weeks after initiation of administration, resumption, or dose increase, and periodically thereafter; the dosage should be adjusted according to the table.

| Serum potassium level (mEq/L) | Dose adjustment |
| --- | --- |
| ≤ 4.8 | If the dose is 20 mg once daily, maintain dose;  If the dose is 10 mg once daily, increase dose to 20 mg once daily (only if eGFR has not decreased by > 30% since the previous measurement). |
| 4.8–5.5 | Maintain dose |
| > 5.5 | Discontinue |

After discontinuing administration, if serum potassium levels drop below 5.0 mEq/L, administration may be resumed at 10 mg once daily.

1. Bioequivalence of the 10 mg and 20 mg tablets has not been demonstrated; therefore, 10 mg tablets should not be used for the 20 mg dose.

### Usage precautions

- 1. Contraindications (do not administer to the following patients):

1. Patients with a history of hypersensitivity to any of the ingredients in this drug.
2. Patients receiving itraconazole, ritonavir-containing preparations, atazanavir, darunavir, fosamprenavir, cobicistat-containing preparations, or clarithromycin
3. Patients with serum potassium level > 5.5 mEq/L at the start of the drug administration as their hyperkalemia may worsen
4. Patients with severe liver dysfunction (Child-Pugh class C)
5. Patients with Addison’s disease as the drug may worsen the patient’s condition
6. Important basic notes
7. Because hyperkalemia may occur, in principle, administration should be initiated in patients with serum potassium levels ≤ 4.8 mEq/L, and the serum potassium levels and eGFR should be measured 4 weeks after initiating, resuming, or increasing administration, and then periodically thereafter. Moreover, in patients with serum potassium level > 4.8 mEq/L at the start of administration, additional serum potassium measurements should be considered prior to the start of administration 4 weeks later, depending on the serum potassium level and the patient’s condition.
8. Because eGFR may decrease early during the administration of this drug, it should be administered with caution to avoid deterioration of renal function.
9. Dizziness due to the hypotensive effect of this drug may occur. Therefore, caution should be exercised by patients when working at heights, driving a car, or operating dangerous machinery.

### Caution for patients with specific backgrounds

### Patients with complications or medical history

### Patients with serum potassium levels of 5.0–5.5 mEq/L

The appropriateness of administering this drug should be judged carefully as it may exacerbate hyperkalemia.

### Patients at high risk of developing hyperkalemia

Serum potassium levels should be monitored more frequently in the patients with the following conditions as they may be at increased risk of developing hyperkalemia:

・Low eGFR

・High serum potassium

・History of hyperkalemia

### Patients with renal dysfunction

### Patients with severe renal dysfunction

Because administration of this drug may decrease eGFR, the appropriateness of administering this drug should be carefully considered in patients with eGFR < 25 mL/min/1.73 m^2^. Moreover, if the patient develops end-stage renal failure or requires dialysis during administration of this drug, the administration of this drug should be discontinued owing to the risk of developing hyperkalemia.

### Patients with liver dysfunction

### Patients with severe liver dysfunction (Child-Pugh class C)

Do not administer this drug. This drug has been excluded in clinical trials as administration may increase blood levels of this drug.

### Patients with moderate liver dysfunction (Child-Pugh class B)

Measure serum potassium levels more frequently depending on the patient’s condition. Blood concentrations of this drug may increase.

### Those with reproductive potential

Females of childbearing potential should be instructed to use appropriate contraception during administration.

### Pregnant women

Pregnant women or women who may be pregnant should only be administered if the therapeutic benefits outweigh the risks. In animal experiments (rats), teratogenicity (double aortic arch) was observed at a systemic exposure level approximately 25 times higher than that in humans, embryo-fetal toxicity (reduced fetal weight) was observed at systemic exposure level approximately 19 times higher than that in humans, and effects on fertility (low ovarian weight) were observed at systemic exposure level approximately 17 times higher than that in humans. In animal experiments (rats), a mild increase in spontaneous motor activity in the fetus, likely owing to exposure during pregnancy, was observed at a systemic exposure level of the mother approximately 4 times higher than that in humans.

### Breastfeeding women

Avoid administration to patients who are breastfeeding. Animal experiments (rats, intravenous administration) have revealed that the compound is transferred into breast milk. Moreover, in rat mothers, obvious adverse effects (increased mortality) were observed in lactating infants at systemic exposure levels approximately 4 times higher than that in humans.

### Children

No clinical trials have been conducted on children.

### Contraindications for concomitant administration (do not use together)

| Drug name | Clinical symptoms and treatment methods | Mechanism and risk factors |
| --- | --- | --- |
| Itraconazole (Itrizol)  Ritonavir-containing preparations (Norvir, Kaletra)  Atazanavir (Reyataz)  Darunavir (Prezista, PrezistaNive)  Fosamprenavir (Lexiva)  Cobicistat-containing preparations (Genvoya, Stribild, Simtuza, Prezicovic)  Clarithromycin(Claris, Claricid) | Risk of significant increase in blood concentration of this drug | Strong CYP3A inhibition reduces the clearance of this drug. |

### Precautions for concomitant administration (caution when using together)

| Drug name | Clinical symptoms and treatment methods | Mechanism and risk factors |
| --- | --- | --- |
| Moderate CYP3A inhibitor  erythromycin  Verapamil  Fluconazole, etc.  Weak CYP3A inhibitor  Amiodarone  Fluvoxamine, etc. | Owing to the risk of increase in the blood concentration of this drug, the patient’s condition, including serum potassium levels, should be carefully observed, particularly when starting this drug and adjusting the dose. | Inhibition of CYP3A decreases the clearance of this drug. |
| Strong or moderate CYP3A inducer  Rifampin  Carbamazepine  Phenytoin  Phenobarbital  Efavirenz  Mitotane  Foods containing St. John’s Wort | As the blood concentration of this drug may decrease significantly and its effectiveness may be reduced, consider substituting with an agent with no or weaker CYP3A-inducing effect. | Induction of CYP3A increases the clearance of this drug. |
| Spironolactone  Triamterene  Potassium canrenoate  Eplerenone  Esaxerenone | Owing to the risk of increased serum potassium levels and hyperkalemia, concomitant use should only be performed when necessary for treatment. If concomitant use is required, the patient’s condition should be carefully monitored, for example, by measuring serum potassium levels more frequently. | Risk of increased potassium retention |
| Potassium supplements | Owing to the increased risk of elevated serum potassium levels and hyperkalemia, caution should be exercised, for example, by measuring serum potassium levels more frequently. | Risk of increased potassium retention |
| Sulfamethoxazole-trimethoprim | Owing to the increased risk of elevated serum potassium levels and hyperkalemia, caution should be exercised by measuring serum potassium levels more frequently or using similar methods. Alternatively, discontinuation of this drug should be considered. | Risk of increased potassium retention |
| Grapefruit-containing foods | Be careful not to ingest these as they may increase blood levels of the drug. | Inhibition of CYP3A decreases the clearance of this drug. |
| Lithium  Lithium carbonate | Since lithium poisoning may occur, monitor blood lithium levels carefully. | Although the exact mechanism is unknown, this may occur by promoting sodium excretion, as sodium ion deficiency has been proposed to promote the retention of lithium ions. |
| Nonsteroidal anti-inflammatory drugs  Indomethacin, etc. | Hyperkalemia may occur in patients with renal impairment. | Although the exact mechanism is unknown, inhibition of prostaglandin production likely reduces the glomerular filtration rate, resulting in potassium retention and increased serum potassium levels. |
| Mitotane | May inhibit the effects of mitotane. | Although the exact mechanism is unknown, previous studies have reported that the efficacy of mitotane is inhibited by a similar drug (spironolactone). |

### Side effects

Adverse reactions may occur; therefore, the patients will be closely monitored. If any abnormalities are identified, appropriate measures will be taken including discontinuation of the drug.

In a global phase III study (Study 16244/FIDELIO-DKD) targeting patients with CKD and type 2 diabetes administered standard treatment with ACE inhibitors or ARBs, adverse events were observed in 646/2,827 cases (22.9%) in the finerenone group and 449/2,831 cases (15.9%) in the placebo group. The main events in the finerenone and placebo groups were hyperkalemia in 286 (10.1%) and 114 (4.0%) cases, respectively; increased blood potassium in 53 (1.9%) and 22 (0.8%) cases, respectively; increased blood creatinine in 44 (1.6%) and 39 (1.4%) cases, respectively; and hypotension in 42 (1.5%) and 22 (0.8%) cases, respectively.

Serious adverse events were observed in 48 cases (1.7%) in the finerenone group and 34 cases (1.2%) in the placebo group. The main events in the finerenone and placebo groups were hyperkalemia in 24 (0.8%) and 5 (0.2%) cases, respectively; acute kidney injury in 9 (0.3%) and 6 (0.2%) cases, respectively; and hypotension in 3 (0.1%) and 2 (< 0.1%) cases, respectively.

Adverse events leading to discontinuation of the study drug were observed in 94 cases (3.3%) in the finerenone group and 59 cases (2.1%) in the placebo group. The main events in the finerenone and placebo groups were hyperkalemia in 43 (1.5%) and 12 (0.4%) cases, respectively; increased blood potassium in 10 (0.4%) and 4 (0.1%) cases, respectively; increased blood creatinine in 4 (0.1%) and 2 (< 0.1%) cases, respectively; renal disorder in 4 (0.1%) and 3 (0.1%) cases, respectively; pruritus in 4 (0.1%) and 1 case (< 0.1%), respectively; diarrhea in 3 (0.1%) and 8 (0.3%) cases, respectively; acute kidney injury in 3 (0.1%) and 3 (0.1%) cases, respectively; and rash in 3 (0.1%) and 1 case (< 0.1%), respectively.

No side effects leading to death (excluding efficacy evaluation events) were reported. (Source: Interview Form.)

　In an international phase III study (Study 17530/FIGARO-DKD) in patients with CKD and type 2 diabetes receiving standard treatment with ACE inhibitors or ARBs, adverse events were observed in 560/3,683 cases (15.2%) in the finerenone group and 413/3,658 cases (11.3%) in the placebo group. The main events in the finerenone and placebo groups were hyperkalemia in 210 (5.7%) and 100 (2.7%) cases, respectively; hypotension in 50 (1.4%) and 11 (0.3%) cases, respectively; increased blood potassium in 32 (0.9%) and 17 (0.5%) cases, respectively; and decreased glomerular filtration rate in 28 (0.8%) and 18 (0.5%) cases, respectively.

Serious adverse events were observed in 35 cases (1.0%) in the finerenone group and 27 cases (0.7%) in the placebo group. The main events in the finerenone and placebo groups were hyperkalemia in 15 (0.4%) and 3 (<0.1%) cases, respectively; and acute kidney injury in 9 (0.2%) and 3 (< 0.1%) cases, respectively. Adverse events leading to discontinuation of the study drug were observed in 97 cases (2.6%) in the finerenone group and 53 cases (1.4%) in the placebo group. The main events in the finerenone and placebo groups were hyperkalemia in 28 (0.8%) and 5 (0.1%) cases, respectively; diarrhea in 10 (0.3%) and 3 (< 0.1%) cases, respectively; increased blood potassium in 7 (0.2%) and 0 cases, respectively; decreased glomerular filtration rate in 6 (0.2%) and 1 case (< 0.1%), respectively; acute kidney injury in 5 (0.1%) and 1 case (< 0.1%), respectively; and rash in 4 (0.1%) and 4 (0.1%) cases, respectively. No adverse events leading to death (excluding efficacy evaluation events) were reported in the finerenone group, while 1 case (< 0.1%) each of hepatocellular carcinoma and death (details unknown) occurred in the placebo group. (Source: Interview Form.)

### Serious side effects

Hyperkalemia (8.8%)

### Other side effects

|  | > 1% | < 1% |
| --- | --- | --- |
| Metabolic and nutritional disorders |  | Hyponatremia, hyperuricemia |
| Vascular disorders | Low blood pressure |  |
| Laboratory tests | Decreased glomerular filtration rate |  |

## Concomitant medications during the observation period

Concomitant use of itraconazole, preparations with ritonavir, atazanavir, darunavir, fosamprenavir, preparations with cobicistat, and clarithromycin is prohibited.

In both groups, the physician in charge will make a comprehensive clinical judgment, including the condition of the study participants, and in principle, no restrictions will exist on the use of drugs other than the study drug (drugs for type 2 diabetes, chronic renal failure, and other comorbid diseases); however, in principle, no new administration of SGLT2 inhibitors will be implemented during the observation period. Moreover, the dosage and administration of drugs other than the study drug will not be changed in principle; however, changes at the discretion of the attending physician will be permitted depending on the participant’s medical condition.

# **Efficacy and safety evaluation**

## Primary endpoint

　　Change in CAVI at 24 weeks after the protocol treatment initiation compared to that at baseline.

## Secondary endpoints

1. Proportional change in geometric mean of UACR at 12 and 24 weeks after protocol treatment initiation compared to that at baseline (important secondary endpoint)
2. Proportional change in geometric mean of blood biomarkers (pentosidine) and urinary biomarkers (type IV collagen, α1-MG, β2-MG, NGAL, NAG, L-FABP creatinine ratio) at 24 weeks after the protocol treatment initiation compared to that at baseline

## Other endpoints

1. Changes from baseline in vital signs, including weight, BMI, eEV, blood pressure, pulse pressure, and pulse rate in the clinic, and blood pressure, pulse pressure, and pulse rate at home at 4, 12, and 24 weeks after protocol treatment initiation
2. Changes from baseline in blood test indices, including serum creatinine, eGFR, serum cystatin C*, serum potassium, HbA1c*, plasma aldosterone concentration**, plasma renin activity** at 4, 12, and 24 weeks after protocol treatment initiation (*evaluated only at baseline, and 12, and 24 weeks, **evaluated only at baseline and 24 weeks)
3. Changes from baseline in AI and % mean arterial pressure at 24 weeks after protocol treatment initiation
4. Changes from baseline in cardiac function indices, including LVEF, septal e', lateral e', E, E/e'*, LVMI, LAD, LAVI assessed by echocardiography 24 weeks after protocol treatment initiation
5. Changes in abundance of up to 181 proteins comprehensively analyzed by proteomic analysis (Target 96 panel) at 24 weeks after protocol treatment initiation as compared to that at baseline (exploratory endpoint)

## Safety endpoints

Adverse events including hyperkalemia occurring after protocol treatment initiation.

### Assessment of adverse events

The methods for evaluating the severity, seriousness, predictability, causal relationship to the study, and causal relationship of adverse events to the study drug are listed below.

### Severity

The severity of adverse events will be stratified as mild, moderate, or severe according to the following criteria:

- 1. Mild: does not interfere with daily activities
  2. Moderate: interferes with daily activities
  3. Severe: impaired ability to carry out normal daily activities

### Seriousness

The seriousness will be judged as either serious or non-serious. Serious refers to any of the conditions listed below.

- 1. Death
  2. Illnesses that may lead to death
  3. Illnesses that require hospitalization or an extended hospital stay at a medical institution for treatment
  4. Disability
  5. Diseases that may lead to disabilities
  6. Illnesses that are similar in seriousness to 3) to 5) and illnesses that are fatal or have the potential to lead to death
  7. Congenital diseases or abnormalities in later generations

### Causal relationship with the conduct of the study

Adverse events will be determined as causally related to the study treatment as either related or unrelated.

“Related” indicates that the relationship between the adverse event and the implementation of the study cannot be denied.

### Causal relationship to the study drug

Adverse events will be determined as causally related to the study drug as either related or unrelated.

“Related” indicates that the relationship between the adverse event and the administration of the study drug cannot be denied. If the relationship cannot be determined to be “related,” it will be categorized as “unrelated.”

### Predictability

Predictability will be determined as either a predictable adverse event (known) or an unpredictable adverse event (unknown). If an adverse event is not described in the documents listed below and cannot be predicted, it will considered an unpredictable adverse event.

The latest Finerenone package insert

The side effects information listed in the 3rd edition of the Finerenone package insert (revised September 2022) is as described in “4.6.9 Side Effects.”

### Outcome and outcome date

The outcome of adverse events will be determined as recovered, improved, not recovered, with sequelae, death, or unknown.

The outcome date will be the date on which the principal investigator or co-investigator confirms the outcome. If the outcome is not one of the following, i.e., improved, recovered, with sequelae, or death, and is not recovered or unknown, follow-up will continue until the end of the observation period for that study participant, and the outcome and outcome date will be entered into the EDC.

## Handling adverse events

### Definitions of adverse events, diseases, and side effects

An adverse event is any untoward or unintended sign (including laboratory abnormalities), symptom, or illness occurring in a study participant, irrespective of being causally related to the study.

Illnesses include illnesses, disabilities, or deaths or infectious diseases suspected to be attributable to the study, as well as abnormal clinical test results and various symptoms.

Side effects are defined as adverse events that have been proposed to be “related” to the study drug.

In this study, we will collect data on all adverse events that occur in study participants from the date of consent to the end of the observation period.

## Response to occurrence of adverse event

### Procedures for study participants

If an adverse event occurs, the principal investigator or co-investigator will take appropriate measures to ensure the safety of the study participant, such as discontinuing treatment and administration of the study drug, if necessary. If treatment becomes necessary, the study participant will be informed of this.

### Reporting adverse events

When an adverse event occurs, the principal investigator or sub-investigator will promptly evaluate the severity, causal relationship to the study, causal relationship to the study drug, predictability, and outcome. If the event is deemed to be an illness, the principal investigator will report it in accordance with “5.6.3 Reporting of Illnesses” The representative principal investigator will report any serious adverse events related to the study drug to Bayer Yakuhin, Ltd. within 24 hours after the representative principal investigator has become aware of them.

The principal investigator or co-investigator will enter the following information on the adverse event into the EDC:

- 1. Adverse event name
  2. Date of onset
  3. Severity
  4. Seriousness (serious/non-serious)
  5. The presence/absence of a causal relationship to the implementation of the study (if “present,” the names of drugs or test items suspected to be related)
  6. Causal relationship to the study drug
  7. Predictability (known/unknown)
  8. Outcome (recovered, improved, not recovered, with sequelae, death, unknown)
  9. Outcome date

### Reporting illnesses

As this study falls under the category of specific clinical research other than specific clinical research using unapproved or off-label drugs, the chief principal investigator must report any illnesses or other issues to the certified review board in accordance with the provisions of the Clinical Research Act (Act No. 16 of 2017) and the Enforcement Regulations of the Clinical Research Act (Ministry of Health, Labour and Welfare Ordinance No. 47 of 2022) in the event of an illness or other issues.

　If the principal investigator becomes aware of a serious illness, he/she shall promptly report this to the administrator of the medical institution conducting the study and the chief principal investigator, who shall then report it to the certified review board. Reporting this to the administrator of the medical institution conducting the study before reporting to the certified review board is not necessary, and the order of reporting can be changed depending on the situation.

If the chief principal investigator becomes aware of any illness other than serious illness, he/she will report this to the administrator of the medical institution conducting the research once a year and then make regular reports to the certified review board. Moreover, the chief principal investigator will report this to Bayer Yakuhin, Ltd. within 24 hours of receiving the regular reports. Other principal investigators will follow the reporting obligations of each medical institution as a standard.

For serious adverse events that do not fall under the above-mentioned illnesses, the principal investigator will promptly convey the information to the chief principal investigator in accordance with the procedures of the institution to which the chief principal investigator belongs. When the chief principal investigator receives information about a serious adverse event that does not fall under the above-mentioned illnesses, he/she will promptly report it to the hospital director and the Saga University certified review board.

The “Procedures for Emergency Reporting of Diseases” provides detailed procedures and reporting deadlines separately.

# **Compliance with the research plan and management of non-compliance**

## Compliance with the research plan

The principal investigator and co-investigators will conduct this study in accordance with the research plan.

Non-compliance refers to a state (deviation) in which the research does not comply with the Clinical Research Act, ministerial ordinance, or the research plan. Specifically, this refers to non-compliance with enforcement of regulations, implementation plans, research plans, procedure manuals, and falsification or fabrication of research data. Serious non-compliance refers to anything that affects the human rights and safety of the study participants, research progress, or reliability of the results, including non-compliance with inclusion/exclusion criteria, discontinuation criteria, or prohibited concomitant therapies, and does not include non-compliance with the research plan for unavoidable medical reasons, such as to avoid immediate danger to the study participants.

The principal investigator and co-investigators will not make any deviations without the prior approval of the certified review board. However, in cases of medical necessity, such as to avoid an immediate risk to study participants, the principal investigator and co-investigators may have to deviate from the research plan without the prior approval of the certified review board.

## Reporting and recording non-conformances

When a co-investigator becomes aware of a clinical research non-conformance, he/she shall promptly report this to the principal investigator. When a principal investigator becomes aware of a clinical research non-conformance, he/she shall promptly report this to the administrator of the medical institution conducting the research and notify the chief principal investigator. Furthermore, when a particularly serious non-conformance is discovered, the chief principal investigator shall promptly seek the opinion of the certified review board. Furthermore, when the chief principal investigator becomes aware of a non-conformance, he/she shall promptly inform the other principal investigators.

The principal investigator and co-investigators will record all deviations from the research plan, regardless of the reason.

## Obtaining permission to conduct the study at the medical institution

The chief principal investigator will listen to the opinion of the certified review board and obtain approval from the administrators of each participating medical institution regarding whether the study can be carried out at the particular institution, before submitting the implementation plan to the Minister of Health, Labour and Welfare.

# **Statistical analysis**

## Sample size

100 cases (Finerenone group, 50 cases; placebo group, 50 cases)

Rationale for sample size determination

During the research plan formulation, the clinical evidence on the effects of finerenone on surrogate markers, including vascular stiffness and biomarkers in patients with CKD complicated by type 2 diabetes, was limited. Therefore, the study sample size was set based on the results of two clinical trials: one that examined the effects of a drug of the same type as finerenone on these surrogate markers and another that examined the effects of a drug of a different type on surrogate markers similar to those in this study.

　First, a clinical trial using eplerenone, a different MRA, reported in 2021 (MIRAD: Mineralocorticoid Receptor Antagonists in Type 2 Diabetes) (1) revealed that administration of eplerenone for 26 weeks significantly improved the levels of surrogate markers associated with heart disease in patients with type 2 diabetes compared to that by placebo. The total number of participants analyzed in the study was 104, and although the participants and endpoints were different from those in this study, the effectiveness of MRA on surrogate markers related to cardiac and renal disease may be verified with a similar number of cases.

　Next, in a subanalysis of the EMPA-TROPISM trial assessing patients with HFrEF (2), after six months of administration of the SGLT2 inhibitor empagliflozin, the change in vascular stiffness assessed by aortic pulse wave velocity was –0.58 cm/s (–0.36 SD) in the empagliflozin group and +0.60 cm/s (+0.60 SD) in the placebo group, demonstrating a significant improvement with empagliflozin (estimated effect size is approximately –0.36 SD–0.60 SD = –0.96 SD), and at the same time, proteomic analysis revealed a reduction in some inflammatory biomarkers. Moreover, in the PROTECT trial (jRCT1071220089) assessing patients with type 2 diabetes, although not limited to patients with CKD, the average CAVI value was approximately 9 with an SD of approximately 1.5 (unpublished data). Using these values, –0.6SD approximately yields a CAVI of –1; thus, in this study, the CAVI value of approximately 9 is expected to lower by approximately 1. In studies focusing on patients with diabetes and the general population (3,4), the CAVI value increased by 0.5 with approximately 10 years of aging; thus, lowering the CAVI by 1 point is equivalent to delaying the progression of arterial stiffness by approximately 20 years, and is considered to be clinically significant.

　Although no studies have directly compared the clinical effects of finerenone and empagliflozin, based on previous studies, if we assume that finerenone will improve vascular stiffness similar to that by empagliflozin as observed in the EMPA-TROPISM trial, we conservatively estimated that the CAVI in SD units from baseline to 24 weeks after treatment initiation would be reduced by > –0.6 (63% of the effect detected in the EMPA-TROPISM trial, calculated as –0.96 × 0.63 = –0.6) in the finerenone group compared to that in the placebo group, and set the significance level at 5% on both sides, power at 80%, and dropout rate at 10%. Thus, the minimum number of cases required to detect a significant difference between the two groups was set at 100 (50 in each group).

References:

1. Brandt-Jacobsen NH, Lav Madsen P, Johansen ML, Rasmussen JJ, Forman JL, Holm MR, Rye Jørgensen N, Faber J, Rossignol P, Schou M, Kistorp C. Mineralocorticoid receptor antagonist improves cardiac structure in type 2 diabetes: data from the MIRAD trial. JACC Heart Fail. 2021;9(8):550–558.
2. Requena-Ibáñez JA, Santos-Gallego CG, Rodriguez-Cordero A, Vargas-Delgado AP, Mancini D, Sartori S, Atallah-Lajam F, Giannarelli C, Macaluso F, Lala A, Sanz J, Fuster V, Badimon JJ. Mechanistic insights of empagliflozin in nondiabetic patients with HFrEF: from the EMPA-TROPISM study. JACC Heart Fail. 2021;9(8):578–589.
3. Chung SL, Yang CC, Chen CC, Hsu YC, Lei MH. Coronary artery calcium score compared with cardio-ankle vascular index in the prediction of cardiovascular events in asymptomatic patients with type 2 diabetes. J Atheroscler Thromb. 2015;22(12):1255–1265.
4. Wohlfahrt P, Cífková R, Movsisyan N, Kunzová Š, Lešovský J, Homolka M, Soška V, Dobšák P, Lopez-Jimenez F, Sochor O. Reference values of cardio-ankle vascular index in a random sample of a white population. J Hypertens. 2017;35(11):2238–2244.

# Analysis set

# Efficacy analysis set

# Full analysis set (FAS)

In accordance with the intention-to-treat principle, the FAS, excluding the following cases, will be used for efficacy analysis:

- - Patients who withdrew consent after enrollment
  - Patients identified to be ineligible after enrollment
  - Patients not administered protocol treatment after allocation
  - Patients with no data on efficacy after protocol treatment initiation

## Per-protocol set (PPS)

Among the FAS, the patient population without any significant deviations from the study plan will be designated as the PPS and will be used for supplementary efficacy analysis.

The data center will evaluate deviations from the study plan as cases accumulate, and before the study data is finalized, a case review meeting will be held with the principal investigator, the head of statistical analysis, the data center, and monitoring staff to define and extract cases of significant deviations from the study plan.

### Safety set (SS)

Randomized patients administered at least one dose of protocol treatment will be included in the safety set (SS).

## Analysis method

For assessing efficacy, FAS will be used as the primary analysis population with supplementary analysis of the PPS for sensitivity analysis. The significance level for all statistical tests will be two-sided at 0.05. No adjustment for multiplicity of tests will be conducted except for proteomics analysis. For details of the analysis not described in this research plan and abnormal data including outliers, the handling method will be considered before conducting the statistical analysis, and a statistical analysis plan will be created and established before the data is finalized. If any changes to the statistical analysis plan results in a consequent change in the research plan, the research and statistical analysis plans will be revised, and the details of the revisions will be explained in the overall report of the study.

## Case composition

A flow diagram will be created for cases with obtained consent, cases not enrolled, cases enrolled/allocated, cases discontinued, cases completed, and cases for which primary endpoints can be analyzed.

## Description of analysis set

The number of cases will be listed for each FAS, PPS, and SS. Excluded cases will be tabulated by the reasons for exclusion, and a list of excluded cases will be created.

## Patient background and baseline values

For patient characteristics in the FAS, summary statistics (number of cases, mean, standard deviation, minimum, 1st quartile [Q1: 25th percentile], median, 3rd quartile [Q3: 75th percentile], maximum) will be calculated for quantitative variables, and the number of cases and percentages (%) will be calculated for qualitative variables by treatment group. Furthermore, the number of cases and percentages (%) will be obtained for complications and use of prior medications by treatment group.

## Efficacy analysis

## Efficacy analysis

1. Analysis of the primary endpoint

The change in CAVI at 24 weeks after protocol treatment initiation compared to that at baseline will be analyzed using a linear regression model with the treatment group as a fixed effect factor and baseline CAVI value as a covariate. Moreover, the mean values of both treatment groups at 24 weeks and their 95% confidence intervals, as well as the treatment effect estimated by the difference in the mean values between the treatment groups and their 95% confidence intervals will be calculated. A test will be performed against the null hypothesis such that the difference in treatment effect is 0.

1. Analysis of secondary endpoints
2. Proportional change in geometric mean of UACR at 12 and 24 weeks after protocol treatment initiation compared to that at baseline (important secondary endpoint):

The change in logarithmic UACR values from baseline to 12 and 24 weeks after the protocol treatment initiation will be analyzed using mixed-effects models for repeated measures (MMRM) with the treatment group as a fixed effect factor, baseline value and time point as covariates, and group and time point interaction. The results will be back-transformed to the original UACR scale, and the proportional change in UACR geometric mean and its 95% confidence interval for both treatment groups at 12 and 24 weeks, as well as the treatment effect estimated by the ratio of the proportional change between the treatment groups and its 95% confidence interval, will be calculated. A test will be performed against the null hypothesis such that the treatment effect ratio is 1 at each time point.

1. Proportional change in geometric mean of blood biomarkers (pentosidine) and urinary biomarkers (type IV collagen, α1-MG, β2-MG, NGAL, NAG, L-FABP creatinine ratio) at 24 weeks after the protocol treatment initiation as compared to those at baseline:

The change in logarithmic value of each blood biomarker from baseline to 24 weeks after the protocol treatment initiation will be analyzed using a linear regression model with the treatment group as a fixed effect factor and the baseline value as a covariate. The results will be back-transformed to the original scale, and the proportional change in the geometric mean of both treatment groups at 24 weeks and its 95% confidence interval, as well as the treatment effect estimated by the ratio of the proportional change between the treatment groups and its 95% confidence interval, will be calculated. A test will be performed against the null hypothesis such that the treatment effect ratio is 1.

1. Analysis of other endpoints
2. Changes from baseline in vital signs, including weight, BMI, eEV, blood pressure, pulse pressure, and pulse rate in the clinic, and blood pressure, pulse pressure, and pulse rate at home at 4, 12, and 24 weeks after protocol treatment initiation:

The change from baseline in each outcome at each time point after the protocol treatment will be analyzed using MMRM with the treatment group as a fixed effect factor, baseline value and time point as covariates, and group and time point interaction, to calculate the mean values of both treatment groups at each time point and their 95% confidence intervals, as well as the treatment effect estimated by the difference in the mean values between the treatment groups and their 95% confidence intervals. A test will be performed against the null hypothesis such that the treatment effect difference is 0.

1. Changes from baseline in blood test indices, including serum creatinine, eGFR, serum cystatin C*, serum potassium, HbA1c*, plasma aldosterone concentration**, plasma renin activity** at 4, 12, and 24 weeks after the protocol treatment initiation (*Evaluated only at baseline, and 12, and 24 weeks, **Evaluated only at baseline and 24 weeks):

The change from baseline in each outcome at each time point after protocol treatment initiation will be analyzed using a linear regression model with the treatment group as a fixed effect factor and the baseline value as a covariate, or MMRM with the treatment group as a fixed effect factor, the baseline value and time point as covariates, and the interaction between group and time point. The mean values of both treatment groups at each time point and their 95% confidence intervals, as well as the treatment effect estimated by the difference in the mean values between the treatment groups and their 95% confidence intervals, will be calculated. A test will be performed against the null hypothesis such that the difference in treatment effect is 0. If the distribution of values deviates significantly from a normal distribution, appropriate scale transformation will be performed as necessary.

1. Changes from baseline in AI and % mean arterial pressure at 24 weeks after protocol treatment initiation:

The change from baseline in each outcome at 24 weeks after the protocol treatment initiation will be analyzed using a linear regression model with the treatment group as a fixed effect factor and the baseline value as a covariate. Moreover, the mean values of both treatment groups at 24 weeks and their 95% confidence intervals, as well as the treatment effect estimated by the difference in the mean values between the treatment groups and their 95% confidence intervals will be calculated. A test will be performed against the null hypothesis such that the difference in treatment effect is 0. If the distribution of values deviates significantly from a normal distribution, appropriate scale conversion will be performed as appropriate.

1. Changes from baseline in cardiac function indices, including LVEF, septal e′, lateral e′, E, E/e′*, LVMI, LAD, and LAVI assessed by echocardiography 24 weeks after protocol treatment initiation, compared to those at baseline:

The change from baseline in each outcome at 24 weeks after the protocol treatment initiation will be analyzed using a linear regression model with the treatment group as a fixed effect factor and the baseline value as a covariate. Moreover, the mean values of both treatment groups at 24 weeks and their 95% confidence intervals, as well as the treatment effect estimated by the difference in the mean values between the treatment groups and their 95% confidence intervals will be calculated. A test will be performed against the null hypothesis such that the difference in treatment effect is 0. If the distribution of values deviates significantly from a normal distribution, appropriate scale conversion will be performed.

1. Changes in abundance of up to 181 proteins comprehensively analyzed by proteomic analysis (Target 96 panel) at 24 weeks after protocol treatment initiation as compared to that at baseline (exploratory endpoint):

Comprehensive comparisons between groups will be performed for each evaluation item using *t*-tests. The Benjamini–Hochberg method will be used to control the false discovery rate to 5% under multiple comparisons.

### Safety analysis

Reported adverse events will be assigned a low-level term (LLT) code using the Medical Dictionary for Regulatory Affairs, Japanese (MedDRA/J). The version of MedDRA/J used for the analysis will be the latest version at the time of database fixation.

Adverse events (TEAEs) observed after protocol treatment initiation will be assessed. Adverse events identified to be causally related to the study drug will be considered side effects.

If an adverse event with the same preferred term (PT) occurred multiple times in one case, the number of occurrences will be considered as the number of events, and the number of cases will be counted separately as one case.

For all adverse events and side effects, the number of occurrences, number of cases, and incidence rate with 95% confidence intervals will be calculated for each treatment group.

The number of events, number of cases, and incidence rate will be determined by system organ class (SOC) and PT classification for each treatment group.

# **Interim analysis**

Not implemented.

# **Access to source documents**

During monitoring related to this study and investigations by the certified review board and regulatory authorities, the principal investigator and the medical institution conducting the study will provide direct access to all relevant records, including the source documents.

# **Quality control and quality assurance**

## Monitoring method

In this study, monitoring will be carried out in accordance with the “monitoring procedures,” prepared separately. Those engaged in monitoring (monitors) will follow the monitoring procedures and check the following items throughout the study period. Monitors must not leak any information learnt in the course of their work.

1. The human rights and safety of the participants in this study will be protected.
2. The study is being conducted in compliance with the current research plan, implementation plan, and regulatory requirements.
3. The accuracy of the CRF can be verified against source documents.

# **Ethical considerations**

This study will be conducted in accordance with the ethical principles based on the Declaration of Helsinki and in compliance with the Clinical Research Act (Act No. 16 of 2017), the Enforcement Regulations of the same Act (Ministry of Health, Labour and Welfare Ordinance No. 47 of April 1, 2022), and related notifications.

## Benefits, burdens, and anticipated disadvantages to study participants

Finerenone used in this study is manufactured and delivered by a different route than the over-the-counter drug finerenone; however, the same efficacy is expected for finerenone and the over-the-counter drug. Furthermore, the study drug is used in the same dosage and administration as the over-the-counter drug; hence, little risk beyond everyday clinical practice is expected. However, the same side effects as the over-the-counter drug finerenone may be observed. Furthermore, although a placebo is not expected to have a therapeutic effect, finerenone is considered an additional therapeutic drug for protecting the heart and kidneys in overseas guidelines, and in principle, the use of other therapeutic drugs is not prohibited. Moreover, as this study has a relatively short observation period of 24 weeks, minimizing the disadvantages by appropriate treatment is possible. However, even if the placebo group does not induce any therapeutic benefits, no compensation will be provided.

Moreover, owing to the centralized testing of blood biomarkers and proteomics performed in this study, the amount of blood drawn will increase by approximately 5 mL per collection compared to that during regular medical care. Furthermore, owing to the observations and tests specified in this study, the length of stay at the medical institution may be extended.

If the results of this study confirm the safety and effectiveness of finerenone for patients with CKD and type 2 diabetes and prove that finerenone is a useful treatment, this study could contribute to future medical advances.

In principle, this study will be conducted within the scope of regular insured medical care; therefore, the participants’ health insurance will cover the observations, tests, and medications used during the study period. However, some tests including blood biomarkers, urinary biomarkers, proteomics, performed at baseline and 24 weeks will not be covered by health insurance; therefore, the costs of these tests will be covered by research funds. Moreover, during participation in this study, in addition to the blood samples collected during regular medical care, additional blood samples will be collected for blood biomarker and proteomics analysis, and approximately 5 mL of additional blood will be collected at baseline and 24 weeks.

Possible side effects from participating in this study and being administered the study drug are described in “4.6.9 Side effects.”

In case of an adverse event (including side effects), the principal investigator or co-investigator will promptly conduct an appropriate medical examination and take appropriate measures. Moreover, to minimize the disadvantages caused by adverse events, the principal investigator or co-investigator will collect information necessary to safely conduct this study and take appropriate measures, including modifying the research plan as necessary.

## Handling of personal information

The principal investigator and co-investigators will consider protecting the personal information of the study participants during creation and handling CRFs. Each study participant will be identified by a patient identification number. The principal investigator and co-investigators will create an anonymized correspondence table to identify individual study participants, appropriately stored and managed within the medical institution conducting the research and not provided outside the medical institution conducting the research.

When publishing the results of this study, we will ensure that the patient information is anonymized.

If a study participant requests disclosure of personal information that can identify that individual, the principal investigator will disclose the relevant personal information to the requesting individual without delay. The principal investigator will also disclose the research plan and details of the research.

# **Handling and storage of records (including data)**

The chief principal investigator must properly preserve the information listed below and the related records pertaining to this study until at least five years after the end of the study. Samples collected and measured at each participating medical institution will be properly disposed of at each participating medical institution.

1. Documents to identify the study participants (anonymization correspondence table)
2. Medical and examination records of the study participants
3. Informed consent forms of the participants (signed original copy)
4. Paper records or data regarding the central testing results sent to the chief principal investigator from the testing institutions designated by SRL, Inc. and the Department of Cardiology, Saga University Hospital
5. Research plan and informed consent form for this study
6. Documents, including implementation plans, submitted to the certified review board
7. Notification of results from the certified review board
8. Documents pertaining to monitoring
9. Contracts concluded regarding the conduct of this study (excluding contracts concluded with funders)
10. Other documents and records related to this study

Data used in the study will be provided from the medical institution conducting the research to the data center via EDC. The research office, monitoring staff, and statistical analysis staff will be permitted to access the collected data stored at the data center based on specific access rights, including EDC user IDs and passwords. The data center will properly store and manage the provided information until the data has been delivered to the principal investigator.

After the retention period ends, paper documents will be shredded and destroyed. Other information will be anonymized and disposed of in an appropriate manner.

# **Matters concerning payment and compensation**

## Financial burden associated with the research

The costs of the study treatment and any tests or examinations performed in the study will be borne by the study participant, as would be the case for regular medical care, except for the study drug, which will be covered by the research fund.

In principle, this study will be conducted within the scope of regular insured medical care; therefore, the participants’ health insurance will cover observations, examinations, and medications used during the study, and no medications or tests outside insurance coverage will be administered. However, some tests (blood biomarkers, urinary biomarkers, proteomics) conducted at baseline, 24 weeks, and at discontinuation will not be covered by health insurance; hence, the costs of these tests will be covered by research funds. Participants will be provided 20,000 yen in total with a QUO card or other payment to reduce their burden, with 10,000 yen at baseline and 10,000 yen at 24 weeks.

## Compensation and damages for health damage caused by the research

In the event of any health damage resulting from participation in this study, the principal investigator and co-investigator will provide the study participant with appropriate treatment and procedures. In such cases, treatment will be covered by insurance, and the study participant will pay their own share of the medical expenses. In addition, because the treatment in this study is covered by the Pharmaceutical Adverse Reaction Relief System, this system may be applied in the event of health damage. However, to cover liability for serious health damage (class 1 disability, class 2 disability, death), medical expenses, and medical treatment (coverage for unknown and known side effects), the representative principal investigator will take out liability insurance under the clinical research insurance. Treatment for any other health damage will be provided using the study participant’s health insurance. In addition, in the event of health damage resulting from the test drug used in this study, it will be covered by product liability insurance.

## Clinical research insurance coverage

1. Compensation (death and disability compensation)

(1) If the study participant is a patient (per study participant)

(The disability rating will be in accordance with the rating set by the Drug Adverse Reaction Relief and Research Promotion Organization.)

| Insurance claim category | Payment type | Compensation limit per study participant | | Compensation limit per accident / clinical research |
| --- | --- | --- | --- | --- |
| Death benefit | If the study participant is the main breadwinner | 20 million yen | | 100 million yen |
|  | If the study participant is not the main breadwinner | 7 million yen | |  |
| Permanent disability compensation insurance | If the study participant is the main breadwinner | Residual disability grade 1 | 30 million yen |  |
|  |  | Residual disability grade 2 | 24 million yen |  |
|  | If the study participant is not the main breadwinner | Residual disability grade 1 | 20 million yen |  |
|  |  | Residual disability grade 2 | 16 million yen |  |

2. Medical expenses compensation and medical benefits compensation

|  | Insurance amount and compensation limit | | | | Deductible amount | Payment deadline  Number of months |
| --- | --- | --- | --- | --- | --- | --- |
|  | Collateral type | Per victim | Per accident | During the period | Per accident |  |
| Additional provisions for medical expenses and medical benefits compensation (medical expenses) | Physical disability | 1 million yen | 10 million yen | 10 million yen | 0 yen | 12 months |
| Additional provisions for medical expenses and medical benefits compensation  (medical benefits) | Physical disability | 37,000 yen per month | 10 million yen | 10 million yen | 0 yen | 12 months |

# **Publication of research information**

## Research plan registration

The chief principal investigator will register an outline of the study in the Japan Registry of Clinical Trials (jRCT) and ClinicalTrials.gov prior to its implementation, and will update it accordingly according to changes in the implementation plan and progress of the study. When the study is completed, the results of the study will be registered without delay.

## Publication of results

When publishing the results, necessary measures will be taken to protect the human rights of the study participants and their associates, as well as the rights and interests of those involved in the research and their associates.

The chief principal investigator shall prepare the primary endpoint report, as well as the comprehensive report and its summary within the timeline provided below. Once prepared, the chief principal investigator shall seek the opinion of the certified review board mentioned in the implementation plan, and then promptly submit them to the administrator of the medical institution conducting the study and register the summary in the jRCT. If the primary endpoint report and comprehensive report are prepared at the same time, the preparation of the comprehensive report will also be considered as the preparation of the primary endpoint report.

1) Primary endpoint report

In principle, within one year after the end of the data collection period associated with the primary endpoint

2) Comprehensive report and its summary

In principle, within one year after the end of the data collection period associated with all the endpoints

# **Research period**

## Research period

Case registration period: Japan Registry of Clinical Trials (jRCT) release date to July 31, 2024

Observation period: 24 weeks

Research implementation period: Japan Registry of Clinical Trials (jRCT) release date to July 31, 2026

## End of study

This study will end when the results are published in jRCT, a database maintained by the Ministry of Health, Labour and Welfare. The chief principal investigator will provide the results of the publication to the principal investigators of each participating medical institution. The principal investigator will report the information provided by the chief principal investigator to the administrator of each participating medical institution.

# **Explaining the study to the participants and collecting their informed consent**

## Procedures for obtaining informed consent

- 1. Prior to the patient’s participation in the study, the principal investigator or co-investigator will provide the patient an informed consent form approved by the certified review board, and thorough verbal explanation of the information presented in “16.2 Explaining the study to the participants.” After providing the patient sufficient time to decide if they want to participate in the study and confirming that the patient fully understands the contents, the patient will provide their voluntary, written consent to participate in the study.
  2. The informed consent document must be signed and dated by the principal investigator or co-investigator, providing the explanation to the patient. If a research collaborator provides supplementary explanation, the research collaborator must also sign and date the document.
  3. The original informed consent form with the name and seal or signature and date will be stored by the principal investigator or co-investigator in accordance with the regulations of the medical institution conducting the research. A copy of the informed consent form will be provided to the patient.
  4. When information that may affect the consent of the study participant is obtained or when changes are incorporated in to the research plan that may affect the consent of the study participant, the principal investigator or co-investigator will promptly communicate that information to the study participants. If a method is specified by the medical institution conducting the research, records will be maintained in accordance with that method. Moreover, with the prior approval of the certified review board, the informed consent form will be revised, and a new consent will be obtained in writing from the study participants.

## Explaining the study to the participants

The informed consent form shall include the following items:

1. The name of the specific clinical research to be conducted, a statement indicating that approval has been obtained from the administrator of the medical institution conducting the specific clinical research, and that an implementation plan has been submitted to the Minister of Health, Labour and Welfare
2. The name of the institution conducting the clinical study and the name and job title of the principal investigator (if the specific clinical study is conducted as a multi-institutional joint research, the name and job title of the chief principal investigator, the names of other conducting medical institutions, and the names and job titles of the principal investigators of those medical institutions are included).
3. Reasons for being selected as a participant for the specific clinical study
4. The anticipated benefits and drawbacks of conducting the specific clinical study
5. A statement indicating that the participants can refuse to participate in a specific clinical study
6. Information on withdrawal of consent
7. A statement indicating that the patient will not be subjected to any disadvantageous treatment for refusing to participate in the specific clinical study or for withdrawing his/her consent
8. Method of disclosing information regarding specific clinical studies
9. A statement indicating that the research plan and other materials related to the implementation of the specific clinical study can be obtained or viewed at the request of the participant of the specific clinical study or their legal representative, and the method for obtaining or viewing such materials
10. Information on the protection of personal information of the participants of the specific clinical study
11. Information on storage and disposal of samples
12. Status of the provision of research funds and other involvement in the specific clinical study
13. System for handling complaints and inquiries
14. Information on the costs associated with the implementation of the specific clinical study
15. Availability and content of other treatments and comparison of the benefits and harms expected compared to other treatment options
16. Information on compensation and provision of medical care for health damage caused by the specific clinical study
17. Information on details reviewed by the certified review board that provides review and opinion services for this specific clinical study, and other topics related to the certified review board for this specific clinical study
18. Other topics necessary for the implementation of the specific clinical study

## Revision of the informed consent form

If the chief principal investigator receives information that indicates the need to revise the informed consent form, he/she shall promptly revise the informed consent form based on that information. When the chief principal investigator revises the informed consent form, he/she shall submit it to the certified review board specified in the implementation plan and obtain approval. After that, each principal investigator shall obtain approval from the administrator of the medical institution conducting the research.

## Withdrawal of consent

If a study participant requests to withdraw their consent, the principal investigator or co-investigator will use the consent withdrawal form to confirm the details of the study participant’s withdrawal of consent. The study participant will check the appropriate item regarding the details of consent withdrawal on the consent withdrawal form, record the date of consent withdrawal, and sign. The principal investigator or co-investigator will confirm the contents of the consent withdrawal form, record the date of confirmation, and sign. The principal investigator or co-investigator will provide a copy of the signed consent withdrawal form to the study participant and store the original at the medical institution.

## The possibility of the samples and information obtained from the study participants being used for future research

The remaining samples after baseline and 24-week blood and urinary biomarker measurements will be stored for a certain period after completion of the study, and then discarded, in accordance with the procedures of SRL, Inc. Moreover, the remaining samples after proteomics analysis will be discarded by the Department of Cardiology, Saga University Hospital after the completion of the study.

　If the participant providing consent withdraws the consent after consent has been obtained for the storage and use of samples and information after the study is conducted, the samples and information related to the study will be immediately disposed of in accordance with the participant’s wishes.

　Furthermore, if the samples and information obtained through this study are to be used in a newly planned study in the future, a new research plan will be formulated and approved by an appropriate ethical review committee depending on the content of the research, and then the data will be provided to the researcher in a form that does not identify the individual study participants.

# **Research funding and conflicts of interest**

## Funding sources and financial relationships associated with the clinical trial

This research will be conducted with funding from Bayer Yakuhin, Ltd. based on an investigator-initiated clinical research agreement with Saga University. Saga University will be responsible for conducting the research, and will enter into a contract with a contracted development organization to provide support services associated with monitoring, data management, statistical analysis, and coordination management in accordance with the contract. Bayer Yakuhin, Ltd. will provide information about finerenone, and offer information and opinions on research plans and summary reports; however, it will not be involved in the implementation, analysis, interpretation, or reporting of the research results. Furthermore, unless there is a legitimate reason, it will not refuse to publish the results of the research.

## Attribution of results

All patents and other results obtained from this study will belong to Bayer Yakuhin Ltd., and the chief principal investigator and the medical institution to which the chief principal investigator is affiliated will have non-exclusive, free, and unrestricted use of the results.

## Management of conflicts of interest

The chief principal investigator shall prepare conflict of interest management standards for the clinical research to be conducted and notify the principal investigators. Based on the conflict of interest status identified via the conflict of interest confirmation results (researcher conflict of interest self-declaration form) and the contents of the report from the administrator of the medical institution conducting the research or the head of the affiliated institution (conflict of interest status confirmation report), the principal investigator shall prepare a conflict of interest management plan for each institution and submit it to the chief principal investigator. The chief principal investigator shall seek the opinion of the certified review board regarding the conflicts of interest management standards and conflict of interest management plan (including cases with changes). Conflicts of interest shall be managed appropriately in accordance with the approved conflict of interest management standards and conflict of interest management plan.

## Conflict of interest status

The chief principal investigator receives personal income of > 1 million yen per year from Bayer Yakuhin, Ltd.; however, this will not result in undue influence from the company on the results of this study. Moreover, a principal investigator receiving a personal income of > 1 million yen per year from Bayer Yakuhin Co., Ltd. Is involved in the study; however, this will not result in undue influence from the company on the results of this study.

# **Certified review board**

## Certified review board

This study will be reviewed and conducted by the following certified review board.

Name: Clinical Research Review Board, Fukushima Medical University (Certification number: CRB2200002)

Address: 1 Hikarigaoka, Fukushima City, Fukushima Prefecture

Contact: fmucrb@fmu.ac.jp

TEL: 024-547-1825 (Clinical Research Review Board Secretariat)

## Reporting to certified review boards

## Regular reports

1. Periodic reporting to the clinical research review board

The chief principal investigator will report the status of this study to the certified review board using the items below annually from the date of submission of the implementation plan to the Minister of Health, Labour and Welfare, and within two months after the end of that period. Furthermore, when making regular reports, reports will also be provided to the administrator of the medical institution conducting the research.

- 1. Number of study participants enrolled
  2. Incidence of diseases and their subsequent progress
  3. Circumstances of non-conformity and subsequent responses
  4. Safety and scientific validity evaluation
  5. Data on the involvement of pharmaceutical manufacturers and distributors as stipulated in the conflicts of interest management standards

1. Regular reporting to the Minister of Health, Labour and Welfare

The chief principal investigator will provide the items mentioned below to the Minister of Health, Labour and Welfare within one month from the date on which the certified review board stated in the implementation plan issues its opinion on the status of the research.

- 1. The name of the certified review board listed in the implementation plan
  2. Determination of suitability to continue the study by the certified review board
  3. Number of study participants enrolled
  4. Incidence of diseases and their subsequent progress
  5. Circumstances of non-conformity and subsequent responses
  6. Safety and scientific validity evaluation
  7. Information on the involvement of pharmaceutical manufacturers and distributors as stipulated in the conflicts of interest management standards

## Other reports

During the study, the chief principal investigator must report to the certified review board:

1. On identification of issues including an outbreak of a disease.
2. On discovery of a major non-conformity
3. On preparation of the primary endpoint report or clinical trial report and its summary

# **Changes to the research plan**

If the research plan needs to be revised after the study initiation, the chief principal investigator will prepare a document outlining the revised plan and its revision history (the contents of the revisions and the reasons for them) to accommodate possible changes to the implementation plan. Then, the chief principal investigator will seek the opinion of the certified review board.

However, this does not include minor changes that fall under the conditions specified below.

1) A change in the name of a person engaged in clinical research that does not involve a change in the person engaged in the clinical research (a name change for reasons including change in marital status)

2) Changes owing to changes in the name of the area or the address (the location remains the same)

　After receiving a report on the revisions to the research plan from the chief principal investigator, the principal investigators shall submit them to the review organization of the medical institution conducting the study, if necessary (changes that are deemed minor by the medical institution may be approved as items to be reported).

If suspension of case registration is necessary during the revision, the chief principal investigator will notify the principal investigators and co-investigators, data center, and persons responsible for statistical analysis of that effect. After the revision, the chief principal investigator will send the revised research plan or the details of the revisions to the principal investigators and co-investigators, data center, and persons responsible for statistical analysis. The chief principal investigator will also revise the informed consent forms for the study participants based on the details of the revisions to the research plan.

If, during the course of this study, a particular medical institution decides to discontinue the study, the chief principal investigator will submit a change to the implementation plan after the observation period for the study participants at that medical institution has ended.

# **Content and method of reporting to the administrator of the medical institution**

1. If the principal investigator obtains facts or information that undermines or is likely to undermine the ethical appropriateness or scientific rationality of the research, he/she must promptly report this to the administrator of the medical institution conducting the research and, if necessary, suspend or discontinue the research or modify the research plan.
2. If the principal investigator obtains facts or information that undermines or is likely to undermine the proper conduct of the research or reliability of the research results, he/she must promptly report this to the administrator of the medical institution conducting the research and, if necessary, suspend or terminate the research or modify the research plan.
3. If the principal investigator becomes aware of a clinical study not being in compliance with ministerial ordinances or the research plan (non-compliance), he/she must promptly report this to the administrator of the medical institution conducting the study and notify the chief principal investigator of the same.
4. The principal investigator must report the progress of the research and occurrence of any illnesses or other issues related to the conduct of the research to the administrator of the medical institution conducting the research, as specified in the research plan (in principle, once a year).
5. When the principal investigator completes or discontinues the study, he/she must promptly report this fact and a summary of the results of the study to the administrator of the medical institution conducting the study in writing.
6. If the principal investigator becomes aware of a serious illness or infectious disease that could not have been predicted based on the precautions for using the investigational drug and is suspected to have been caused by the implementation of this study, he/she must report this to the administrator of the medical institution conducting the study and then notify the chief principal investigator. The chief principal investigator must promptly provide this information to the other principal investigators.
7. When the chief principal investigator receives an opinion from the certified review board, he/she must promptly report this opinion to the administrator of the medical institution conducting the study and provide this information to the other principal investigators. The other principal investigators who receive information from the chief principal investigator must promptly report this information to the administrator of the medical institution conducting the study.
8. When the chief principal investigator prepares the primary endpoint report or clinical trial report and its summary, he/she must, after first consulting the certified review board, submit it to the administrator of the medical institution without delay and make the summary of the primary endpoint report or clinical trial report public. When the chief principal investigator submits the summary of the clinical trial report, he/she must submit the summary of the clinical trial report, research plan, and statistical analysis plan to the Minister of Health, Labour and Welfare, promptly report this to the administrator of the medical institution, and provide information regarding this to the other principal investigators. In this case, the other principal investigators must promptly report the provided information to the administrator of the medical institution conducting the study.
9. When the research is completed, the principal investigator must take the necessary measures to protect the human rights of the study participants and those related to them, and the rights and interests of the researchers and those related to them, without delay, and then publish the results of the research. Moreover, on final publication of the results, the principal investigator must report it to the administrator of the medical institution conducting the study without delay.

# **Provision of medical care to study participants after the study is completed**

No regulations will be imposed regarding treatment after the end of this study. The principal investigator and co-investigators will ensure that study participants receive the best possible prevention, diagnosis, and treatment even after the study ends.

# **Study protocol amendment**

Created on June 30, 2023

Research Plan Revision Comparison Table 1 (ver1.1 to 1.2)

| **(Before change) April 12, 2023** | **(After change) June 30, 2023** | **Reason for change** |
| --- | --- | --- |
| April 12, 2023 ver1.1 | June 30, 2023 ver1.2 | Revision |
| Creation date: April 12, 2023  Version: 1.1 | Creation date: June 30, 2023  Version: 1.2 | Revision |
| **Revision history**   \| Version \| Creation and revision dates \| Reasons for revision \| Scheduled implementation date \| \| --- \| --- \| --- \| --- \| \| Version 1.0 (Draft Edition) \| 2023/1/27 \| Newly created \| - \| \| Version 1.1 \| 2023/4/12 \| For approval by the Certified Review Board \| 2023/5/1 \| | **Revision history**   \| Version \| Creation and revision dates \| Reasons for revision \| Scheduled implementation date \| \| --- \| --- \| --- \| --- \| \| Version 1.0 (Draft Edition) \| 2023/1/27 \| Newly created \| - \| \| Version 1.1 \| 2023/4/12 \| For approval by the Certified Review Board \| 2023/5/1 \| \| Version 1.2 \| 2023/6/30 \| correction \| 2023/7/1 \| | Revision |
| **Research summary**  Endpoints  Other endpoints  2. Changes from baseline in blood test indices (serum creatinine, eGFR, serum cystatin C*, serum potassium, HbA1c*, plasma aldosterone concentration**, plasma renin activity**) at 4, 12, and 24 weeks after the protocol treatment initiation (*evaluated only at baseline, and 12, and 24 weeks; **evaluated only at baseline and 24 weeks) | **Research summary**  Endpoints  Other endpoints  2. Changes from baseline in blood test indices (serum creatinine, eGFR, serum cystatin C*, serum potassium, HbA1c*, plasma (or serum is also acceptable) aldosterone concentration**, plasma (or serum is also acceptable) renin activity or concentration**) at 4, 12, and 24 weeks after the protocol treatment initiation (*evaluated only at baseline, and 12, and 24 weeks; **evaluated only at baseline and 24 weeks) | Added information |
| **Research summary**  Target number of cases  100 cases (Finerenone group: 50 cases, Placebo group: 50 cases) | **Research summary**  Target number of cases  100 cases (Finerenone group: 50 cases, Placebo group: 50 cases)  However, for target patients with consent already obtained when the target number of cases is reached, registration may be possible even after the target number of cases has been reached. | Description adjustment for the protection of eligible patients with consent already obtained when the target number of cases is reached. |
| **2. Research contents**  **2.1. Endpoints**  3) Other endpoints:  2. Changes from baseline in blood test indices (serum creatinine, eGFR, serum cystatin C*, serum potassium, HbA1c*, plasma aldosterone concentration**, plasma renin activity**) at 4, 12, and 24 weeks after the protocol treatment initiation (*evaluated only at baseline, and 12, and 24 weeks; **evaluated only at baseline and 24 weeks) | **2. Research contents**  **2.1. Endpoints**  3) Other endpoints:  2. Changes from baseline in blood test indices (serum creatinine, eGFR, serum cystatin C*, serum potassium, HbA1c*, plasma (or serum is also acceptable) aldosterone concentration**, plasma (or serum is also acceptable) renin activity or concentration**) at 4, 12, and 24 weeks after the protocol treatment initiation (*evaluated only at baseline, and 12, and 24 weeks; **evaluated only at baseline and 24 weeks) | Added information |
| **2. Research contents**  **2.5. Regulations for handling study drugs (management and distribution procedures)**  **2.5.1. Centralized management of study drugs**  Both the active and placebo test drugs will be delivered to the Department of Cardiology at Saga University Hospital by Bayer Yakuhin, Ltd., where they are temporarily stored. Each bottle is assigned a different number and a correspondence table of active drug and placebo is delivered at the same time. After delivery, the Study Drug Allocation Officer will pack for each case individually while checking the correspondence table. | **2. Research contents**  **2.5. Regulations for handling study drugs (management and distribution procedures)**  **2.5.1. Centralized management of study drugs**  Both the active and placebo test drugs will be delivered to the Department of Cardiology at Saga University Hospital by Bayer Yakuhin, Ltd. After being allocated (packaged) by the Study Drug Allocation Officer, they will be stored in the Pharmacy Department at Saga University Hospital. | Description adjustment |
| **2. Research contents**  **2.5. Regulations for handling study drugs (management and distribution procedures)**  **2.5.2. Delivery of study drugs to each medical institution**  The drugs will be delivered to each participating medical institution by an independent Study Drug Management Officer in the Pharmacy Department of the Saga University Hospital. | **2. Research contents**  **2.5. Regulations for handling study drugs (management and distribution procedures)**  **2.5.2. Delivery of study drugs to each medical institution**  The drugs will be delivered to each participating medical institution by an independent Study Drug Central Manager in the Pharmacy Department of the Saga University Hospital. | Description adjustment |
| **2. Research contents**  **2.8. Randomization procedure**  [Allocation factor]  eGFR (< 60 mL/min/1.73 m^2^ / 60 mL/min/1.73 m^2^ or >)* | **2. Research contents**  **2.8. Randomization procedure**  [Allocation factor]  eGFR (< than 45 mL/min/1.73 m^2^ / 45 mL/min/1.73 m^2^ or >)* | Typo |
| **4. Treatment of the study participants**  **4.1. Observation and examination schedule**  Table 1. Observation and examination schedule  7) For UACR and serum cystatin C, baseline data will be prioritized; however, if baseline data is unavailable, data within 3 months from the baseline may be used. | **4. Treatment of the study participants**  **4.1. Observation and examination schedule**  Table 1. Observation and examination schedule  7) For UACR and serum cystatin C, baseline data will be given prioritized; however, if baseline data is unavailable, data from within 3 months prior to the baseline may be used. | Correction |
| **4. Treatment of the study participants**  **4.1. Observation and examination schedule**  Table 1. Observation and examination schedule  11) Hemoglobin, hematocrit, serum albumin, serum potassium, HbA1c, plasma aldosterone concentration, plasma renin activity  15) Only serum potassium, HbA1c, plasma aldosterone concentration, and plasma renin activity will be measured. | **4. Treatment of the study participants**  **4.1. Observation and examination schedule**  Table 1. Observation and examination schedule  11) Hemoglobin, hematocrit, serum albumin, serum potassium, HbA1c, plasma (or serum is also acceptable) aldosterone concentration, plasma (or serum is also acceptable) renin activity or concentration  15) Only serum potassium, HbA1c, plasma (or serum is also acceptable) aldosterone concentration, and plasma (or serum is also acceptable) renin activity or concentration will be measured. | Added information |
| **4. Treatment of the study participants**  **4.2. Patient enrollment**  2) Based on the results of the eligibility assessment, the eligibility and allocation test items will be entered into the EDC, and registration and allocation will be performed. A patient identification number will be assigned to registered study participants (including those determined as "ineligible" by the EDC), and the group to which they were assigned will be displayed for eligible cases. | **4. Treatment of the study participants**  **4.2. Patient enrollment**  2) Based on the results of the eligibility assessment, the eligibility and allocation test items will be entered into the EDC, and registration and allocation will be performed. A patient identification number will be assigned to the registered study participants (including those determined as "ineligible" by the EDC), and a "study drug number" will also be displayed. | Typo |
| **4. Treatment of the study participants**  **4.2. Patient enrollment**  **4.2.2. Patient background information**  Duration of diabetes, presence or absence of complications/history (hypertension, dyslipidemia, ischemic heart disease, stroke, lower limb arteriosclerosis obliterans, heart failure), primary underlying disease of chronic renal failure (diabetic nephropathy, non-diabetic nephropathy: chronic glomerulonephritis, nephrosclerosis, polycystic kidney disease, collagen disease-related nephropathy, others) | **4. Treatment of the study participants**  **4.2. Patient enrollment**  **4.2.2. Patient background information**  Duration of diabetes, presence or absence of complications/history (hypertension, dyslipidemia, ischemic heart disease, stroke, lower limb arteriosclerosis obliterans, heart failure), primary underlying disease of chronic kidney disease (diabetic nephropathy, non-diabetic nephropathy: chronic glomerulonephritis, nephrosclerosis, polycystic kidney disease, collagen disease-related nephropathy, others) | Typo |
| **4. Treatment of the study participants**  **4.3. Observation and testing items for the prescribed visit**   1. Baseline (Day 0)   g. Blood test:  Hemoglobin, hematocrit, serum albumin, serum potassium, HbA1c***, plasma aldosterone concentration, plasma renin activity  h. Cardiac ultrasound examination (optional)****:  LVEF, septal e', lateral e', mitral orifice blood flow velocity waveform (E), E/e', LVMI, LAVI | **4. Treatment of the study participants**  **4.3. Observation and testing items for the prescribed visit**   1. Baseline (Day 0)   g. Blood test:  Hemoglobin, hematocrit, serum albumin, serum potassium, HbA1c***, plasma (or serum is also acceptable) aldosterone concentration, plasma (or serum is also acceptable) renin activity or concentration  h. Cardiac ultrasound examination (optional)****:  LVEF, septal e', lateral e', mitral orifice blood flow velocity waveform (E), E/e', LVMI, LAD, LAVI | Added information and typos |
| **4. Treatment of the study participants**  **4.3. Observation and testing items for the prescribed visit**   1. Baseline (Day 0)   **Baseline data will be prioritized; however, if baseline data is unavailable, data within 3 months from the baseline may be used. | **4. Treatment of the study participants**  **4.3. Observation and testing items for the prescribed visit**   1. Baseline (Day 0)   **Baseline data will be prioritized; however, if baseline data is unavailable, data from within 3 months prior to the baseline may be used. | For correction. |
| **4. Treatment of the study participants**  **4.3. Observation and testing items for the prescribed visit**  4) 24 weeks (Day 168, allowable range for observation and testing: Day 154-182)  g. Blood test:  Serum potassium, HbA1c, plasma aldosterone concentration, plasma renin activity  h. Cardiac ultrasound examination (optional):  LVEF, septal e', lateral e', mitral orifice blood flow velocity waveform (E), E/e', LVMI, LAVI  i. Concomitant medications:  ACE inhibitors, ARBs, β-blockers, calcium antagonists, diuretics, statins, insulin, metformin, SGLT2 inhibitors, GLP-1 receptor agonists, DPP-4 inhibitors, iron preparations, ESAs, HIF-PH inhibitors, and hyperkalemia medications | **4. Treatment of the study participants**  **4.3. Observation and testing items for the prescribed visit**  4) 24 weeks (Day 168, allowable range for observation and testing: Day 154-182)  g. Blood test:  Serum potassium, HbA1c, plasma (or serum is also acceptable) aldosterone concentration, and plasma (or serum) renin activity or concentration  h. Cardiac ultrasound examination (optional):  LVEF, septal e', lateral e', mitral orifice blood flow velocity waveform (E), E/e', LVMI, LAD, LAVI  i. Concomitant medications:  ACE inhibitors, ARBs, ARNIs, β-blockers, calcium antagonists, diuretics, statins, insulin, metformin, SGLT2 inhibitors, GLP-1 receptor agonists, DPP-4 inhibitors, iron preparations, ESAs, HIF-PH inhibitors, and hyperkalemia medications | Added information and typos |
| **4. Treatment of the study participants**  **4.7. Concomitant medications during the observation period**  In both groups, the physician in charge will make a comprehensive clinical judgment, including the condition of the study participants, and in principle there will be no restrictions on the use of drugs other than the study drug (drugs for type 2 diabetes, chronic renal failure, and other comorbid diseases); however, in principle no new administration of SGLT2 inhibitors will be implemented during the observation period. | **4. Treatment of the study participants**  **4.7. Concomitant medications during the observation period**  In both groups, the physician in charge will make a comprehensive clinical judgment, including the condition of the study participants, and in principle there will be no restrictions on the use of drugs other than the study drug (drugs for type 2 diabetes, chronic kidney disease, and other comorbid diseases); however, in principle no new administration of SGLT2 inhibitors will be implemented during the observation period. | Typo |
| **5. Efficacy and safety evaluation**  **5.3 Other endpoints**  2. Changes from baseline in blood test indices (serum creatinine, eGFR, serum cystatin C*, serum potassium, HbA1c*, plasma aldosterone concentration**, plasma renin activity**) at 4, 12, and 24 weeks after the protocol treatment initiation (*evaluated only at baseline, and 12, and 24 weeks; **evaluated only at baseline and 24 weeks) | **5. Efficacy and safety evaluation**  **5.3 Other endpoints**  2. Changes from baseline in blood test indices (serum creatinine, eGFR, serum cystatin C*, serum potassium, HbA1c*, plasma (or serum is also acceptable) aldosterone concentration**, plasma (or serum is also acceptable) renin activity or concentration**) at 4, 12, and 24 weeks after the protocol treatment initiation (*evaluated only at baseline, and 12, and 24 weeks; **evaluated only at baseline and 24 weeks) | Added information |
| **7. Statistical analysis**  **7.1. Target number of cases**  Total target number of cases: 100 cases (Finerenone group: 50 cases, placebo group: 50 cases)  [Basis for setting]  Based on the results of previous studies, although no studies have directly compared the clinical effects of Finerenone and empagliflozin. If Finerenone is assumed to possess the same effect in improving vascular stiffness as empagliflozin observed in the EMPA-TROPISM study, ... | **7. Statistical analysis**  **7.1. Target number of cases**  Total target number of cases: 100 cases (Finerenone group: 50 cases, placebo group: 50 cases)  However, for target patients with consent already obtained when the target number of cases is reached, registration may be possible even after the target number of cases has been reached.  [Basis for setting]  Based on the results of the above previous studies, although there is no data directly comparing the clinical effects of Finerenone and empagliflozin. If Finerenone is assumed to possess the same effect in improving vascular stiffness as empagliflozin observed in the EMPA-TROPISM study, ... | Description adjustment for the protection of eligible patients with consent already obtained when the number of cases is reached.  Added information and typos |
| **7. Statistical analysis**  **7.4. Efficacy analysis**  C) Analysis of other endpoints  b. Changes from baseline in blood test indices (serum creatinine, eGFR, serum cystatin C*, serum potassium, HbA1c*, plasma aldosterone concentration**, plasma renin activity**) at 4, 12, and 24 weeks after the protocol treatment initiation (*evaluated only at baseline, and 12, and 24 weeks, **evaluated only at baseline and 24 weeks) | **7. Statistical analysis**  **7.4. Efficacy analysis**  C) Analysis of other endpoints  b. Changes from baseline in blood test indices (serum creatinine, eGFR, serum cystatin C*, serum potassium, HbA1c*, plasma (or serum is also acceptable) aldosterone concentration**, plasma (or serum is also acceptable) renin activity or concentration**) at 4, 12, and 24 weeks after the protocol treatment initiation (*evaluated only at baseline, and 12, and 24 weeks, **evaluated only at baseline and 24 weeks) | Added information |
| **17. Research funding and conflicts of interest**  **17.4. Conflict of interest**  Moreover, a principal investigator receives a personal income > 1 million yen per year from Bayer Yakuhin Ltd.; however, this will not result in any undue influence from the company on the results of this study. | **17. Research funding and conflicts of interest**  **17.4. Conflict of interest**～  In addition, a principal investigator receives a personal income of > 1 million yen per year, and a co-investigator receives a personal income of > 2.5 million yen per year, from Bayer Yakuhin Co., Ltd.; however, this will not result in any undue influence from the company on the results of this study. | For correction in accordance with the latest conflict of interest status |

Created on October 20, 2023

Research Plan Revision Comparison Table 2 (ver1.2 to 1.3)

| (Before change) June 30, 2023 | (After change) October 20, 2023 | Reason for change |
| --- | --- | --- |
| June 30, 2023 ver1.2 | October 20, 2023 ver1.3 | Revision |
| Creation date: June 30, 2023  Version: 1.2 | Creation date: October 20, 2023  Version: 1.3 | Revision |
| **Revision history**   \| Version \| Creation and revision dates \| Reasons for revision \| Scheduled implementation date \| \| --- \| --- \| --- \| --- \| \| Version 1.0 (Draft Edition) \| 2023/1/27 \| Newly created \| - \| \| Version 1.1 \| 2023/4/12 \| For approval by the Certified Review Board \| 2023/5/1 \| \| Version 1.2 \| 2023/6/30 \| Correction \| 2023/7/1 \| | **Revision history**   \| Version \| Creation and revision dates \| Reasons for revision \| Scheduled implementation date \| \| --- \| --- \| --- \| --- \| \| Version 1.0 (Draft Edition) \| 2023/1/27 \| Newly created \| - \| \| Version 1.1 \| 2023/4/12 \| For approval by the Certified Review Board \| 2023/5/1 \| \| Version 1.2 \| 2023/6/30 \| Correction \| 2023/7/1 \| \| Version 1.3 \| 2023/10/20 \| Correction \| 2023/11/1 \| | Revision |
| **Research summary**  Inclusion and exclusion criteria  Exclusion criteria:  12) Patients receiving itraconazole, ritonavir-containing preparations, atazanavir, darunavir, fosamprenavir, cobicistat-containing preparations, or clarithromycin | **Research summary**  Inclusion and exclusion criteria  Exclusion criteria:  12) Patients receiving itraconazole, ritonavir-containing preparations, atazanavir, darunavir, fosamprenavir, cobicistat-containing preparations, clarithromycin, or ensitrevir | Revision of the package insert |
| **3. Inclusion criteria**  3.3. Exclusion criteria  12) Patients receiving itraconazole, ritonavir-containing preparations, atazanavir, darunavir, fosamprenavir, cobicistat-containing preparations, or clarithromycin | **3. Inclusion criteria**  3.3. Exclusion criteria  12) Patients receiving itraconazole, ritonavir-containing preparations, atazanavir, darunavir, fosamprenavir, cobicistat-containing preparations, clarithromycin, or ensitrevir | Revision of the package insert |
| **4. Treatment of the study participants**  **4.2. Patient enrollment**  2) Based on the results of the eligibility assessment, the eligibility and allocation test items will be entered into the EDC, and registration and allocation will be performed. A patient identification number will be assigned to the registered study participants (including those determined to be "ineligible" by the EDC), and a "study drug number" will also displayed. | **4. Treatment of the study participants**  **4.2. Patient enrollment**  2) Based on the results of the eligibility assessment, the eligibility and allocation test items will be entered into the EDC, and registration and allocation will be performed. A patient identification number will be assigned to the registered study participants, and a "study drug number" will also displayed. | Correction |
| **4. Treatment of the study participants**  **4.6. Overview of study drug**  **4.6.5. Usage precautions**  1) Contraindications (Do not administer to the following patients)  2. Patients receiving itraconazole, ritonavir-containing preparations, atazanavir, darunavir, fosamprenavir, cobicistat-containing preparations, or clarithromycin | **4. Treatment of the study participants**  **4.6. Overview of study drug**  **4.6.5. Usage precautions**  1) Contraindications (Do not administer to the following patients)  2. Patients receiving itraconazole, ritonavir-containing preparations, atazanavir, darunavir, fosamprenavir, cobicistat-containing preparations, clarithromycin, or ensitrevir | Revision of the package insert |
| **4. Treatment of the study participants**  **4.6. Overview of study drug**  **4.6.7. Contraindications for concomitant use (Do not use in combination)**  Drug name etc.  Itraconazole (Itrizol)  Ritonavir-containing preparations (Norvir, Kaletra)  Clarithromycin (Claris, Claricid) | **4. Treatment of the study participants**  **4.6. Overview of study drug**  **4.6.7. Contraindications for concomitant use (Do not use in combination)**  Drug name etc.  Itraconazole (Itrizol)  Ritonavir-containing preparations (Norvir, Kaletra)  Clarithromycin (Claris, Claricid)  Ensitrelville (Zocova) | Revision of the package insert |
| **4. Treatment of the study participants**  **4.7. Concomitant medications during the observation period**  Concomitant use of itraconazole, preparations containing ritonavir, atazanavir, darunavir, fosamprenavir, preparations containing cobicistat, and clarithromycin will be prohibited. | **4. Treatment of the study participants**  **4.7. Concomitant medications during the observation period**  Concomitant use of itraconazole, preparations containing ritonavir, atazanavir, darunavir, fosamprenavir, preparations containing cobicistat, clarithromycin, and ensitrelvir will be prohibited. | Revision of the package insert |

Created on June 25, 2024

Research Plan Revision Comparison Table 3 (ver1.3 to 1.4)

| **(Before change) October 20, 2023** | **(After change) June 25, 2024** | **Reason for change** |
| --- | --- | --- |
| October 20, 2023 ver1.3 | June 25, 2024 ver1.4 | Revision |
| Creation date: October 20, 2023  Version: 1.3 | Creation date: June 25, 2024  Version: 1.4 | Revision |
| **Revision history**   \| Version \| Creation and revision dates \| Reasons for revision \| Scheduled implementation date \| \| --- \| --- \| --- \| --- \| \| Version 1.0 (Draft Edition) \| 2023/1/27 \| Newly created \| - \| \| Version 1.1 \| 2023/4/12 \| For approval by the Certified Review Board \| 2023/5/1 \| \| Version 1.2 \| 2023/6/30 \| Correction \| 2023/7/1 \| \| Version 1.3 \| 2023/10/20 \| Correction \| 2023/11/1 \| | **Revision history**   \| Version \| Creation and revision dates \| Reasons for revision \| Scheduled implementation date \| \| --- \| --- \| --- \| --- \| \| Version 1.0 (Draft Edition) \| 2023/1/27 \| Newly created \| - \| \| Version 1.1 \| 2023/4/12 \| For approval by the Certified Review Board \| 2023/5/1 \| \| Version 1.2 \| 2023/6/30 \| Correction \| 2023/7/1 \| \| Version 1.3 \| 2023/10/20 \| Correction \| 2023/11/1 \| \| Version 1.4 \| 2024/6/25 \| Correction \| 2024/8/13 \| | Revision |
| **Research summary**  Endpoints  Other endpoints  5. Changes in abundance of up to 181 proteins comprehensively analyzed by proteomic analysis (Target 96 panel) at 24 weeks after the protocol treatment initiation as compared to that at baseline (exploratory endpoint) | **Research summary**  Endpoints  Other endpoints  5. Changes in abundance of up to 181 proteins comprehensively analyzed by proteomic analysis (Olink Target 96 CVD III analysis, Olink Target 96 Inflammation analysis) at 24 weeks after the protocol treatment initiation as compared to that at baseline (exploratory endpoint) | Added information |
| **2. Research contents**  2.1. Endpoints  3) Other endpoints:  5. Changes in abundance of up to 181 proteins comprehensively analyzed by proteomic analysis (Target 96 panel) at 24 weeks after the protocol treatment initiation as compared to that at baseline (exploratory endpoint) | **2. Research contents**  2.1. Endpoints  3) Other endpoints:  5. Changes in abundance of up to 181 proteins comprehensively analyzed by proteomic analysis (Olink Target 96 CVD III analysis, Olink Target 96 Inflammation analysis) at 24 weeks after the protocol treatment initiation as compared to that at baseline (exploratory endpoint) | Added information |
| **2. Research contents**  2.4. Method of administration of study drug  2.4.1. Method of administration of study drug  Quoted from the 3rd edition of the package insert | **2. Research contents**  2.4. Method of administration of study drug  2.4.1. Method of administration of study drug  Quoted from the 6th edition of the package insert | Corrections accompanying the revision of the package insert |
| **4. Treatment of the study participants**  **4.5. Central testing**  Blood biomarkers (pentosidine) and urinary biomarkers (type IV collagen, α1-MG, β2-MG, NGAL, NAG, L-FABP creatinine ratio), ... to ... Moreover, samples for proteomics analysis will be collected by SRL, Inc., and after being frozen and stored, they will be transported after anonymization to a testing institution designated by the Department of Cardiology at Saga University Hospital for proteomics analysis.  Detailed procedures for the collection, transportation, and measurement of samples, as well as the provision of test results, will follow the standard operating procedures separately established by SRL Corporation and the testing institutions designated by the Department of Cardiology at Saga University Hospital. | **4. Treatment of the study participants**  **4.5. Central testing**  Blood biomarkers (pentosidine) and urinary biomarkers (type IV collagen, α1-MG, β2-MG, NGAL, NAG, L-FABP creatinine ratio), ... to ... Moreover, samples for proteomics analysis will be collected by SRL, Inc., and after being frozen and stored, they will be transported after anonymization to Pharma Foods Co., Ltd. Apro Science Group for proteomics analysis.  Detailed procedures for the collection, transportation, and measurement of samples, as well as the provision of test results, will follow the standard operating procedures separately established by SRL Corporation and Pharma Foods Co., Ltd. Apro Science Group. | Updating with the latest information |
| **4. Treatment of the study participants**  **4.6. Overview of study drug**  **4.6.5. Usage precautions**  1) Contraindications (Do not administer to the following patients)  2. Patients receiving itraconazole, ritonavir-containing preparations, atazanavir, darunavir, fosamprenavir, cobicistat-containing preparations, clarithromycin, or ensitrevir | **4. Treatment of the study participants**  **4.6. Overview of study drug**  **4.6.5. Usage precautions**  1) Contraindications (Do not administer to the following patients)  2. Patients receiving itraconazole, posaconazole, voriconazole, ritonavir-containing preparations, atazanavir, darunavir, fosamprenavir, cobicistat-containing preparations, clarithromycin, or ensitrevir | Revision of the package insert |
| **4. Treatment of the study participants**  **4.6. Overview of study drug**  **4.6.7. Contraindications for concomitant use (Do not use in combination)**  Drug name  Itraconazole (Itrizol)  Ritonavir-containing preparations (Norvir, Kaletra)  Cobicistat-containing preparations (Genvoya, Stribild, Simtuza, Prezicovic)  to | **4. Treatment of the study participants**  **4.6. Overview of study drug**  **4.6.7. Contraindications for concomitant use (Do not use in combination)**  Drug name  Itraconazole (Itrizol)  Posaconazole (Noxafil)  Voriconazole (Vfend)  Ritonavir-containing preparations (Norvir, Kaletra)  Cobicistat-containing preparations (Genvoya, Simtuza, Prezicovic)  to | Additions and deletions due to a revision of the package insert |
| **4. Treatment of the study participants**  **4.7. Concomitant medications during the observation period**  Concomitant use of itraconazole, preparations containing ritonavir, atazanavir, darunavir, fosamprenavir, preparations containing cobicistat, clarithromycin, and ensitrelvir will be prohibited. | **4. Treatment of the study participants**  **4.7. Concomitant medications during the observation period**  Concomitant use of itraconazole, posaconazole, voriconazole, preparations containing ritonavir, atazanavir, darunavir, fosamprenavir, preparations containing cobicistat, clarithromycin, and ensitrelvir will be prohibited. | Additions due to a revision of the package insert |
| **5. Efficacy and safety evaluation**  **5.3 Other endpoints**  5. Changes in abundance of up to 181 proteins comprehensively analyzed by proteomic analysis (Target 96 panel) at 24 weeks after the protocol treatment initiation as compared to that at baseline (exploratory endpoint) | **5. Efficacy and safety evaluation**  **5.3 Other endpoints**  5. Changes in abundance of up to 181 proteins comprehensively analyzed by proteomic analysis (Olink Target 96 CVD III analysis, Olink Target 96 Inflammation analysis) at 24 weeks after the protocol treatment initiation as compared to that at baseline (exploratory endpoint) | Added information |
| **5. Efficacy and safety evaluation**  **5.4 Safety endpoints**  **5.4.6 Predictability**  The latest Finerenone package insert  The side effects information listed in the 3rd edition of the Finerenone package insert (revised September 2022) is as described in "4.6.9 Side Effects." | **5. Efficacy and safety evaluation**  **5.4 Safety endpoints**  **5.4.6 Predictability**  The latest Finerenone package insert  The side effects information listed in the 6th edition of the Finerenone package insert (revised June 2024) is as described in "4.6.9 Side Effects." | Corrections accompanying the revision of the package insert |
| **7.4. Efficacy analysis**  **7.4.1. Efficacy analysis**  C) Analysis of other endpoints  e. Changes in abundance of up to 181 proteins comprehensively analyzed by proteomic analysis (Target 96 panel) at 24 weeks after the protocol treatment initiation as compared to that at baseline (exploratory endpoint): | **7.4. Efficacy analysis**  **7.4.1. Efficacy analysis**  C) Analysis of other endpoints  e. Changes in abundance of up to 181 proteins comprehensively analyzed by proteomic analysis (Olink Target 96 CVD III analysis, Olink Target 96 Inflammation analysis) at 24 weeks after the protocol treatment initiation as compared to that at baseline (exploratory endpoint): | Added information |
| **12. Handling and storage of records (including data)**  4) Paper records or data regarding the central testing results will be sent to the chief principal investigator from SRL Corporation and the testing institutions designated by the Department of Cardiology, Saga University Hospital. | **12. Handling and storage of records (including data)**  4) Paper reports or data regarding the central measurement results will be sent to the chief principal investigator from SRL Inc. and Pharma Foods Co., Ltd. Apro Science Group. | Updating with the latest information |
| **16. Explanation to the study participants and their informed consent**  **16.5. The possibility that samples and information obtained from study participants will be used for future research**  The remaining samples after baseline and 24-week blood and urinary biomarker measurements will be stored for a certain period after the study completion, and then discarded, in accordance with the procedures of SRL, Inc. Moreover, the remaining samples after proteomics analysis will be discarded by the Department of Cardiology, Saga University Hospital after the completion of the study. | **16. Explanation to the study participants and their informed consent**  **16.5. The possibility that samples and information obtained from study participants will be used for future research**  The remaining samples after baseline and 24-week blood and urinary biomarker measurements will be stored for a certain period after the study completion, and then discarded, in accordance with the procedures of SRL, Inc. Moreover, the remaining samples after proteomics analysis will be stored for a certain period after the study completion, and then discarded, in accordance with the procedures of Pharma Foods Co., Ltd. Apro Science Group. | Updating with the latest information. |
| **17. Research funding and conflicts of interest**  **17.4. Conflict of interest**  The chief principal investigator receives a personal income of > 1 million yen per year from Bayer Yakuhin, Ltd.; however, this will not result in any undue influence of the company on the results of this study. Moreover, a principal investigator receives a personal income of > 1 million yen per year, and a co-investigator receives a personal income of > 2.5 million yen per year, from Bayer Yakuhin Co., Ltd.; however, this will not result in any undue influence of the company on the results of this study. | **17. Research funding and conflicts of interest**  **17.4. Conflict of interest**  A co-investigator receives a personal income of > 1 million yen per year from Bayer Yakuhin Ltd.; however, this will not result in any undue influence of the company on the results of this study. | Correction based on the latest conflict of interest status |

**Supplementary Text S2. Study sites list**

**Study site, City (Chief investigator)**

1.　Saga University, Saga (Dr. Koichi Node)

2.　Oita University, Yufu (Dr. Naohiko Takahashi)

3.　Osaka Metropolitan University, Osaka (Dr. Daiju Fukuda)

4.　University of Occupational and Environmental Health, Japan, Kitakyushu (Dr. Yosuke Okada)

5.　JR Hiroshima Hospital, Hiroshima (Dr. Hiroki Teragawa)

6.　Juntendo University Shizuoka Hospital, Shizuoka (Dr. Satoru Suwa)

7.　St. Marianna University School of Medicine, Kawasaki (Dr. Keisuke Kida)

8.　Toho University Ohashi Medical Center, Tokyo (Dr. Masao Moroi)

9.　Dokkyo Medical University Saitama Medical Center, Koshigaya (Dr. Isao Taguchi)

10.　Dokkyo Medical University School of Medicine, Mibu (Dr. Shigeru Toyoda)

11.　Fukushima Medical University, Fukushima (Dr. Michio Shimabukuro)

12.　Mitsui Memorial Hospital, Tokyo (Dr. Kengo Tanabe)

13.　Wakamatsu Hospital of the University of Occupational and Environmental Health, Japan, Kitakyushu (Dr. Kenichi Tanaka)

**Supplementary Text S3**

**Effects of FInerenone on Vascular stiffnEss and cardiorenal biomarkerS in Type 2 diAbetes and chRonic kidney disease**

**(FIVE-STAR)**

**Statistical Analysis Plan**

Principal Investigator

Affiliation: Department of Cardiology, Saga University Hospital

Name: Koichi Node

Head of Statistical Analysis

Affiliation: Clinical Research Division, Clinical Medicine Promotion Organization

Name: Takumi Imai

Creation date: September 30, 2024 (Version 1.0)

Creation/Revision History

| Version number | Creation/Revision Dates | Creation/Revision Contents | Author |
| --- | --- | --- | --- |
| 1.0 | September 30, 2024 | First edition | Takumi Imai |
|  |  |  |  |
|  |  |  |  |

**Purpose**

1. The purpose of this statistical analysis plan is to report the methods for statistical analysis for the study protocol in the "Effects of FInerenone on Vascular Stiffness and Cardiorenal Biomarkers in Chronic Kidney Disease with Type 2 Diabetes (FIVE-STAR)" study (henceforth referred to as "this study").

## **Changes from the research plan**

None

## **Abbreviations**

| Abbreviation | Definition (English) | Definition (Japanese) |
| --- | --- | --- |
| ACE | Angiotensin-converting enzyme | Angiotensin-converting enzyme |
| AI | Augmentation index | Amplification factor / increased pressure factor |
| ARB | Angiotensin II receptor blocker | Angiotensin receptor blocker |
| ARNI | Angiotensin receptor-neprilysin inhibitors | Angiotensin receptor neprilysin inhibitors |
| BMI | Body mass index | Body mass index |
| CAVI | Cardio-ankle vascular index | Cardio-ankle vascular index |
| Child-Pugh | - | An index showing the degree of liver damage |
| CKD | Chronic kidney disease | Chronic kidney disease |
| ClinicalTrials.gov | - | US Clinical Trials Registry Database |
| CRB | Certified review board | Certified clinical research review board |
| CRF | Case report form | Case report form |
| DPP-4 | Dipeptidyl peptidase 4 | Dipeptidyl peptidase 4 |
| E | - | Early diastolic flow velocity waveform of mitral valve orifice |
| E/e’ | - | Early diastolic left ventricular inflow mitral annular velocity ratio |
| EDC | Electric data capture | Electronic data capture |
| eEV | Estimated extracellular volume | Estimated extracellular fluid volume |
| eGFR | Estimated glomerular filtration rate | Estimated glomerular filtration rate |
| ESA | Erythropoiesis-stimulating agent | Erythropoiesis-stimulating agent |
| FAS | Full analysis set | Full analysis set |
| GLP-1 | Glucagon-like peptide-1 | Glucagon-like peptide-1 |
| HbA1c | Hemoglobin A1c | Hemoglobin A1c |
| HFrEF | Heart failure with reduced ejection fraction | Heart failure with reduced ejection fraction |
| HIF-PH | Hypoxia-inducible factor prolyl-hydroxylase inhibitor | Hypoxia-inducible factor prolyl hydroxylase |
| IgA | Immunoglobulin A | Immunoglobulin A |
| jRCT | Japan Registry of Clinical Trials | Japan Registry of Clinical Trials |
| LAD | Left atrial dimension | Left atrial dimension |
| LAVI | Left atrial volume index | Left atrial volume index |
| L-FABP | Liver-type fatty acid-binding protein | L-type fatty acid binding protein |
| LLT | Lowest level term | Lowest level term |
| LVEF | Left ventricular ejection fraction | Left ventricular ejection fraction |
| LVMI | Left ventricular mass index | Left ventricular mass index |
| MedDRA/J | Medical Dictionary for Regulatory Activities/Japanese version | Medical Dictionary for Regulatory Activities/Japanese version |
| MMRM | Mixed-effects models for repeated measures | Mixed-effects models for repeated measures |
| MRA | Mineralocorticoid receptor antagonist | Mineralocorticoid receptor antagonists |
| NAG | N-acetyl-β-d-glucosaminidase | N-acetylglucosaminidase |
| NGAL | Neutrophil gelatinase-associated lipocalin | Neutrophil gelatinase-associated lipocalin |
| NYHA | New York Heart Association | New York Heart Association |
| PPS | Per-protocol set | Per-protocol set |
| PT | Preferred term | Preferred term |
| RA | Renin-angiotensin | Renin-angiotensin |
| Septal e’, lateral e’ | - | Septal and lateral mitral annulus movement speed |
| SGLT2 | Sodium glucose cotransporter 2 | Sodium-glucose cotransporter 2 |
| SOC | System organ class | System organ class |
| SS | Safety set | Safety set |
| TEAE | Treatment-emergent adverse event | Adverse events occurring after baseline |
| UACR | Urine albumin-to-creatinine ratio | Urinary albumin/creatinine ratio |
| α1-MG | α1-microglobulin | α1-microglobulin |
| β2-MG | β2-microglobulin | β2-microglobulin |

--: Indicates no appropriate term.

# **Research summary**

## **Research objective**

To evaluate the effects of finerenone on vascular stiffness using cardio-ankle vascular index (CAVI) and cardiorenal biomarkers in patients with chronic kidney disease and type 2 diabetes.

## **Study drug**

Finerenone (brand name: Kerendia® tablets 10 mg/20 mg)

## **Study design**

Investigator-initiated, multicenter, prospective, parallel-group, placebo-controlled, double-blind, randomized controlled trial

## **Study participants**

Patients with chronic kidney disease and type 2 diabetes

## **Target number of cases**

100 cases (Finerenone group, 50 cases; Placebo group, 50 cases)

## **Research methodology**

After verifying the eligibility of patients providing written consent, patients meeting the inclusion criteria will be enrolled in the study. Patients will be randomized (allocated) to the finerenone or placebo groups and will undergo double-blind administration, observation, and prescribed tests at baseline (before the study drug administration) and 4, 12, and 24 weeks after study drug administration. In principle, baseline tests will be conducted within 60 days of obtaining consent, and study drug administration will be initiated the following day.

# **Analysis** **population**

## **Randomized set**

All participants who were allocated to either treatment group following study registration.

## **Efficacy analysis set**

Regarding efficacy, the full analysis set (FAS) will be the primary analysis set, and a supplementary analysis will be conducted with the per-protocol set (PPS) for sensitivity analysis.

### **Full analysis set (FAS)**

In accordance with the intention-to-treat principle, the FAS excluding the following patients from the randomized set will be used for efficacy analysis:

- Patients who withdrew consent after enrollment
- Patients who were identified as ineligible after enrollment
- Patients who never received protocol treatment after allocation
- Patients with no data on efficacy after protocol treatment initiation

### **Per-protocol set (PPS)**

Among the FAS, the patients without any significant deviations from the study plan will be included in the PPS and will be used for supplementary efficacy analysis.

The data center will evaluate deviations from the study plan as cases accumulate. Furthermore, before the study data set is finalized, a case review meeting will be conducted with the principal investigator, head of statistical analysis, data center, and monitoring staff to define and identify cases with significant deviations from the study plan.

## **Safety analysis set (SS)**

Randomized patients administered at least one dose of the protocol treatment will be included in the SS.

# **Endpoints**

## **Primary endpoint**

Change in CAVI at 24 weeks after protocol treatment initiation as compared to that at baseline

## **Secondary endpoints**

1. Proportional change in geometric mean of urine albumin-to-creatinine ratio (UACR) at 12 and 24 weeks after the protocol treatment initiation as compared to that at baseline (important secondary endpoint)
2. Proportional change in geometric mean of blood biomarkers (pentosidine) and urinary biomarkers (type IV collagen, α1-MG, β2-MG, NGAL, NAG, L-FABP creatinine ratio) at 24 weeks after the protocol treatment initiation as compared to that at baseline

## **Other endpoints**

1. Changes from baseline in vital signs (weight, body mass index (BMI), estimated extracellular volume (eEV), blood pressure, pulse pressure, and pulse rate in the clinic, in addition to blood pressure, pulse pressure, and pulse rate at home) at 4, 12, and 24 weeks after the protocol treatment initiation
2. Changes from baseline in blood test indices (serum creatinine, estimated glomerular filtration rate (eGFR), serum cystatin C*, serum potassium, Hemoglobin A1c (HbA1c*), plasma (or serum) aldosterone concentration**, plasma (or serum) renin activity or concentration**) at 4, 12, and 24 weeks after the protocol treatment initiation (*evaluated only at baseline, and 12, and 24 weeks; **evaluated only at baseline and 24 weeks)
3. Changes from baseline in augmentation index (AI) and % mean arterial pressure at 24 weeks after the protocol treatment initiations
4. Changes from baseline in cardiac function indices (left ventricular ejection fraction (LVEF), septal e', lateral e', E, E/e'*, left ventricular mass index (LVMI), left atrial dimension (LAD), left atrial volume index (LAVI)) assessed by echocardiography 24 weeks after the protocol treatment initiation

*Three types of E/e' will be evaluated including E/septal e', E/lateral e', and E/average (septal e', lateral e')**

** average (septal e', lateral e') = (septal e' + lateral e')/2. If septal e' is missing, average (septal e', lateral e') = lateral e'. If lateral e' is missing, average (septal e', lateral e') = septal e'. If both are missing, average (septal e', lateral e') is also missing.

1. Changes in abundance of up to 181 proteins comprehensively analyzed by proteomic analysis (Olink Target 96 CVD III analysis, Olink Target 96 Inflammation analysis), 24 weeks after the protocol treatment initiation as compared to that at baseline (exploratory endpoint)

## **Safety endpoints**

Adverse events including hyperkalemia occurring after the protocol treatment initiation

# **Data handling**

## **Handling missing data**

Unless otherwise specified, missing values will be treated as missing values and will not be imputed with other data or used in tabulations or descriptive statistics calculations.

## **Handling of data at the time of discontinuation**

In safety analysis, data at the time of discontinuation will be treated as data from the most recent measurement after a specified study visit before the discontinuation. If a measurement at the time of discontinuation is conducted after the last study visit specified, it will not be included in the descriptive statistics compilation or calculation.

## **Defining calculation items**

The change in endpoint A (participant-level) at a certain time point, t, is defined as follows:

Change in endpoint A = Endpoint A(t) - Endpoint A(baseline)

The proportional change (participant-level) of endpoint A at a certain time point, t, is defined as follows:

Proportional change of endpoint A = Endpoint A(t) / Endpoint A(baseline)

The change in the mean value of endpoint A (population-level) at a certain time point, t, is defined as follows:

Change in the mean value of endpoint A(t) = mean value of endpoint A(t) - mean value of endpoint A(baseline)

The proportional change of the geometric mean of endpoint A (population-level) at a certain time point, t, is defined as follows:

Proportional change in geometric mean of endpoint A(t) = geometric mean of endpoint A(t)/geometric mean of endpoint A(baseline)

= exp (ln (endpoint A) mean(t) - ln (endpoint A) mean(baseline))

= exp (ln (endpoint A) mean change (t))

# **Output statistics and significance levels for hypothesis tests**

## **Descriptive statistics**

The outputs will be number of cases, mean, standard deviation, minimum, 25th percentile, median, 75th percentile, and maximum and number of missing values.

## **Inferential statistics**

The mean and 95% confidence interval based on t-distribution or Wald method will be the output. For variables with normality of the residuals not holding, the mean and 95% confidence interval based on t-distribution or Wald method will be calculated using the natural logarithm scale, and the output will be values converted to the original scale by inverse logarithm transformation.

## **Analysis using linear models and mixed-effect models for repeated measures (MMRM)**

The estimation results of group effects (estimated values, 95% confidence intervals based on t-distribution or Wald method), and *P* values of the hypothesis test for the null hypothesis of "no group effect" will be the output. The significance level was set at a two-sided *P* value of < 0.05. For variables with normality of the residuals not holding, the analysis is performed using the natural logarithm scale, and the outputs will be the estimates and 95% confidence intervals after being converted to the original scale using an inverse logarithmic transformation.

## **Frequency counting**

The number and proportion (%) of the cases will be the output. If needed, the 95% confidence interval of the proportion of cases will be calculated using the Clopper–Pearson method.

## **Addressing multiplicity of tests**

In this study, the evaluation of treatment efficacy will be based on the primary analysis of the primary endpoint targeting the full analysis set (FAS). Multiplicity adjustments will not be performed for supplementary analyses of the primary endpoint, analyses of the per-protocol set (PPS), or analyses of secondary endpoints. However, as an exception, for proteomics analyses, methods to address multiplicity within the proteomics analysis will be specified separately.

# **Statistical** **analysis**

## **Patient classification**

The following numbers of cases will be verified with those with consent obtained:

- Number of cases with consent obtained
- Number of registered cases
- Number of unregistered cases
- Number of allocated cases
- Number of unallocated cases
- Number of SS cases in each treatment group
- Number of cases excluded from SS in each treatment group
- Number of FAS cases in each treatment group
- Number of cases excluded from the FAS in each treatment group
- Number of PPS cases in each treatment group
- Number of cases excluded from the PPS in each treatment group
- Number of patients completing the 24-week follow-up in each treatment group in the FAS
- Number of patients not completing the 24-week follow-up in each treatment group in the FAS
- Number of patients completing 24 weeks of treatment in each treatment group in the FAS
- Number of patients not completing 24 weeks of treatment in each treatment group in the FAS

For unallocated cases, the reasons for no allocation will be tabulated. For allocated patients discontinuing before or after the treatment initiation, the reasons for their exclusion will be tabulated by treatment group. For allocated patients excluded from the SS, patients in the SS excluded from the FAS, patients in the FAS excluded from the PPS, and the reasons for their exclusion will be tabulated by treatment group. For patients in each treatment group not completing the 24-week follow-up or 24-week treatment, the reasons for their exclusion will be tabulated.

## **Demographic and clinical characteristics**

For each population (randomized, FAS, PPS, SS), descriptive statistics and frequency tabulations of the following items will be performed for the entire population and for each treatment group.

- Age (at the time of registration)
- eGFR (at the time of registration)
- UACR (at the time of registration)
- HbA1c (at time of registration)
- Serum potassium (at time of registration)
- LVEF (at the time of enrollment)
- Duration of diabetes (at the time of enrollment)
- Height (baseline)
- Weight (baseline)
- BMI (baseline)
- Clinic blood pressure (systolic and diastolic) (baseline)
- Clinic pulse (baseline)
- Home blood pressure (systolic and diastolic) (baseline)
- Home pulse (baseline)
- Gender (at the time of registration)
- Use of SGLT2 inhibitors (at time of registration)
- Presence or absence of complications or history of comorbidities (hypertension, dyslipidemia, ischemic heart disease, stroke, lower limb arteriosclerosis obliterans, heart failure) (at the time of registration)
- Primary underlying disease of chronic kidney disease (diabetic nephropathy, non-diabetic nephropathy: chronic glomerulonephritis, nephrosclerosis, polycystic kidney disease, collagen disease-related nephropathy, others) (at the time of registration)

## **Description of the medication adherence for the study treatment**

For the SS and FAS, descriptive statistics and frequency estimation will be conducted for medication adherence for the study treatment by treatment group and study visit.

## **Concomitant medication use description**

Descriptive statistics and frequency estimation will be conducted for concomitant medication use by treatment group and study visit for the FAS. The concomitant medications that will be assessed include ACE inhibitors, ARBs, ARNIs, β-blockers, calcium antagonists, diuretics, statins, insulin, metformin, SGLT2 inhibitors, GLP-1 receptor agonists, DPP-4 inhibitors, iron preparations, ESAs, HIF-PH inhibitors, and hyperkalemia medications.

## **Analysis of the primary endpoint**

The following analysis will be performed on the FAS as the primary analysis. Furthermore, an analysis will be conducted using the PPS to confirm the robustness of the results.

1. Descriptive statistics calculations will be conducted, and histograms will be plotted to assess CAVI and its participant-level change and proportional change by treatment group and study visit. Inferential statistics calculations will be used to estimate CAVI mean and change, and geometric mean and proportional change at population-level by treatment group and study visit.
2. As the primary analysis for the primary endpoint, change in CAVI at 24 weeks will be analyzed using a linear regression model with baseline values as covariates and allocation as fixed effects. To assess treatment effect, the between-group difference in CAVI change using the least squares mean at 24 weeks will be estimated using the placebo group as a reference. The output will be the *P* value of the test for the null hypothesis that the between-group difference in change is 0. Moreover, the presence or absence of a treatment effect will be determined using the criteria of a two-sided *P* value of < 0.05.
3. As a supplementary analysis for the primary endpoint, change in logarithmic CAVI at 24 weeks will be analyzed using a linear regression model with logarithmic baseline values as covariates and allocation as fixed effects. To assess treatment effect, the between-group ratio of the proportional change in CAVI geometric mean using the least squares mean at 24 weeks will be estimated using the placebo group as a reference. The output will be the *P* value of the test for the null hypothesis that the between-group ratio is 1.

## **Analysis of secondary endpoints**

The following analyses will be conducted with the FAS.

1. Proportional change in geometric mean of UACR at 12 and 24 weeks after the protocol treatment initiation as compared to that at baseline (important secondary endpoint):
2. Descriptive statistics calculations will be conducted, and histograms will be plotted for UACR and its participant-level proportional change by treatment group and study visit. Inferential statistics calculations will be conducted for the geometric mean of population-level UACR and its proportional change by treatment group and study visit.
3. Change in logarithmic UACR values at 12 and 24 weeks will be analyzed using a mixed-effects model (MMRM) with the logarithmic baseline value as a covariate, allocation, study visit and their interactions as fixed effects, and the patient as a random effect. The restricted maximum likelihood (REML) method will be used for the analysis with a compound symmetry structure specified as the covariance structure. The Kenward–Roger method will be used to calculate the degrees of freedom. To assess treatment effects, the between-group ratio of the proportional change in UACR geometric mean using the least squares mean at 12 weeks and the between-group ratio of the proportional change in UACR geometric mean using the least squares mean at 24 weeks will be estimated using the placebo group as the reference. At each time point, the output will be *P* value for testing the null hypothesis that the between-group ratio is 1.
4. Proportional change in geometric mean of blood biomarkers (pentosidine) and urinary biomarkers (type IV collagen, α1-MG, β2-MG, NGAL, NAG, L-FABP creatinine ratio) at 24 weeks after the protocol treatment initiation as compared to that baseline:
5. Descriptive statistics will be calculated, and histograms will be plotted for each item and its participant-level proportional change by treatment group and study visit. Inferential statistics calculations will be conducted for the population-level geometric mean and its proportional change by treatment group and study visit.
6. The logarithmic values of each item at 24 weeks will be analyzed using a linear regression model with the logarithmic values of baseline values as covariates and allocation as fixed effects. To assess treatment effect, the between-group ratio of the proportional change of the geometric mean using the least squares mean at 24 weeks will be estimated using the placebo group as a reference. The output will be *P* value of the test for the null hypothesis that the between-group ratio is 1.

## **Analysis of other endpoints**

The following analyses will be performed with the FAS.

1. Changes from baseline in vital signs (weight, BMI, eEV, blood pressure, pulse pressure, and pulse rate in the clinic, and blood pressure, pulse pressure, and pulse rate at home) at 4, 12, and 24 weeks after the protocol treatment initiation:
2. Descriptive statistics calculations will be conducted, and histograms will be plotted for each item and its participant-level change by treatment group and study visit. Inferential statistics calculations will be conducted for the population-level mean and its change by treatment group and study visit.
3. For each item at each time point, analysis will be performed using MMRM with the baseline values as covariates, allocation, study visit and their interactions as fixed effects, and the patient as a random effect. The REML method will be used for the analysis with compound symmetry structure specified as the covariance structure. The Kenward–Roger method will be used to calculate the degrees of freedom. To assess the treatment effect at each time point, the between-group difference in the change in the mean value using the least squares mean will be estimated using the placebo group as a reference. At each time point, the output will be the *P* value for testing the null hypothesis that the between-group ratio is 0.
4. Changes from baseline in blood test indices (serum creatinine, eGFR, serum cystatin C*, serum potassium, HbA1c*, plasma (or serum) aldosterone concentration**, plasma (or serum) renin activity or concentration**) at 4, 12, and 24 weeks after the protocol treatment initiation (*evaluated only at baseline, and 12, and 24 weeks; **evaluated only at baseline and 24 weeks):
5. Descriptive statistics calculations will be conducted, and histograms will be plotted for each item and its participant-level change by treatment group and study visit. Inferential statistics calculations will be conducted for the population-level mean and its change by treatment group and study visit. If the distribution of values deviates significantly from normal distribution, the proportional change will be described with estimation of the geometric mean and its proportional change as appropriate.
6. For each item marked with an "*," at each time point, analysis is performed using a MMRM with the baseline values as covariates, allocation, study visit and their interactions as fixed effects, and the patient as a random effect. The REML method will be used for the analysis with a compound symmetry structure specified as the covariance structure. The Kenward–Roger method will be used to calculate the degrees of freedom. To assess the treatment effect at each time point, the between-group difference in the change in the mean value using the least squares mean will be estimated using the placebo group as a reference. At each time point, the output will be the *P* value for testing the null hypothesis that the between-group ratio is 0.

For items marked with "**," analysis will performed using a linear regression model with baseline values as covariates and allocation as fixed effects for each item at 24 weeks. To assess the treatment effect, the between-group difference in the change using the least squares mean at 24 weeks will be estimated using the placebo group as a reference. The output will be *P* value for testing the null hypothesis that the between-group difference in change is 0.

In any analysis, if the distribution of values significantly deviates from a normal distribution, logarithmic values will be used for analysis as appropriate.

1. Changes from baseline in AI and % mean arterial pressure at 24 weeks after the protocol treatment initiation:
2. Descriptive statistics calculations will be conducted, and histograms will be plotted for each item and its participant-level change by treatment group and study visit. Inferential statistics calculations will be conducted for the population-level mean and its change by treatment group and study visit. If the distribution of values deviates significantly from normal distribution, the proportional change will be described with estimation of the geometric mean and its proportional change as appropriate.
3. Analysis will be performed using a linear regression model with baseline values as covariates and allocation as fixed effects for each item at 24 weeks. To assess the treatment effect, the between-group difference in the change using the least squares mean at 24 weeks will be estimated using placebo group as a reference. The output will be the *P* value for testing the null hypothesis that the between-group difference in change is 0. If the distribution of values significantly deviates from a normal distribution, logarithmic values will be used for analysis as appropriate.
4. Changes from baseline in cardiac function indices (LVEF, septal e', lateral e', E, E/e'*, LVMI, LAD, LAVI) assessed by echocardiography 24 weeks after the protocol treatment initiation:
5. Descriptive statistics calculations will be conducted, and histograms will be plotted for each item and its participant-level change by treatment group and study visit. Inferential statistics calculations will be conducted for the population-level mean and its change by treatment group and study visit. If the distribution of values deviates significantly from normal distribution, the proportional change described with estimation of the geometric mean and its proportional change as appropriate.
6. Analysis will be performed using a linear regression model with baseline values as covariates and allocation as fixed effects for each item at 24 weeks. To assess the treatment effect, the between-group difference in the change using the least squares mean at 24 weeks will be estimated using the placebo group as a reference. The output will be the *P* value for testing the null hypothesis that the between-group difference in change is 0. If the distribution of values significantly deviates from a normal distribution, the logarithmic values will be used for analysis as appropriate.
7. Changes in abundance of up to 181 proteins comprehensively analyzed by proteomic analysis (Olink Target 96 CVD III analysis, Olink Target 96 Inflammation analysis) at 24 weeks after the protocol treatment initiation as compared to that at baseline (exploratory endpoint):
8. Descriptive statistics calculations will be conducted for each item and its participant-level change by treatment group and study visit. If the distribution of values deviates significantly from normal distribution, the proportional change will be described as appropriate.
9. Comprehensive comparisons between groups will be performed for each variable using *t*-tests. The Benjamini–Hochberg method will be used to control the false discovery rate at < 5% under multiple comparisons.

## **Subgroup analysis for the primary endpoint**

For the primary endpoint, subgroup analysis based on the variables listed below will be performed with the FAS. If information related to the definition of a subgroup is missing, the case will be excluded from the subgroup analysis.

- Age at time of registration (median and above/below median)
- Age at time of registration (70 and above/below 70 years)
- Sex (male/female)
- BMI at baseline (median and above/below median)
- Clinic systolic blood pressure at baseline (median and above/below median)
- Clinic systolic blood pressure at baseline (130 mmHg and above/below 130 mmHg)
- CAVI at baseline (median and above/below median)
- CAVI at baseline (9 and above/below 9)
- eGFR at time of registration (median and above/below median)
- eGFR at time of registration (45 mL/min/1.73 m^2^ and above/below 45 mL/min/1.73 m^2^)
- eGFR at time of registration (60 mL/min/1.73 m^2^ and above/below 60 mL/min/1.73 m^2^)
- UACR at time of registration (median and above/below median)
- UACR at time of enrollment (300 mg/g.cr. and above/below 300 mg/g.cr.)
- HbA1c at time of registration (median and above/below median)
- Serum potassium levels at time of registration (median and above/below median)
- LVEF at time of registration (median and above/below median)
- LVEF at time of registration (40% and above/below 40%)
- LVEF at time of registration (60% and above/below 60%)
- Duration of diabetes at time of registration (median and above/below median)
- History of hypertension at time of registration
- History of ischemic heart disease, stroke, or lower limb arteriosclerosis at time of

registration

- History of heart failure at time of registration
- Use of ACE inhibitors, ARBs, or ARNIs at baseline
- Use of concomitant SGLT2 inhibitors at baseline
- Use of concomitant GLP-1 receptor agonists at baseline
- KDIGO chronic kidney disease severity classification at time of registration (moderate,

high, very high)

## **Safety endpoints**

The following analyses will be performed with the SS. Reported adverse events will be assigned a low-level term (LLT) code using the Medical Dictionary for Regulatory Affairs, Japanese (MedDRA/J). The version of MedDRA/J used for the analysis will be the latest version at the time of database fixation.

1. Serum potassium levels (blood test parameter)
2. Descriptive statistics calculations will be conducted, and histograms will be plotted for serum potassium levels and its participant-level change by treatment group and study visit. Inferential statistics calculations will be conducted for the population-level mean and its change by treatment group and study visit. If the distribution of values deviates significantly from normal distribution, the proportional change will be described with the geometric mean and its proportional change as appropriate.
3. For serum potassium levels at each time point, analysis will be performed using MMRM with the baseline values as covariates, allocation, study visit and their interactions as fixed effects, and the patient as a random effect. The REML method will be used for the analysis with compound symmetry structure set as the covariance structure. The Kenward–Roger method will be used to calculate the degrees of freedom. To assess the treatment effect at each time point, the between-group difference in the change in the mean value using the least squares mean will be estimated using the placebo group as the reference. At each time point, the output will be the *P* value for testing the null hypothesis that the between-group ratio is 0.
4. The frequency of serum potassium levels exceeding 5.5 and 6.0 mEq/L, and falling below 3.5 mEq/L will be calculated for baseline, 4, 12, and 24 weeks after the protocol treatment initiation.
5. At 4, 12, or 24 weeks after the protocol treatment initiation, frequency of events with serum potassium exceeding 5.5 and 6.0 mEq/L, or falling below 3.5 mEq/L will be tabulated. The denominator for the tabulation will be the number of SS cases with serum potassium data after the protocol treatment initiation.
6. Hyperkalemia (adverse event)

The frequency of hyperkalemia reported as an adverse event will be tabulated. The denominator for the tabulation will be the SS.

1. Adverse events and side effects

Adverse events (TEAEs) occurring after the protocol treatment initiation will be calculated. Adverse events causally related to the study drug will be considered under side effects.

If an adverse event with the same preferred term (PT) occurred multiple times in one case, the number of occurrences will be considered as the number of events, and the number of cases will be considered separately as one case.

For all adverse events and side effects, the number of occurrences, number of cases, and incidence rate with 95% confidence intervals using the Clopper–Pearson method will be calculated for each treatment group.

The number of events, number of cases, and incidence rate will be determined by system organ class (SOC) and PT classification for each treatment group.

# **Statistical analysis and tabulation** **software**

| OS | Microsoft Windows 11 or later |
| --- | --- |
| Statistical analysis software | R4.2.0 or later |
| Representative software | R and Microsoft Excel |

# **Amendment**

|  | **(Before change) September 30, 2024** |  | **(After change) October 31, 2024** | **Reason for change** |
| --- | --- | --- | --- | --- |
| P.1 | Created on September 30, 2024 (Version 1.0) | P.1 | Created on October 31, 2024 (Version 1.1) | Revision |
| P.2 | **Creation/Revision history** | P.2 | **Creation/Revision history**  (2nd line in the table)  1.1 October 31, 2024 Added description on handling of CAVI data, Takumi Imai | Revision |
| P.5 | **1.2 Definitions of abbreviations** | P.5 | **1.2 Definitions of abbreviations**  (1st line in the table)  ABI ankle brachial index, Ankle-brachial index | This information was needed owing to the revisions |
| P.10 |  | P.10 | **5.4 CAVI data handling**  CAVI data with low ABI values (≤ 0.9) will not be used in the tabulation and analysis owing to its limited reliability. If CAVI data from both sides are available, they will be averaged and used for tabulation and analysis. If CAVI data from only one side are available, that CAVI data will be used for tabulation and analysis. | CAVI data handling was determined based on discussions with experts in CAVI assessment |

**Supplementary Figure S1. Background KDIGO risk categories of patients included in FIVE-STAR trial***

**
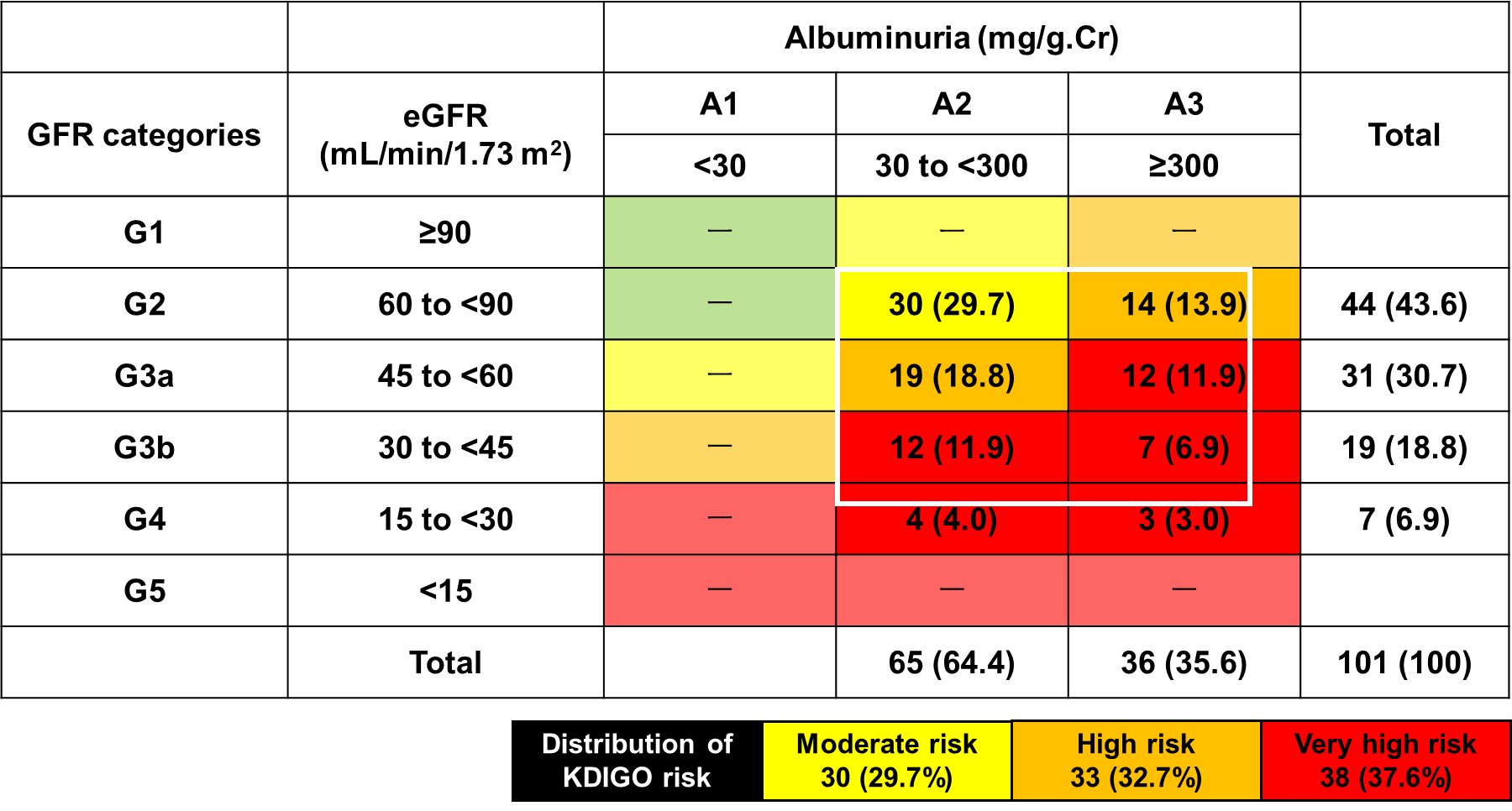
**

Data are expressed as number (percentage).

*This trial included patients with an eGFR ranging ≥25 to <90 mL/min/1.73 m^2^ and elevated albuminuria (UACR ≥30 to <3500 mg/g.Cr). These eGFR and UACR ranges for trial eligibility are shown as a white-line box.

eGFR, estimated glomerular filtration rate; KDIGO, Kidney Disease Improving Global Outcomes; UACR, urinary albumin-to-creatinine ratio.

**Supplementary Figure S2. CAVI response in prespecified subgroups**


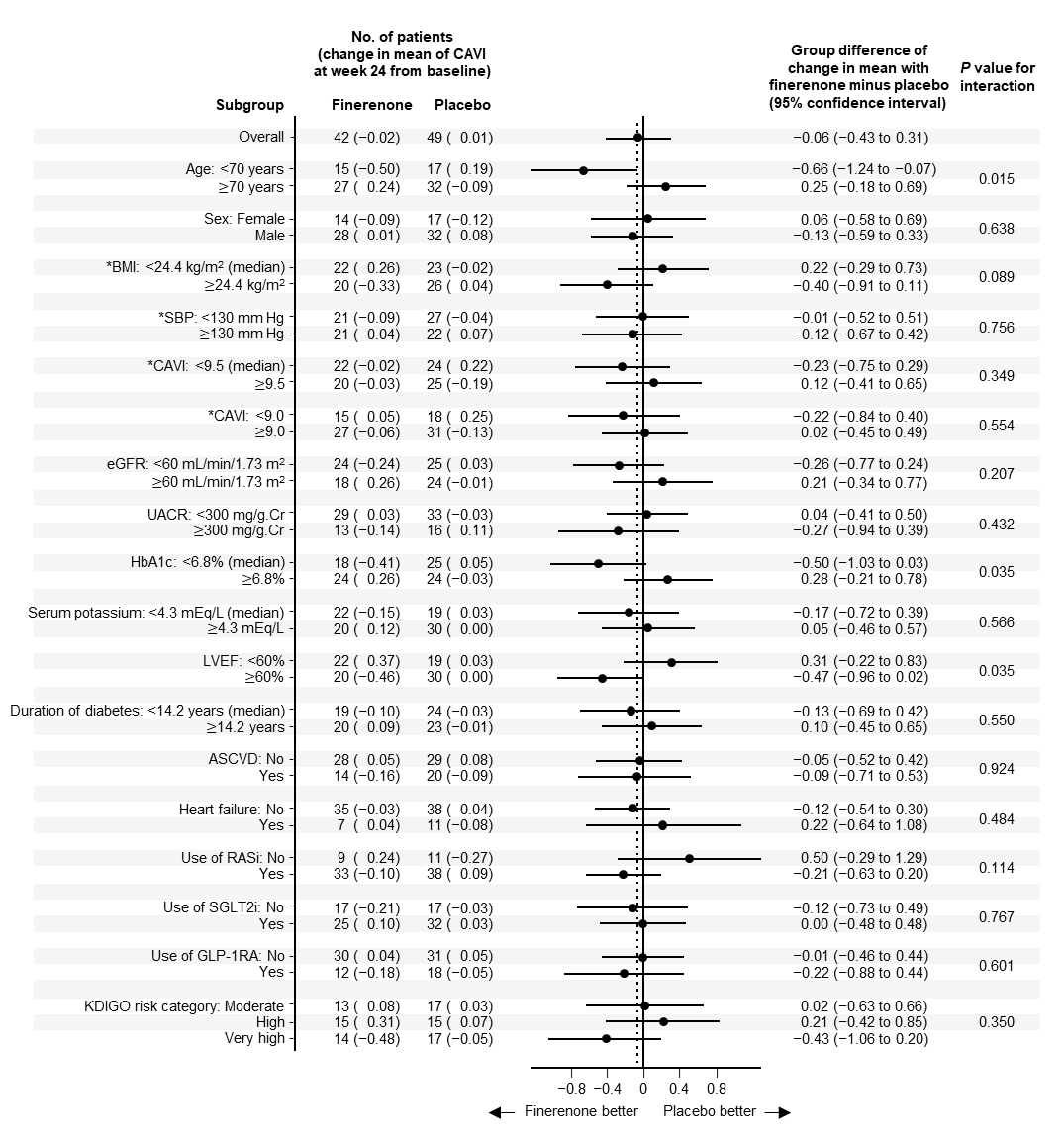


CAVI changes from baseline to week 24 in prespecified subgroups based on background demographics and clinical characteristics.

* At baseline

ASCVD, atherosclerotic cardiovascular diseases (including ischemic heart disease, stroke, or peripheral arterial disease); BMI, body mass index; CAVI, cardio-ankle vascular index; eGFR, estimated glomerular filtration rate; GLP-1RA, glucagon-like peptide-1 receptor agonist; KDIGO, Kidney Disease: Improving Global Outcomes; LVEF, left ventricular ejection fraction; RASi, renin-angiotensin system inhibitor (including angiotensin-converting enzyme inhibitor, angiotensin receptor blocker, or sacubitril/valsartan); SBP, systolic blood pressure; Sac/Val, sacubitril/valsartan; SGLT2i, sodium-glucose co-transporter 2 inhibitor; UACR, urinary albumin-to-creatinine ratio.

**Supplementary Figure S3. Changes in CAVI in a subgroup (age <70 years)**


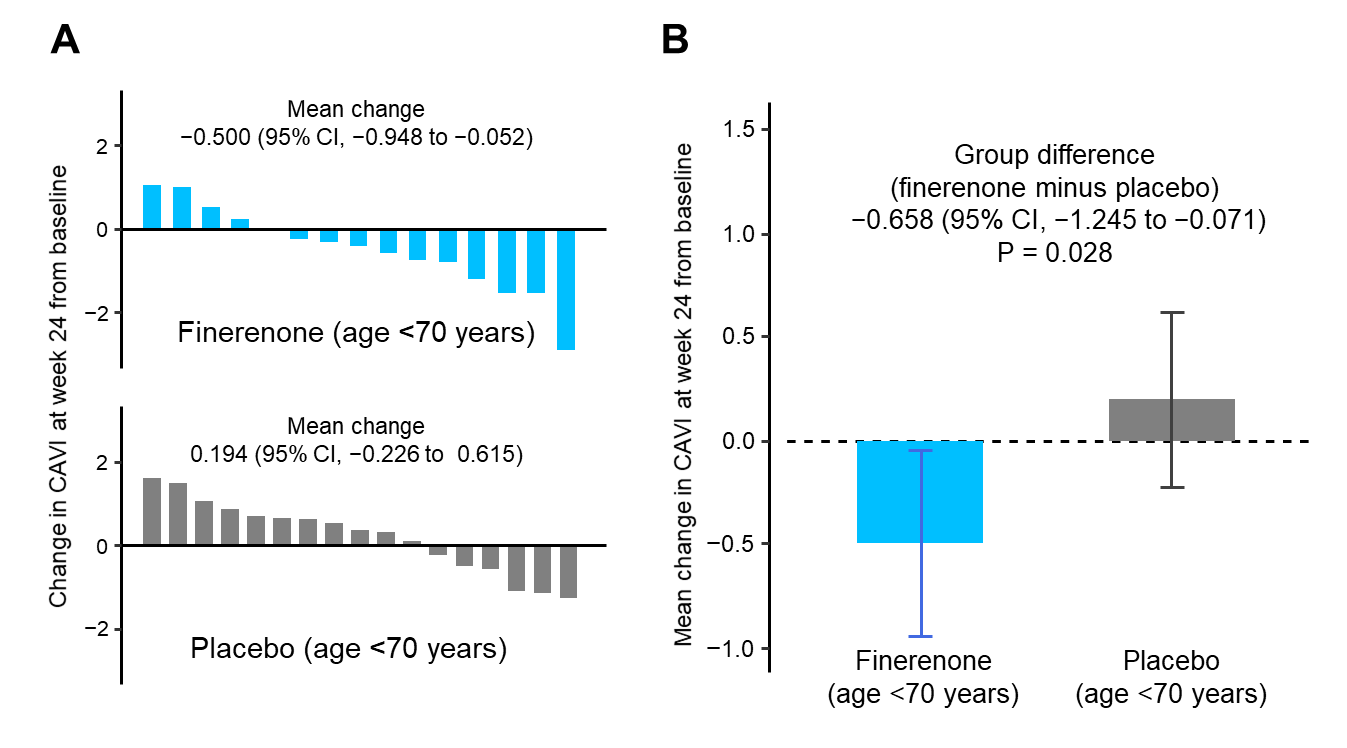


A. Waterfall plots showing changes in CAVI from baseline to week 24 by individual. B. Comparison of changes in CAVI at week 24 from baseline in a subgroup (age <70 years). The group differences (finerenone minus placebo) were estimated with adjustment for baseline CAVI.

CAVI, cardio-ankle vascular index; CI, confidence interval.

**Supplementary Figure S4. Changes in CAVI in a subgroup (HbA1c <6.8%)**


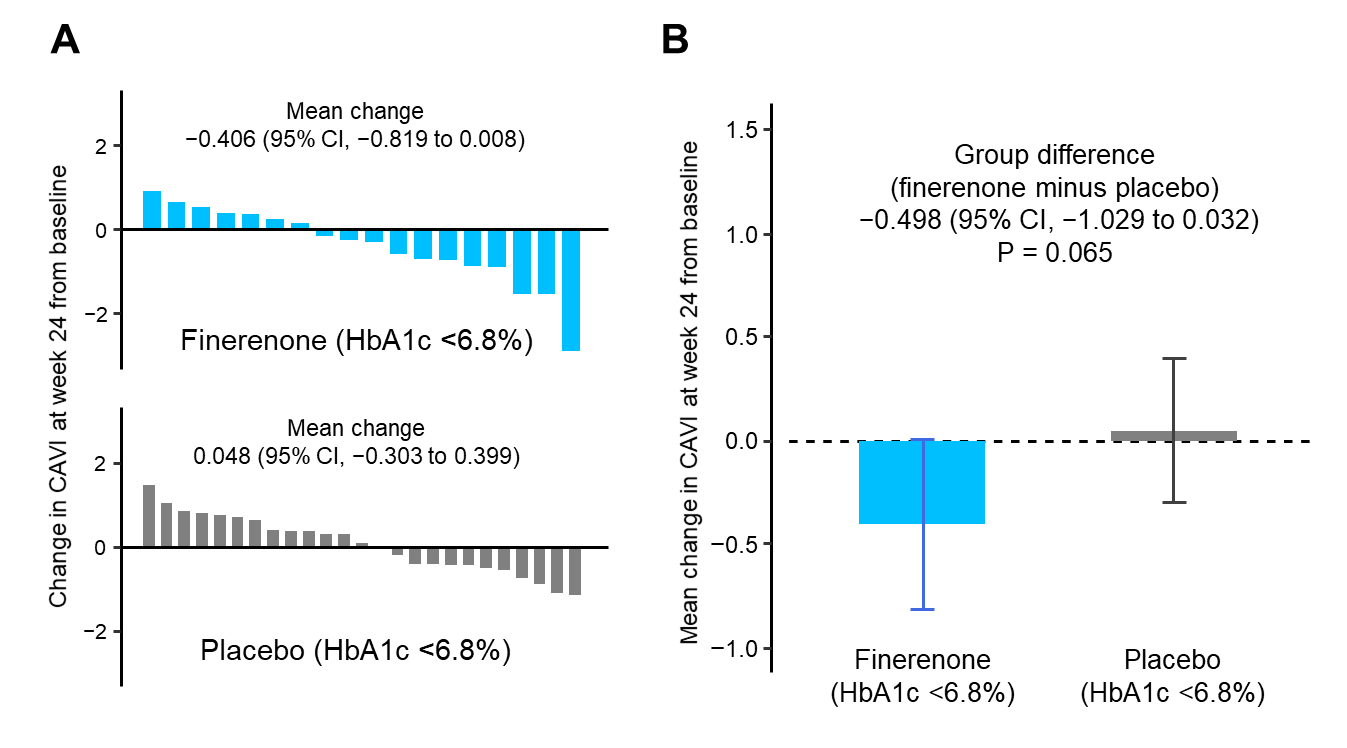


A. Waterfall plots showing changes in CAVI from baseline to week 24 by individual. B. Comparison of changes in CAVI at week 24 from baseline in a subgroup (HbA1c <6.8%). The group differences (finerenone minus placebo) were estimated with adjustment for baseline CAVI.

CAVI, cardio-ankle vascular index; CI, confidence interval.

**Supplementary Figure S5. Changes in CAVI in a subgroup (LVEF ≥60%)**


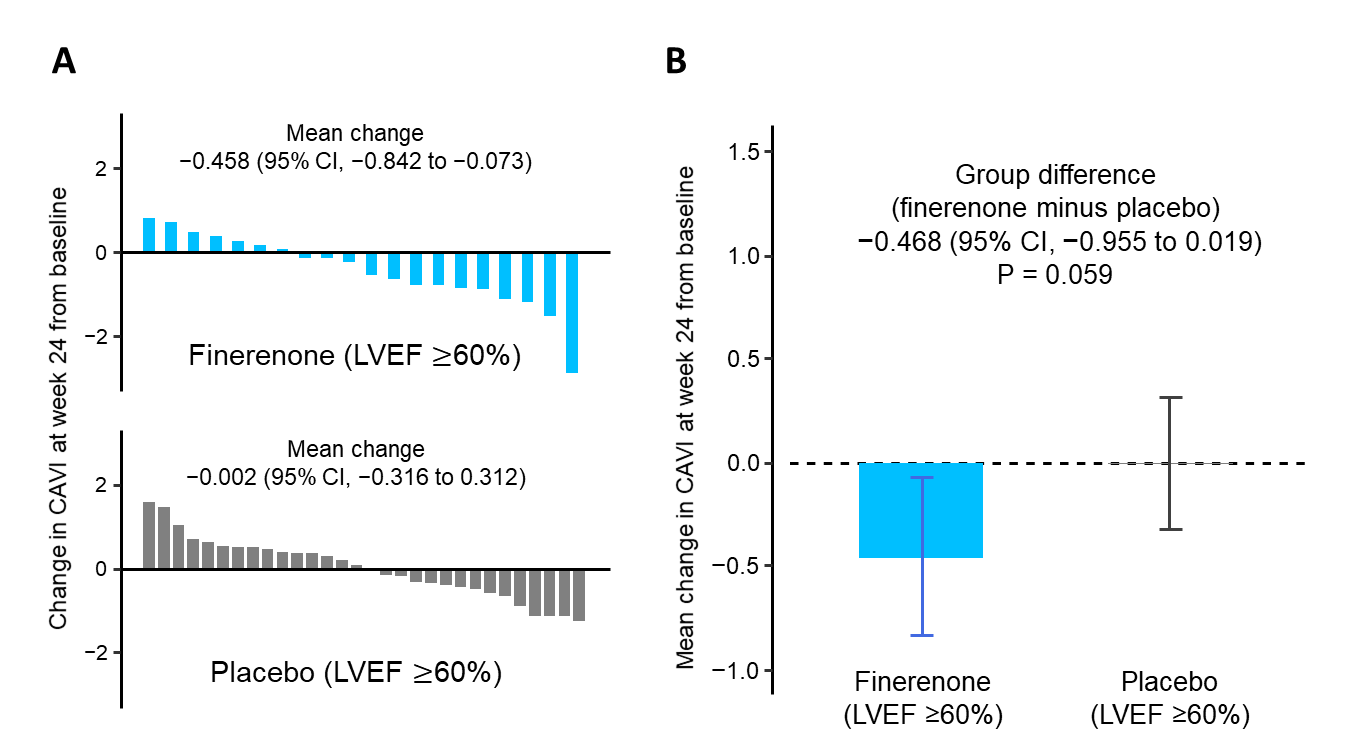


A. Waterfall plots showing changes in CAVI from baseline to week 24 by individual. B. Comparison of changes in CAVI at week 24 from baseline in a subgroup (LVEF ≥60%). The group differences (finerenone minus placebo) were estimated with adjustment for baseline CAVI.

CAVI, cardio-ankle vascular index; CI, confidence interval; LVEF, left ventricular ejection fraction.

**Supplementary Figure S6. Correlation between changes in CAVI and UACR**


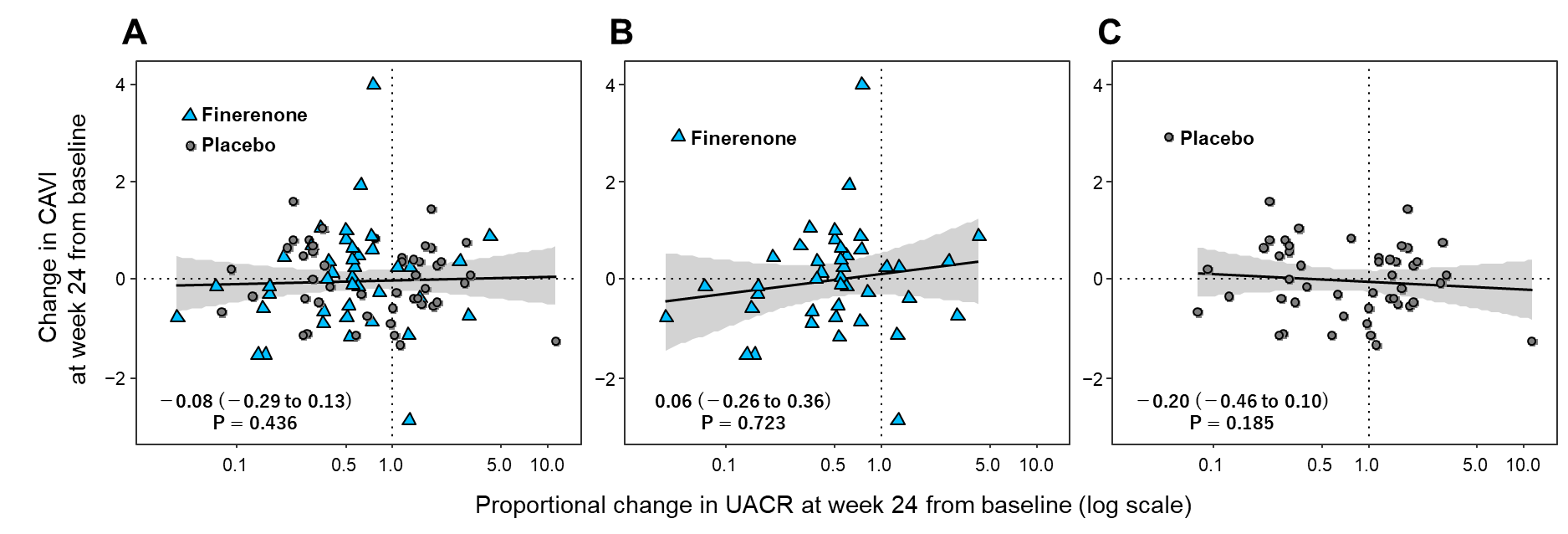


Scatter plot of the change in CAVI versus the proportional change in UACR at week 24 from baseline for all patients (A), the finerenone group (B), and the placebo group (C). No significant correlation was observed between changes in CAVI and UACR (values in figures represent Pearson’s correlation coefficient [95% confidence interval]), and no heterogeneity was identified across the treatment groups (P=0.216).

CAVI, cardio-ankle vascular index; UACR, urinary albumin-to-creatinine ratio.

**Supplementary Figure S7. Focused network of proteins influenced by finerenone with a nominal P value of <0.05.**

**
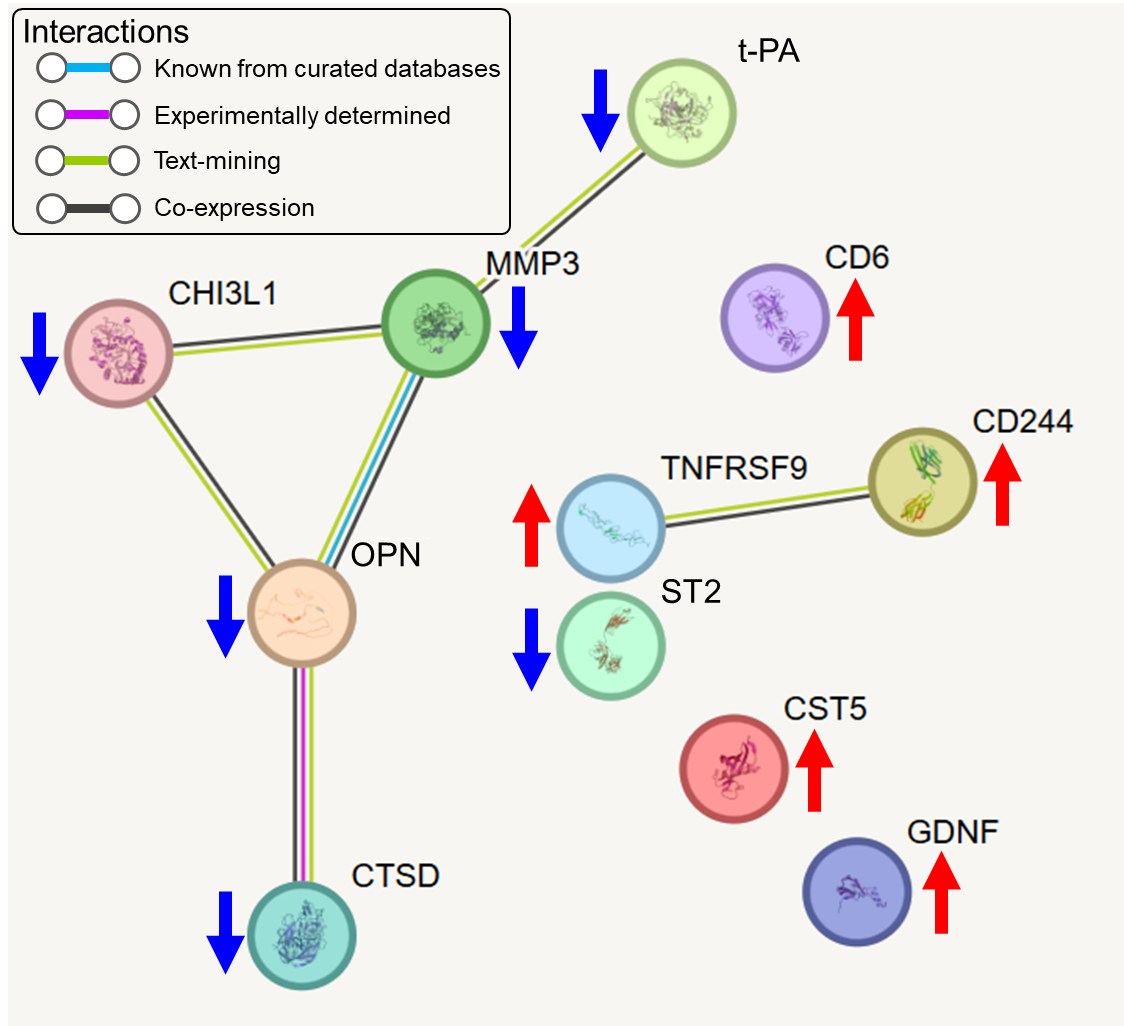
**

Focused network of proteins that were altered with finerenone therapy compared with placebo from baseline to week 24 (nominal P value <0.05).

The network and interactions were defined using STRING.

CHI3LI, chitinase-3 like-protein-1; CST5, cystatin D; CTSD, cathepsin D; GDNF, glial cell line-derived neurotrophic factor; MMP-3, matrix metalloproteinase-3; OPN, osteopontin; STRING, Search Tool for the Retrieval of Interacting Genes/Proteins; ST2, suppression of tumorigenesis-2; TNFRSF9, tumor necrosis factor receptor superfamily member 9; t-PA, tissue-type plasminogen activator.

**Supplementary Table S1. Doses of study drugs in on-treatment patients at week 24**

|  | **Total (*n* = 94)** | **Finerenone (*n* = 47)** | **Placebo (*n* = 47)** |
| --- | --- | --- | --- |
| Initial daily dose at baseline |  |  |  |
| 10 mg | 51 (54.3) | 26 (55.3) | 25 (53.2) |
| 20 mg | 43 (45.7) | 21 (44.7) | 22 (46.8) |
| Final daily dose at week 24 |  |  |  |
| 10 mg | 12 (12.8) | 8 (17.0) | 4 (8.5) |
| 20 mg | 82 (87.2) | 39 (83.0) | 43 (91.5) |

Values are expressed as n (%).

**Supplementary Table S2. Changes in clinical and laboratory measures**

| **Outcome and time** | **Finerenone** | **Placebo** | **Group difference***  **(finerenone minus placebo)** | |
| --- | --- | --- | --- | --- |
|  | **Mean (95% CI)** | **Mean (95% CI)** | **Difference (95% CI)** | ***P* value** |
| Body mass index, kg/m^2^ | (*n* = 51) | (*n* = 50) |  |  |
| At baseline | 25.1 (23.8 to 26.4) | 26.1 (24.8 to 27.4) |  |  |
| At week 4 | 25.0 (23.7 to 26.3) | 26.0 (24.7 to 27.3) |  |  |
| At week 12 | 25.0 (23.7 to 26.3) | 26.0 (24.7 to 27.3) |  |  |
| At week 24 | 24.9 (23.6 to 26.2) | 25.9 (24.6 to 27.2) |  |  |
| Change from baseline at week 4 | −0.1 (−0.3 to 0.1) | −0.1 (−0. 3 to 0.1) | 0.0 (−0.3 to 0.3) | 0.990 |
| Change from baseline at week 12 | −0.1 (−0.3 to 0.1) | −0.04 (−0.2 to 0.2) | −0.1 (−0.4 to 0.2) | 0.614 |
| Change from baseline at week 24 | −0.2 (−0.4 to −0.01) | −0.1 (−0.3 to 0.1) | −0.1 (−0.4 to 0.2) | 0.585 |
| Systolic blood pressure, mm Hg | (*n* = 51) | (*n* = 50) |  |  |
| At baseline | 133.1 (128.4 to 137.8) | 129.9 (125.1 to 134.6) |  |  |
| At week 4 | 128.1 (123.4 to 132.8) | 129.2 (124.5 to 134.0) |  |  |
| At week 12 | 126.8 (122.0 to 131.6) | 134.4 (129.7 to 139.2) |  |  |
| At week 24 | 129.4 (124.6 to 134.2) | 133.9 (129.2 to 138.7) |  |  |
| Change from baseline at week 4 | −5.1 (−9.8 to −0.3) | −0.6 (−5.4 to 4.2) | −2.8 (−8.7 to 3.2) | 0.357 |
| Change from baseline at week 12 | −6.1 (−10.9 to −1.2) | 4.6 (−0.3 to 9.4) | −9.2 (−15.2 to −3.2) | 0.003 |
| Change from baseline at week 24 | −3.5 (−8.3 to 1.3) | 4.1 (−0.8 to 8.9) | −6.1 (−12.1 to −0.1) | 0.045 |
| Diastolic blood pressure, mm Hg | (*n* = 51) | (*n* = 50) |  |  |
| At baseline | 73.6 (70.3 to 77.0) | 74.2 (70.8 to 77.6) |  |  |
| At week 4 | 70.0 (66.7 to 73.4) | 72.5 (69.2 to 75.9) |  |  |
| At week 12 | 69.2 (65.9 to 72.6) | 75.0 (71.7 to 78.4) |  |  |
| At week 24 | 71.0 (67.6 to 74.4) | 74.5 (71.2 to 77.9) |  |  |
| Change from baseline at week 4 | −3.6 (−6.9 to −0.3) | −1.7 (−5.0 to 1.7) | −2.2 (−6.3 to 1.9) | 0.288 |
| Change from baseline at week 12 | −4.4 (−7.7 to −1.0) | 0.8 (−2.5 to 4.2) | −5.5 (−9.6 to −1.4) | 0.009 |
| Change from baseline at week 24 | −2.6 (−6.0 to 0.7) | 0.3 (−3.0 to 3.7) | −3.3 (−7.4 to 0.9) | 0.120 |
| HbA1c, % | (*n* = 49) | (*n* = 50) |  |  |
| At baseline | 7.1 (6.8 to 7.4) | 7.0 (6.8 to 7.3) |  |  |
| At week 12 | 7.2 (7.0 to 7.5) | 7.2 (6.9 to 7.4) |  |  |
| At week 24 | 7.3 (7.0 to 7.5) | 7.2 (6.9 to 7.4) |  |  |
| Change from baseline at week 12 | 0.1 (−0.03 to 0.3) | 0.1 (−0.02 to 0.3) | −0.02 (−0.2 to 0.2) | 0.897 |
| Change from baseline at week 24 | 0.1 (−0.01 to 0.3) | 0.1 (−0.03 to 0.3) | 0.02 (−0.2 to 0.2) | 0.868 |
| Serum potassium, mEq/L | (*n* = 51) | (*n* = 50) |  |  |
| At baseline | 4.2 (4.1 to 4.3) | 4.4 (4.3 to 4.5) |  |  |
| At week 4 | 4.4 (4.3 to 4.5) | 4.3 (4.2 to 4.4) |  |  |
| At week 12 | 4.4 (4.3 to 4.5) | 4.3 (4.2 to 4.4) |  |  |
| At week 24 | 4.4 (4.3 to 4.5) | 4.3 (4.2 to 4.4) |  |  |
| Change from baseline at week 4 | 0.3 (0.1 to 0.4) | −0.1 (−0.2 to 0.02) | 0.3 (0.1 to 0.4) | <0.001 |
| Change from baseline at week 12 | 0.2 (0.1 to 0.3) | −0.1 (−0.2 to 0.1) | 0.2 (0.04 to 0.3) | 0.011 |
| Change from baseline at week 24 | 0.2 (0.1 to 0.3) | −0.1 (−0.2 to 0.04) | 0.2 (0.1 to 0.3) | 0.003 |
| eGFR, mL/min/1.73 m^2^ | (*n* = 51) | (*n* = 50) |  |  |
| At baseline | 57.2 (52.2 to 62.1) | 57.8 (52.8 to 62.8) |  |  |
| At week 4 | 53.9 (48.9 to 58.8) | 59.1 (54.1 to 64.1) |  |  |
| At week 12 | 52.5 (47.5 to 57.5) | 58.8 (53.8 to 63.8) |  |  |
| At week 24 | 52.4 (47.4 to 57.3) | 57.3 (52.3 to 62.3) |  |  |
| Change from baseline at week 4 | −3.3 (−5.3 to −1.3) | 1.3 (−0.8 to 3.3) | −4.6 (−7.4 to −1.9) | 0.001 |
| Change from baseline at week 12 | −4.7 (−6.7 to −2.7) | 1.0 (−1.0 to 3.0) | −5.8 (−8.6 to −3.0) | <0.001 |
| Change from baseline at week 24 | −4.8 (−6.9 to −2.8) | −0.5 (−2.5 to 1.6) | −4.4 (−7.2 to −1.6) | 0.002 |
| Serum Cystatin C, | (*n* = 48) | (*n* = 48) |  |  |
| At baseline | 1.3 (1.2 to 1.4) | 1.3 (1.2 to 1.4) |  |  |
| At week 12 | 1.4 (1.3 to 1.5) | 1.2 (1.1 to 1.4) |  |  |
| At week 24 | 1.4 (1.3 to 1.5) | 1.3 (1.2 to 1.4) |  |  |
| Change from baseline at week 12 | 0.1 (0.03 to 0.2) | −0.03 (−0.1 to 0.05) | 0.1 (0.03 to 0.2) | 0.008 |
| Change from baseline at week 24 | 0.1 (0.05 to 0.2) | 0.05 (−0.02 to 0.1) | 0.07 (−0.02 to 0.2) | 0.131 |
| Plasma/serum aldosterone concentration, pg/mL† | (*n* = 49) | (*n* = 48) |  |  |
| At baseline | 22.2 (16.6 to 29.7) | 29.7 (22.0 to 40.0) |  |  |
| At week 24 | 34.9 (25.7 to 47.4) | 30.3 (22.4 to 41.0) |  |  |
| Proportional change at week 24 | 1.6 (1.3 to 1.9) | 1.0 (0.8 to 1.2) | 1.5 (1.2 to 1.9)‡ | 0.001 |
| Plasma/serum renin activity, ng/mL/hr† | (*n* = 45) | (*n* = 45) |  |  |
| At baseline | 2.9 (2.0 to 4.1) | 2.5 (1.8 to 3.6) |  |  |
| At week 24 | 4.9 (3.4 to 6.9) | 2.5 (1.8 to 3.5) |  |  |
| Proportional change at week 24 | 1.7 (1.3 to 2.2) | 1.0 (0.7 to 1.3) | 1.8 (1.3 to 2.5)‡ | 0.001 |

* Adjusted for baseline values

† Data are expressed as the geometric means (95% CI) or proportional change from baseline (95% CI).

‡ Group ratio (finerenone vs. placebo), adjusted for baseline values

CI, confidence interval; eGFR, estimated glomerular filtration rate.

**Supplementary Table S3. Effect on circulating proteins from Cardiovascular III and Inflammation panels**

| **Protein** | **Panel** | **Major function** | **Effect estimate (finerenone minus placebo)*** | **Nominal *P* value** | **BH *P* value** |
| --- | --- | --- | --- | --- | --- |
| CST5 | Inflammation | Inhibition of cysteine proteinases | 0.188569 | 0.000774 | 0.142379 |
| GDNF | Inflammation | Enhancement of survival and morphological differentiation of dopaminergic neurons | 0.194652 | 0.006729 | 0.417406 |
| OPN | Cardiovascular III | Cell-matrix interaction; Inflammatory response | −0.245702 | 0.006806 | 0.417406 |
| CD244 | Inflammation | Inflammation: Immune response | 0.117030 | 0.019069 | 0.559225 |
| CHI3L1 | Cardiovascular III | Angiogenesis; Inflammatory response | −0.323429 | 0.022454 | 0.559225 |
| t-PA | Cardiovascular III | Antithrombotic action | −0.227057 | 0.023265 | 0.559225 |
| MMP-3 | Cardiovascular III | Degradation of cartilage and connective tissue | −0.206002 | 0.024168 | 0.559225 |
| ST2 | Cardiovascular III | Inflammation; Cell proliferation; Fibrosis | −0.169734 | 0.024463 | 0.559225 |
| CTSD | Cardiovascular III | Angiogenesis; Inflammatory response | −0.131026 | 0.028905 | 0.559225 |
| TNFRSF9 | Inflammation | Cell survival and differentiation; Immunity | 0.142584 | 0.030393 | 0.559225 |
| CD6 | Inflammation | Cell adhesion; T-cell activation | 0.124113 | 0.034674 | 0.579999 |

Six proteins (OPN, CHI3L1, t-PA, MMP-3, ST2, and CTSD) were downregulated (blue cells) with finerenone therapy compared with placebo, and five proteins (CST5, GDNF, CD244, TNFRSF9, and CD6) were upregulated (red cells).

* Based on log2 fold change from baseline in normalized protein expression at week 24.

BH, Benjamini-Hochberg method; CHI3LI, chitinase-3 like-protein-1; CST5, cystatin D; CTSD, cathepsin D; GDNF, glial cell line-derived neurotrophic factor; MMP-3, matrix metalloproteinase-3; OPN, osteopontin; ST2, suppression of tumorigenesis-2; TNFRSF9, tumor necrosis factor receptor superfamily member 9; t-PA, tissue-type plasminogen activator.

**Supplementary Table S4. Safety endpoints***

| **Event** | **Finerenone**  **(*n* = 51)** | **Placebo**  **(*n* = 50)** |
| --- | --- | --- |
| Any adverse event | 8 (15.7) | 8 (16.0) |
| Any serious adverse event | 5 (9.8) | 5 (10.0) |
| Atrial fibrillation | 0 | 1 (2.0) |
| Cardiac failure | 0 | 1 (2.0) |
| Cataract traumatic | 0 | 1 (2.0) |
| Cholangitis acute | 1 (2.0) | 0 |
| COVID-19 | 1 (2.0) | 0 |
| Gastric cancer | 0 | 1 (2.0) |
| Interstitial lung disease | 1 (2.0) | 0 |
| Large intestinal polypectomy | 0 | 1 (2.0) |
| Pyelonephritis | 1 (2.0) | 0 |
| Thermal burn | 1 (2.0) | 0 |
| Documented serum potassium level† |  |  |
| >5.5 mEq/L | 0 | 0 |
| <3.5 mEq/L | 0 | 3 (6.0) |
| Investigator-reported hyperkalaemia | 1 (2.0) | 0 |
| Adverse event related to study drugs ‡ | 2 (3.9) | 1 (2.0) |
| Serious adverse event related to study drugs ‡ | 1 (2.0)§ | 0 |
| Adverse event leading to discontinuation of study drugs | 2 (3.9)¶ | 1 (2.0)‖ |

* Shown are adverse events (n [%]) that occurred after the initiation of the study drug. Serious adverse events were classified according to the preferred terms from the Medical Dictionary for Regulatory Activities.

† Data are counted based on laboratory results from weeks 4, 12, or 24.

‡ A causal relationship between any adverse event and study drug administration was based on the opinion of the reporting investigators.

§ Interstitial lung disease (interstitial pneumonia)

¶ Serious adverse events (interstitial lung disease and pyelonephritis)

‖ Non-serious adverse event (abnormal hepatic function)
